# Supplementary material for: Early stages of covalent organic framework formation imaged in operando
Source: Nature. 2024 Jun 5;630(8018):872–7. doi: 10.1038/s41586-024-07483-0 (PMC11208157; doi:10.1038/s41586-024-07483-0)
Supplement: Supplementary file 1 — This file contains Supplementary Discussions about the iSCAT methodology, the chosen experimental conditions, COF crystallization, surfactant-free solvent structuring and a comparison of iSCAT to other techniques used at present for monitoring wet-chemical reactions. Supplementary iSCAT images are provided of solvent-restructuring processes, as well as of COF formation in various catalyst concentrations and solvent systems. An overview is provided of solvent systems used at present for COF synthesis and solvent systems that have the potential to be used in the future. Supplementary (in situ) XRD data are provided for the COF reaction mechanism in structured/unstructured solvents and the broad applicability of the IAC protocol. This file contains Supplementary Figs. 1–60, Supplementary Tables 1–5 and a guide to Supplementary Videos 1–10. [file 41586_2024_7483_MOESM1_ESM.pdf]

nature portfolio

https://doi.org/10.1038/s41586-024-07483-0

Supplementary information

Early stages of covalent organic framework formation imaged in operando

In the format provided by the authors and unedited

Supplementary Information for

Early stages of covalent organic framework formation imaged in operando

Christoph G. Gruber[1], Laura Frey[2], Roman Guntermann[2], Dana D. Medina[2*], Emiliano Cortés[1*]

[1] Nanoinstitute Munich and Center for Nanoscience (CeNS), Faculty of Physics, Ludwig-Maximilians-Universität München, 80539 Munich, Germany. e-mail: emiliano.cortes@lmu.de

[2] Department of Chemistry and Center for Nanoscience (CeNS), Ludwig-Maximilians-Universität München, 81377 Munich, Germany. e-mail: dana.medina@cup.lmu.de

Contents

Supplementary video guide 4
Section 1. Comparison of iSCAT with current techniques for mechanistic studies on COF formation dynamics 7
Section 2. Synthesis conditions chosen for the mechanistic studies (3M, RT) 10
a) Temperature 10
b) Catalyst concentration 10
c) Confirmation of imine bond and COF formation under iSCAT conditions 20
d) Optical absorption 23
Section 3. iSCAT methodology 24
a) iSCAT signal origin and contributions 24
b) Background correction and image processing 25
c) Acronyms for microscopes exploiting interferometric detection 27
d) StdDev as figure of merit for temporal iSCAT traces 27
e) Differentiation between scatterers floating in solution and attached on the substrate 27
f) Size estimation based on iSCAT contrast 28
Section 4. COF crystallization mechanism in current literature 29
Section 5. Refractive index of binary and ternary solvent systems in the TA-TAPB COF reaction 30
Section 6. Reflectivity of solvents and solvent mixtures in iSCAT 31
Section 7. Solvent restructuring processes visualized with iSCAT 32
Section 8. Droplet growth during liquid-liquid phase rearrangements 37
Section 9. Surfactant-free solvent structuring and liquid phase diagrams 40
Section 10. iSCAT measurements of COF formation in a binary solvent system without solvent structuring 42
Section 11. Solvent systems meeting the conditions for surfactant-free emulsification commonly used in COF synthesis and systems currently not used in COF synthesis 45
Section 12. iSCAT induction periods of TA TAPB COF formation employing different catalyst and solvent conditions 47
Section 13. Complementary data to Figures 3 and 4 49
a) Corresponding PXRD patterns to Figure 2b 49
b) Effect of the inclusion of mesitylene in the reaction mixture at different reaction times 49
c) Corresponding PXRD patterns to the crystallinity plot in Figure 3c 50
d) iSCAT induction periods of TA-TAPB COF formation 51
e) 100 Peak height as Figure of merit for trend shown in Fig. 4a 51
Section 14. Effect of including NaCl into the catalyst mixture on the solvent restructuring processes 52
Section 15. Characterization of TA TAPB COF synthesized with IAC approach at RT 58
a) Indexing of TA-TAPB COF obtained with 3M HOAc/1.5M NaCl as catalyst mixture at RT 58
b) Pore accessibility of TA-TAPB COF synthesized with 3M HOAc/1.5M NaCl as catalyst mixture at RT 58
c) Morphology of TA-TAPB COF synthesized with NaCl in catalyst mixture 59

Section 16. IAC approach - required conditions, generalizability, upscaling and effect on the COF crystallization ..60
a) Effect of NaCl addition on COF crystallinity in a binary solvent system...60
b) Effect of NaCl addition on COF crystallinity depending on introduction time 60
c) Relative catalyst mixture concentrations 61
d) Ions...62
e) Gram-scale synthesis 62
f) In-situ XRD analysis of TA-TAPB COF reaction with and without NaCl 63
Section 17. Expanding the concept to different COFs, solvent systems, and salt types 68
a) WTA COF, inorganic salt...69
b) TAPB-DMTA COF, inorganic salt 70
c) TT-ETTA COF, inorganic salt...71
d) WTA COF, antagonistic salt 72
References 73

Supplementary video guide

Supplementary Video 1.

iSCAT video of TA-TAPB COF formation with 3M HOAc as catalyst (room temperature; 1,4-dioxane/mesitylene/HOAc 9:1:1 v/v). After addition of the catalyst (start of timer), phase rearrangement processes are imaged (ca. 0 – 2 sec). Here, nucleation, growth, and dissolution of black contrast mesitylene droplets are imaged. This is followed by nucleation, precipitation and growth of the COF framework (ca. 2 – 80 sec). Images were acquired at a speed of 2.7 ms per frame (370 fps), background-subtracted, 2x2 binned and temporally averaged (5 consecutive frames) resulting in an effective temporal resolution of 13.5 ms. The video speed is increased, so that 1 second in this video equals 9 seconds in real-time (total real-time of video: 90 sec). The contrast is adjusted to 0.66 – 2.0.

Supplementary Video 2.

iSCAT video of the phase rearrangement after catalyst addition during TA-TAPB COF formation with 3M HOAc (room temperature; 1,4-dioxane/mesitylene/HOAc 9:1:1 v/v). After addition of the catalyst (start of timer), nucleation, growth, and dissolution of black contrast mesitylene droplets are imaged (ca. 0 – 2 sec). Images were acquired at a speed of 2.7 ms per frame (370 fps), background-subtracted and 2x2 binned. The video is slowed down, so that 1 second in this video equals 270 ms in real-time (total real-time of video: 2.7 sec). The contrast is adjusted to 0.6 – 3.84.

Supplementary Video 3.

iSCAT video of TA-TAPB COF formation with 6M HOAc as catalyst (room temperature; 1,4-dioxane/mesitylene/HOAc 9:1:1 v/v). After addition of the catalyst (start of timer), growth of the COF framework is imaged. Images were acquired at a speed of 21.8 ms per frame (46 fps), background-subtracted and 2x2 binned. The video speed is increased, so that 1 second in this video equals 21.8 seconds in real-time (total real-time of video: 218 sec). The contrast is adjusted to 0.47 – 1.9.

Supplementary Video 4.

iSCAT video of TA-TAPB COF formation with 1M HOAc as catalyst (room temperature; 1,4-dioxane/mesitylene/HOAc 9:1:1 v/v). After addition of the catalyst (start of timer), phase rearrangement processes are imaged (ca. 0 – 77 sec). Here, nucleation, growth, and dissolution of black contrast mesitylene droplets are imaged. Afterwards, nucleation, precipitation and growth of the COF framework is observed. Images were acquired at a speed of 6.7 ms per frame (150 fps), background-subtracted and 2x2 binned. The video speed is increased, so that 1 second in this video equals 33.5 seconds in real-time (total real-time of video: 335 sec). The contrast is adjusted to 0.5 – 2.53.

Supplementary Video 5.

iSCAT video of in-solution growth during TA-TAPB COF formation with 4M HOAc (room temperature; 1,4-dioxane/mesitylene/HOAc 9:1:1 v/v). After addition of catalyst (start of timer) and an induction period, fluctuations originate from in-solution growth and grow in signal intensity. Images were acquired at a speed of 2.05 ms per frame (488 fps), background-subtracted and 2x2 binned. The video speed is increased, so that 1 second in this video equals 10.3 seconds in real-time (total real-time of video: 103 sec). The contrast is adjusted to 0.89 – 1.62.

Supplementary Video 6.

iSCAT video of the initial attachment and growth of solid matter during TA-TAPB COF formation with 3M HOAc (room temperature; 1,4-dioxane/mesitylene/HOAc 9:1:1 v/v). Images were acquired at a speed of 2.7 ms per frame (370 fps), background-subtracted, 2x2 binned and temporally averaged (5 consecutive frames) resulting in an effective temporal resolution of 13.5 ms. The video is slowed down, so that 1 second in this video equals 540 ms in real-time (total real-time of video: 5.4 sec). The contrast is adjusted to 0.52 – 2.29.

Supplementary Video 7.

iSCAT video of the attachment of a single nanoscale particle from solution during TA-TAPB COF formation with 4M HOAc (room temperature; 1,4-dioxane/mesitylene/HOAc 9:1:1 v/v). The presence of a black spot in the upper-left region of the image is due to a hot pixel defect in the camera. Images were acquired at a speed of 2.05 ms per frame (488 fps), background-subtracted and 2x2 binned. The video is slowed down, so that 1 second in this video equals 540 ms in real-time (total real-time of video: 8.2 sec). The contrast is adjusted to 0.79 – 1.5.

Supplementary Video 8.

iSCAT video of the growth of a single particle that attached directly after acid addition during TA-TAPB COF formation with 3M HOAc (room temperature; 1,4-dioxane/mesitylene/HOAc 9:1:1 v/v). The particle turns from black/negative contrast (interferometric scattering) to white/positive contrast (pure scattering). Images were acquired at a speed of 2.7 ms per frame (370 fps), background-subtracted, 2x2 binned and temporally averaged (10 consecutive frames) resulting in an effective temporal resolution of 27 ms. The video speed is increased, so that 1 second in this video equals 5.4 seconds in real-time (total real-time of video: 54 sec). The contrast is adjusted to 0.68 – 1.67.

Supplementary Video 9.

iSCAT video of TA-TAPB COF formation with 3M HOAc as catalyst in the binary, unstructured solvent system 1,4-dioxane/aqueous HOAc (9:1 v/v; room temperature). Upon the addition of the catalyst (start of timer), diffuse alterations in the reflectivity are observed (ca. 0 – 0.6 sec), stemming from the introduction of water (higher refractive index difference to the glass surface compared to 1,4-dioxane). This effect persists until the solvents are mixed. However, no phase separation phenomena or nucleation of liquid droplets is captured by the imaging process. During and following this phase, the nucleation, precipitation and growth of the COF framework commences (ca. 0.6 – 2 sec). Images were acquired at a speed of 2.2 ms per frame (455 fps), background-subtracted and 2x2 binned. The video is slowed down, so that 1 second in this video equals 220 ms in real-time (total real-time of video: 2.2 sec). The contrast is adjusted to 0.55 – 1.75.

Supplementary Video 10.

iSCAT video of TA-TAPB COF reaction at the oil-water interface in a model system (room temperature; 1,4-dioxane/mesitylene/0.06M HOAc 9:1:5 v/v; 200 µl total volume). Increasing iSCAT signal fluctuations are detected at the interface of oil- and water-rich phase (see Extended Data Fig. 5a for initial condition) resulting from the emergence of solid phase from a reaction at the interface. Images were acquired at a speed of 6.05 ms per frame (165 fps) and background-subtracted (here, as reference background the image shown in Extended Data Fig. 5a was taken). The video is slowed down, so that 1 second in this video equals 3 sec in real-time (total real-time of video: 30 sec). The contrast is adjusted to 0.38 – 1.91.

Section 1. Comparison of iSCAT with current techniques for mechanistic studies on COF formation dynamics

The following tables were created to the best of our knowledge. The values for temporal/lateral resolution as well as the requirements of the techniques listed can strongly depend on the measured sample. We assumed here the monitoring of wet-chemical reactions, similar to COF formation (polymerization and nanometer-sized seed nucleation in liquid).

Direct imaging

	In-Situ Scanning Tunneling Microscopy (STM)	Liquid-Phase transmission electron microscopy (LP-TEM)	iSCAT
**Radiation or illumination**	- (electron tunneling current)	Electrons	Visible Light (400-800 nm)
**Invasiveness**	Non-invasive	Invasive (Electron beam induced radiaion damage)[60,61]	Non-invasive (in absence of optical absorption)
**Operating principle**	The operating principle is based on the quantum tunneling effect. A sharp metallic tip is brought close to a conductive sample surface. Electrons can tunnel between the tip and the surface (when the tip is positioned within a few angstroms of the sample). The tunneling current depends exponentially on the tip-surface distance. This allows for precise control. The tip is scanned across the sample surface while the tunneling current is monitored. By this, a topographic image of the surface can be generated with atomic resolution.[62]	The operating principle is based on the transmission of electrons through the sample. For liquid-phase TEM, a specialized liquid cell with thin electron-transparent windows is utilized. The electron beam passes through both the sample and the liquid. The occurring interactions provide information about the structure and the behavior of the sample in a liquid medium.[63]	The operating principle is based on the interference of light scattered by the sample with a reference beam. The incident light illuminates a sample located on a substrate. The sample can be located in any medium. The scattered light by the sample and the reflected light (=reference beam) by the substrate-medium interface are collected. The two contributions interfere, giving rise to the iSCAT signal at the detector.[5]
**Typical lateral resolution**	Atomic resolution (scan area up to 100 nm)[12]	nm[64]	Ca. 200 – 300 nm (sub-5 nm localization precision)
**Typical acquisition speed**	min[65]	ms/sec[63]	µs/ms
**Probe/ Imaging region**	Flat surfaces	Thin liquid sample layer (typically 1-1.5 µl liquid)[64]	Typically, ca. 300 nm depth[66]
**Sample requirements (for monitoring polymerization)**	• Electronically conductive sample in contact with a conductive substrate[12]<br>• Constraints in sample height modulations[62]	• Generally applicable<br>• Thin liquid sample layer (typically 1-1.5 ul liquid)[64]<br>• Establishment of safe electron dose levels – sample radiation damage, beam effects on solvents	• Generally applicable<br>• Absence of optical absorption<br>• Bulk possible
**Measurement Conditions**	Ambient conditions[65]	Ambient conditions (extendable by sample holder design)	Ambient conditions (extendable by sample holder design)

Supplementary Table 1. Overview of limitations and advantages of techniques applied for direct imaging of wet-chemical formation processes in the COF field.

	Indirect monitoring			
	**Mass spectrometry**	**Sorption measurements**	**In-Situ (Synchrotron) XRD**	**In-Situ Optical Turbidity measurements**
**Radiation or illumination**	- (m/z of ionized sample)	- (Gas ad/desorption)	(Synchrotron) X-Rays	Near-IR Light (typically: 700-1000 nm)
**Invasiveness**	Decomposes samples	Non-invasive	Minimal invasive	Non-invasive
**Operating principle**	The operating principle is based on measuring the mass-to-charge ratio (m/z) of ions. The sample is ionized to create charged particles. These ions are then separated based on their mass-to-charge ratio in a mass analyzer. The subsequently detected mass spectrum provides information about the sample´s molecular composition.[67]	The operating principle is based on the ad-/desorption of nitrogen gas on the material's surface and within its pores. The amount of gas is measured. By this, the surface area and pore size distribution of the material can be obtained.[12]	The operating principle is based on the interaction of X-rays with crystalline/ordered structures. Incident X-rays are diffracted by the crystal lattice. The resulting diffraction pattern gives, among others, insight into the crystal structure and lattice parameters of the material.[68]	The operating principle is based on the interaction of light with a solute sample. A light beam is incident on the sample. The amount of light scattered or absorbed by the sample (e.g., nanoparticles in solution) is measured using a detector (typically at 90 ° and 180°, respectively). The measured intensity is directly related to the samples' turbidity.[69,70]
**Typical lateral resolution**	-	-	-	-
**Typical acquisition speed**	ex situ[19]	ex situ (h[12])	min (lab-scale); ca. 10 sec (synchrotron) [13,18,71]	sec[69]
**Probe/ Imaging region**	Bulk solution[19]	Bulk powder	Bulk solution	Bulk solution
**Sample requirements (for monitoring polymerization)**	• Must be ionizable<br>• Preferably solute[19,67]	• Porous matter<br>• ca. 50 mg solid material [12]	• Crystalline matter<br>• Beam-time at a synchrotron	• Solutes (transparent/slightly colored)<br>• Absorption features of solvents, educts and products need to be separated in order to follow one specific[69]
**Measurement Conditions**	Ionized Gas phase	Vacuum, 77 K[12]	Ambient conditions (extendable by sample holder design)	Ambient conditions (extendable by sample holder design)

Supplementary Table 2. Overview of limitations and advantages of techniques applied for indirect monitoring of wet-chemical formation processes in the COF field.

Section 2. Synthesis conditions chosen for the mechanistic studies (3M, RT)

a) Temperature

The decision to conduct the experiments at room temperature instead of elevated temperature (120 °C) was based on the time scale of the processes under investigation in this study, such as solvent mixing, initial reaction stages, and polymerization, which occur within the range of milliseconds to minutes.
Within the time scales of the processes studied, the reaction mixture in bulk synthesis is maintained at room temperature during the preparation stage. It is only after the preparation that the mixture is transferred to an oven to undergo heating up to a temperature of 120 °C. This sequential approach ensures that the initial stages of the reactions occur at room temperature before subjecting the mixture to elevated temperatures.
Additional experimental verification and details can be found in Supplementary Section 3c.

b) Catalyst concentration

Various catalyst concentrations were evaluated, including 1M, 3M, and 6M HOAc. The conducted iSCAT measurements confirmed that regardless of the catalyst concentration used, the same sequence of reaction stages was observed and imaged, albeit with altered kinetics (see Supplementary Figure 2 - 12).
While for high catalyst concentration some processes are masked because of overlapping timelines (e.g., nucleation and growth for 6M HOAc), for low catalyst concentration (e.g., for 1M HOAc) these processes are comparably slow. Accordingly, we chose 3M HOAc as the ideal condition to access all stages of COF emergence in one measurement. Supplementary Figure 1 provides visual evidence supporting this decision.

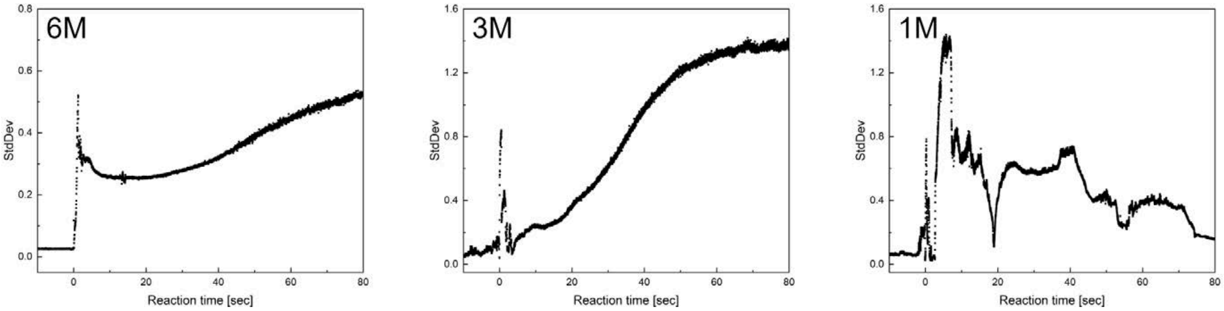

Supplementary Figure 1. A comparative analysis of the temporal evolution of the iSCAT signal was conducted for varying concentrations of acetic acid (6M, 3M, and 1M) over a period of 90 seconds. In the case of 3M HOAc, the phase rearrangement processes as well as the growth processes can be clearly distinguished. The reaction kinetics for 6M HOAc are faster, effectively masking the phase rearrangement phenomena. In contrast, the slower reaction kinetics of 1M HOAc only permitted the observation of phase rearrangements during the 90-second timeframe. Images for 1M, 3M, 6M, were taken at 6.5 ms (154 fps), 2.7 ms (370 fps), 21.8 ms (46 fps) per frame, respectively.

6M HOAc:

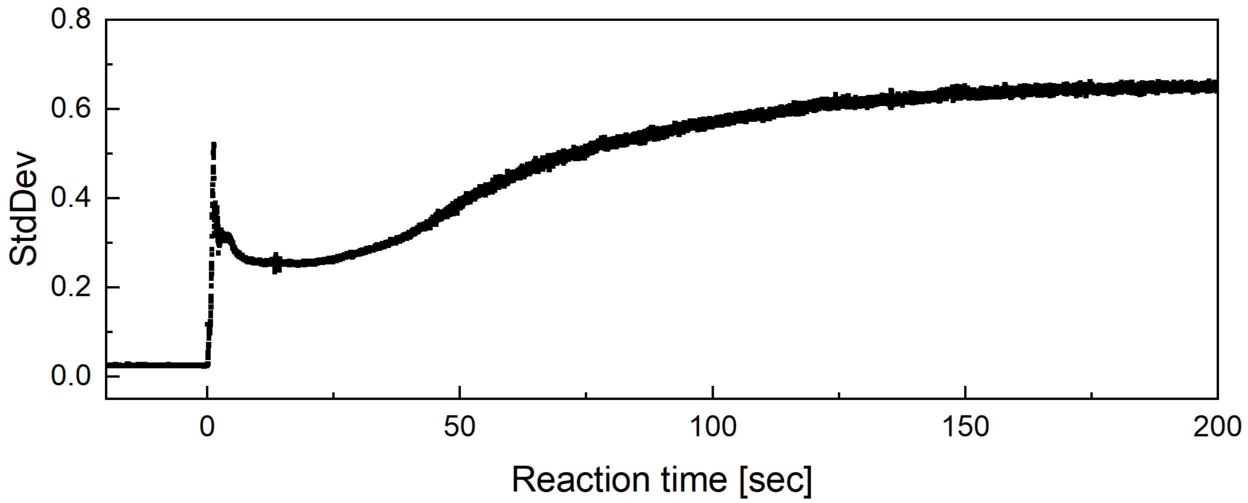

Supplementary Figure 2. Temporal evolution of the StdDev of the iSCAT images acquired during the formation of TA-TAPB COF with 6M HOAc. Images were acquired at a speed of 21.8 ms per frame (46 fps), background-subtracted and 2x2 binned.

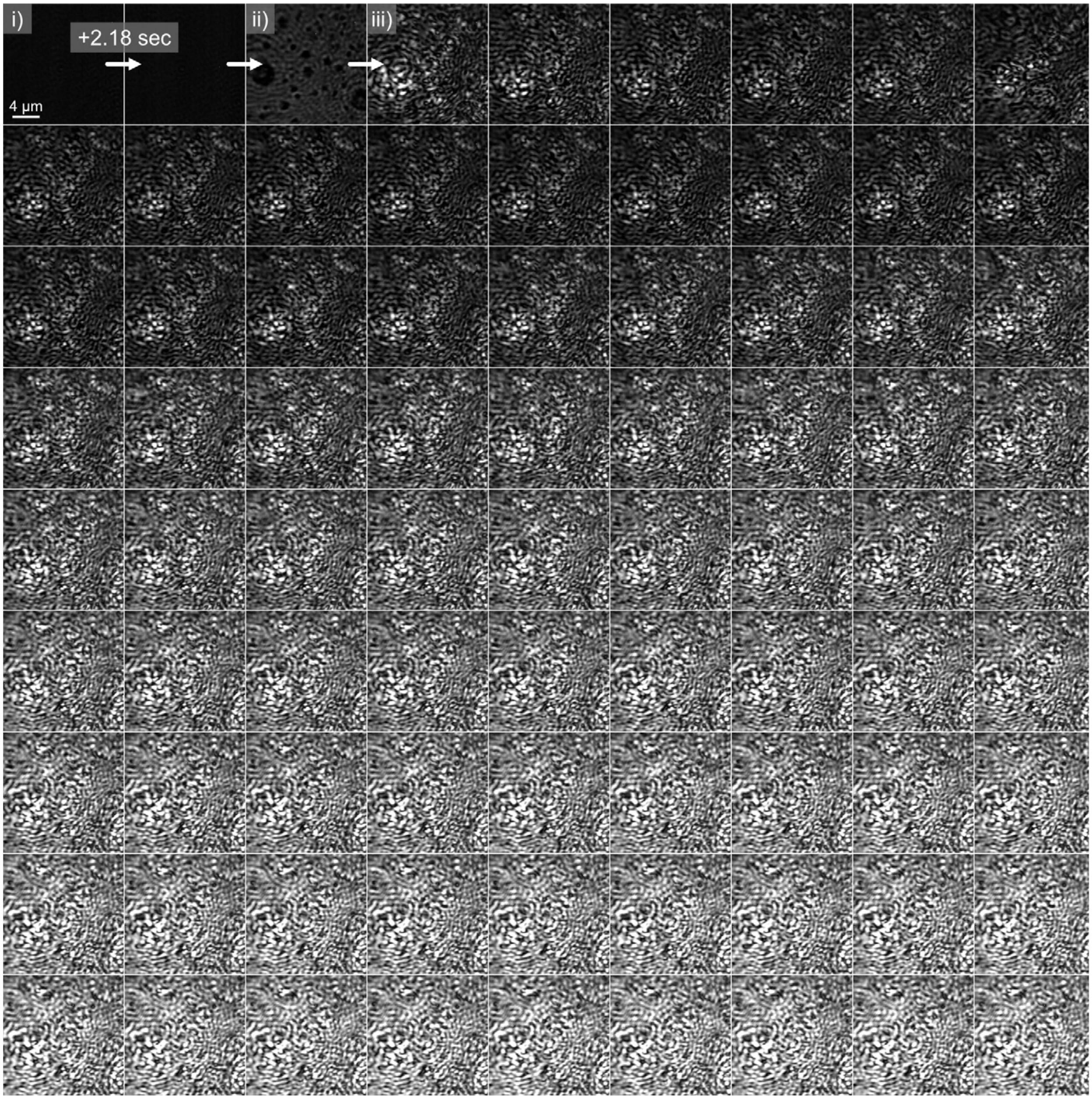

Supplementary Figure 3. Background-subtracted iSCAT images during TA-TAPB COF formation with 6M HOAc (time period: 177 sec). To the initial reactant solution (i ; TA and TAPB in 1,4-dioxane/mesitylene v/v 9:1), the aqueous catalyst mixture (1 equiv.) is added and the nucleation of black contrast mesitylene droplets are imaged (ii). This is followed by nucleation and growth of the COF framework (iii). Images were acquired at a speed of 21.8 ms per frame (46 fps), background-subtracted and 2x2 binned. To enhance visibility, a subset of images was selected from the 8100 acquired images. Specifically, every 100th image was chosen for display, resulting in a time difference of 2.18 seconds between the displayed frames. The contrast is adjusted to 0.84 – 3.11. Scale bar (applies to all images), 4 µm.

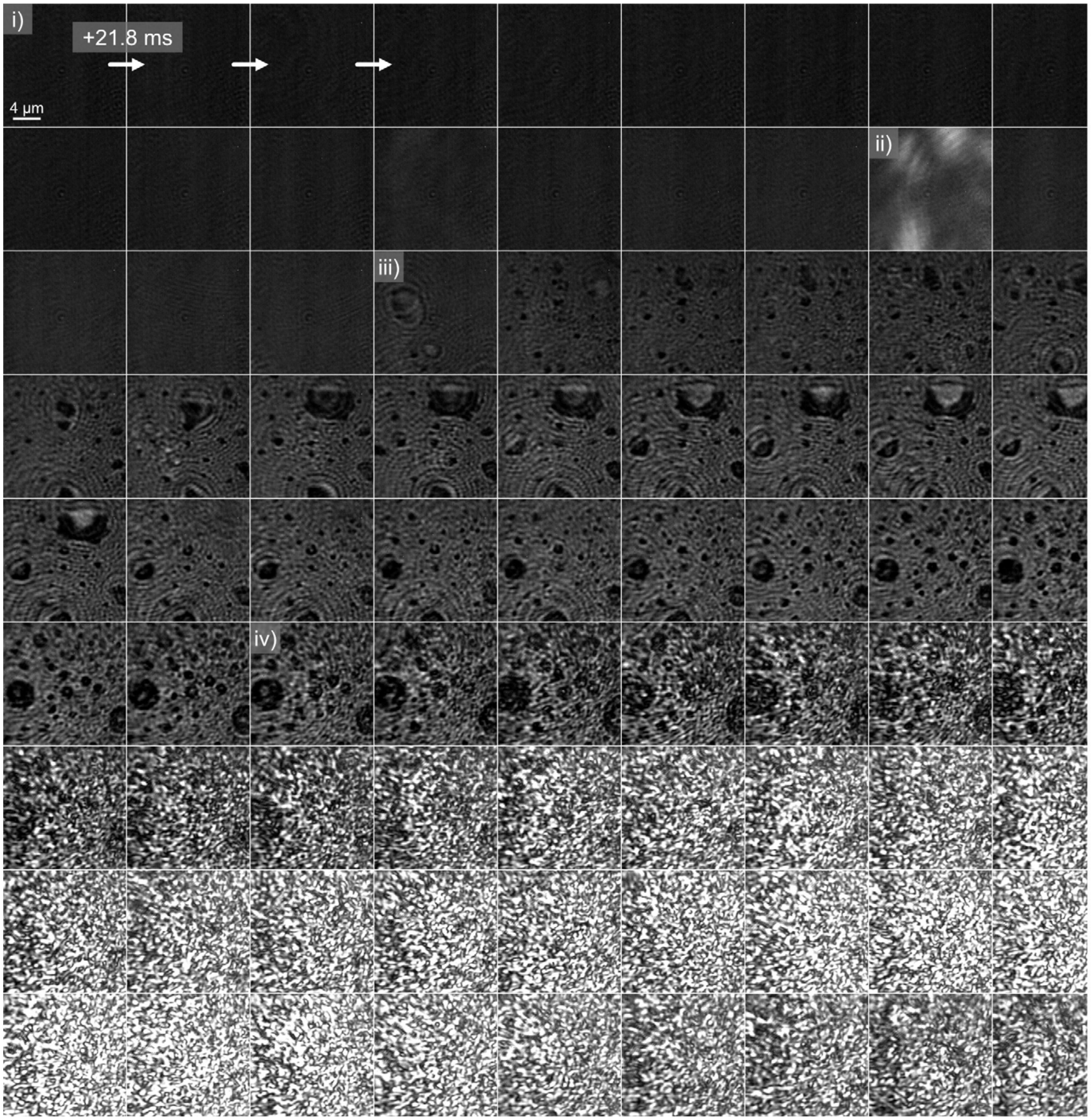

Supplementary Figure 4. Background-subtracted iSCAT images during the initial stages of TA-TAPB COF formation with 6M HOAc (time period: 1.77 sec). To the initial reactant solution (i ; TA and TAPB in 1,4-dioxane/mesitylene v/v 9:1), the aqueous catalyst mixture (1 equiv.) is added (ii). Afterwards, phase rearrangement processes and nucleation of black contrast mesitylene droplets are imaged (iii). In the following, this process is directly transitioning into the reaction and simultaneous nucleation on the surface (iv). Images were acquired at a speed of 21.8 ms per frame (46 fps), background-subtracted and 2x2 binned. The contrast is adjusted to 0.87 – 2.06. Scale bar (applies to all images), 4 µm.

3M HOAc:

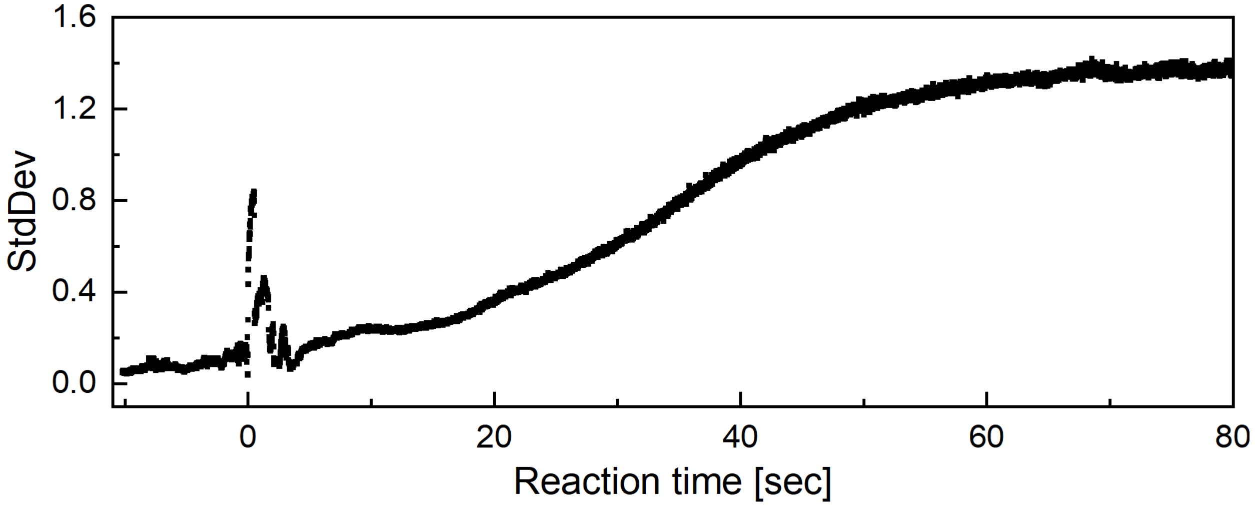

Supplementary Figure 5. Temporal evolution of the StdDev of the iSCAT images acquired during the formation of TA-TAPB COF with 3M HOAc. Images were acquired at a speed of 2.7 ms per frame (370 fps), background-subtracted, 2x2 binned and temporally averaged (5 frames).

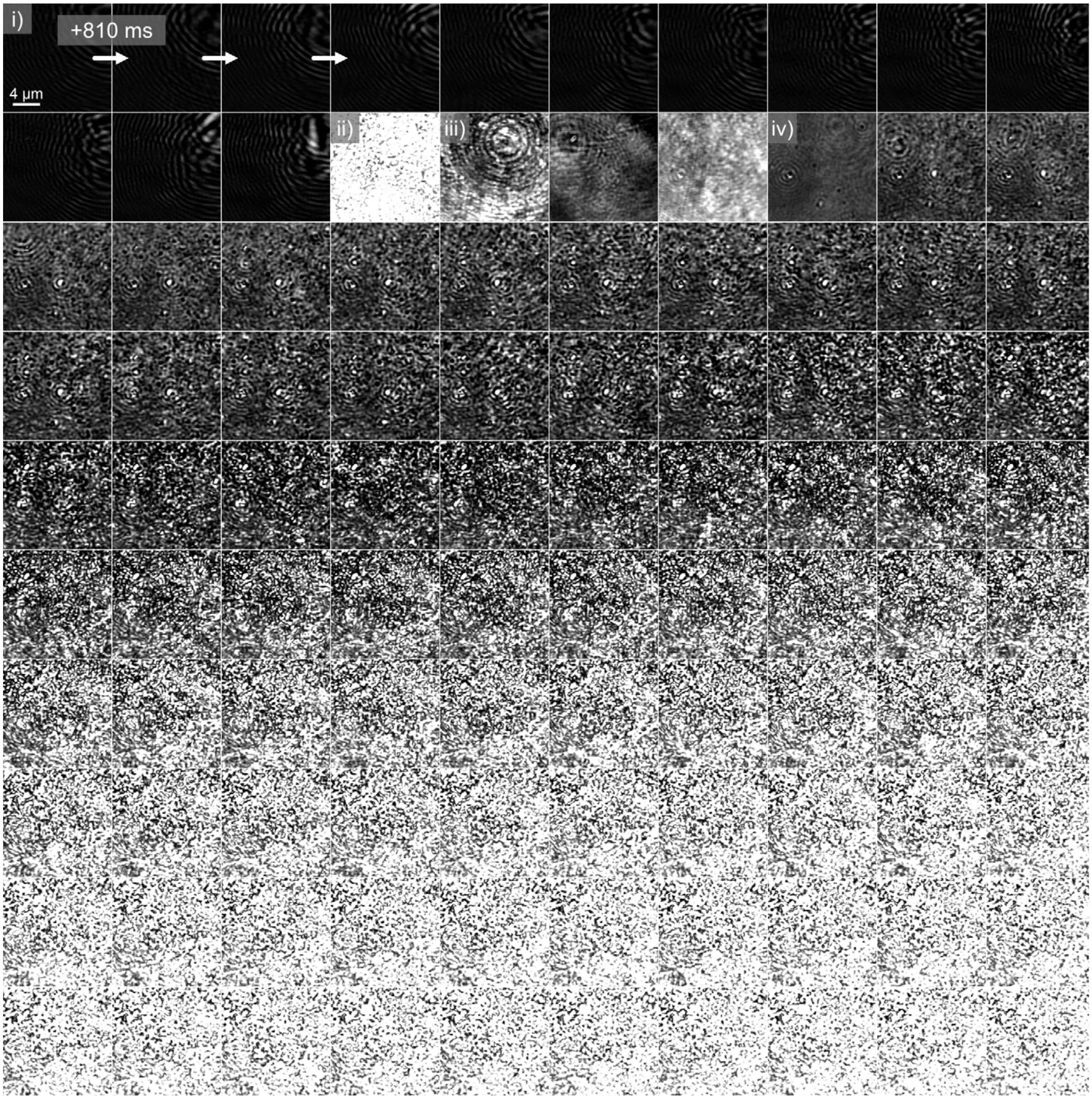

Supplementary Figure 6. Background-subtracted iSCAT images during TA-TAPB COF formation with 3M HOAc (time period: 81 sec). To the initial reactant solution (i ; TA and TAPB in 1,4-dioxane/mesitylene v/v 9:1), the aqueous catalyst mixture (1 equiv.) is added (ii). Subsequently, phase rearrangement processes and nucleation of black contrast mesitylene droplets (iii) are imaged. After dissolution of the mesitylene droplets, first solid matter is detected as black contrast spots on the surface (iv). For an extensive description of the displayed images, see the main manuscript. Images were acquired at a speed of 2.7 ms per frame (370 fps), background-subtracted and 2x2 binned. To enhance visibility, a subset of images was selected from the 30.000 acquired images. Specifically, every 300th image was chosen for display, resulting in a time difference of 810 ms between the displayed frames. The contrast is adjusted to 0.89 - 2.56. Scale bar (applies to all images), 4 µm.

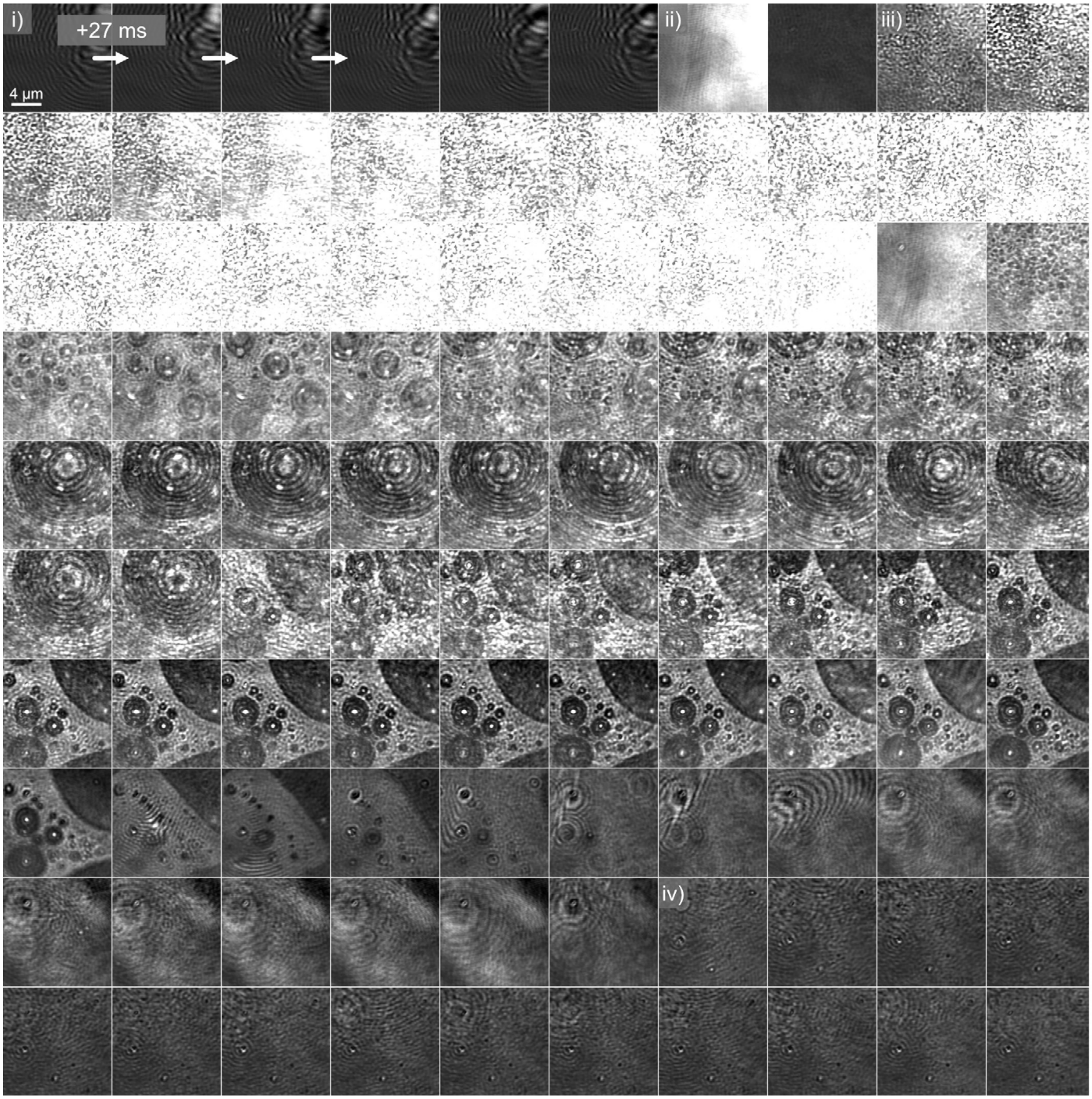

Supplementary Figure 7. Background-subtracted iSCAT images during the initial stages of TA-TAPB COF formation with 3M HOAc (time period: 2.7 sec). To the initial reactant solution (i ; TA and TAPB in 1,4-dioxane/mesitylene v/v 9:1), the aqueous catalyst mixture (1 equiv.) is added (ii). Subsequently, phase rearrangement processes and nucleation of black contrast mesitylene droplets (iii) are imaged. After dissolution of the mesitylene droplets, first solid matter is detected as black contrast spots on the surface (iv). For an extensive description of the displayed images, see the main manuscript. Images were acquired at a speed of 2.7 ms per frame (370 fps), background-subtracted and 2x2 binned. To enhance visibility, every 10th image acquired was chosen for display, resulting in a time difference of 27 ms between the displayed frames. The contrast is adjusted to 0.55 – 2.97. Scale bar (applies to all images), 4 µm.

1M HOAc:

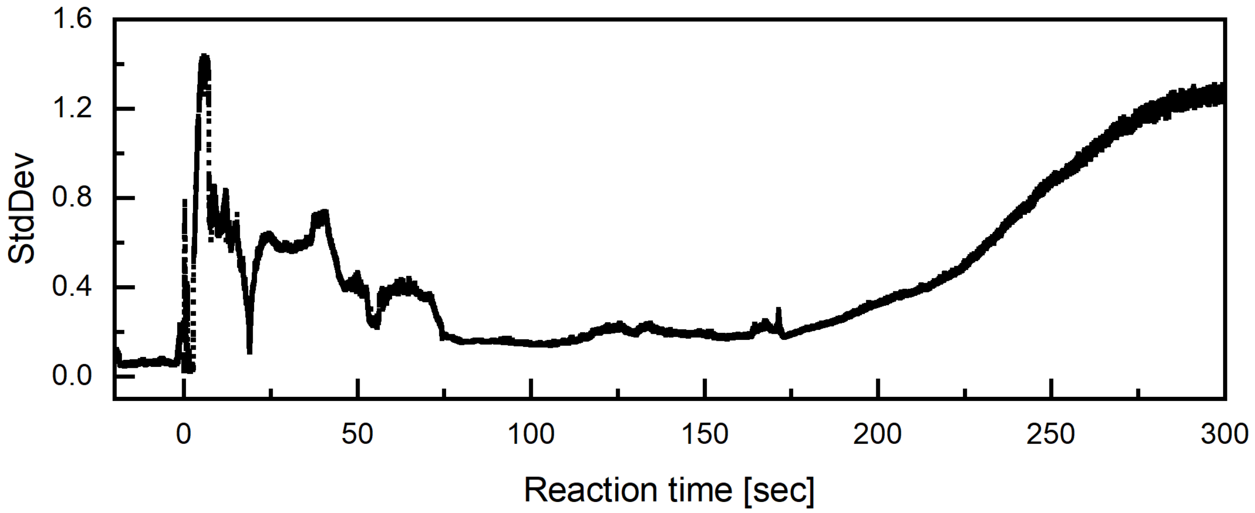

Supplementary Figure 8. Temporal evolution of the StdDev of the iSCAT images acquired during the formation of TA-TAPB COF with 1M HOAc. Images were acquired at a speed of 6.7 ms per frame (149 fps), background-subtracted and 2x2 binned.

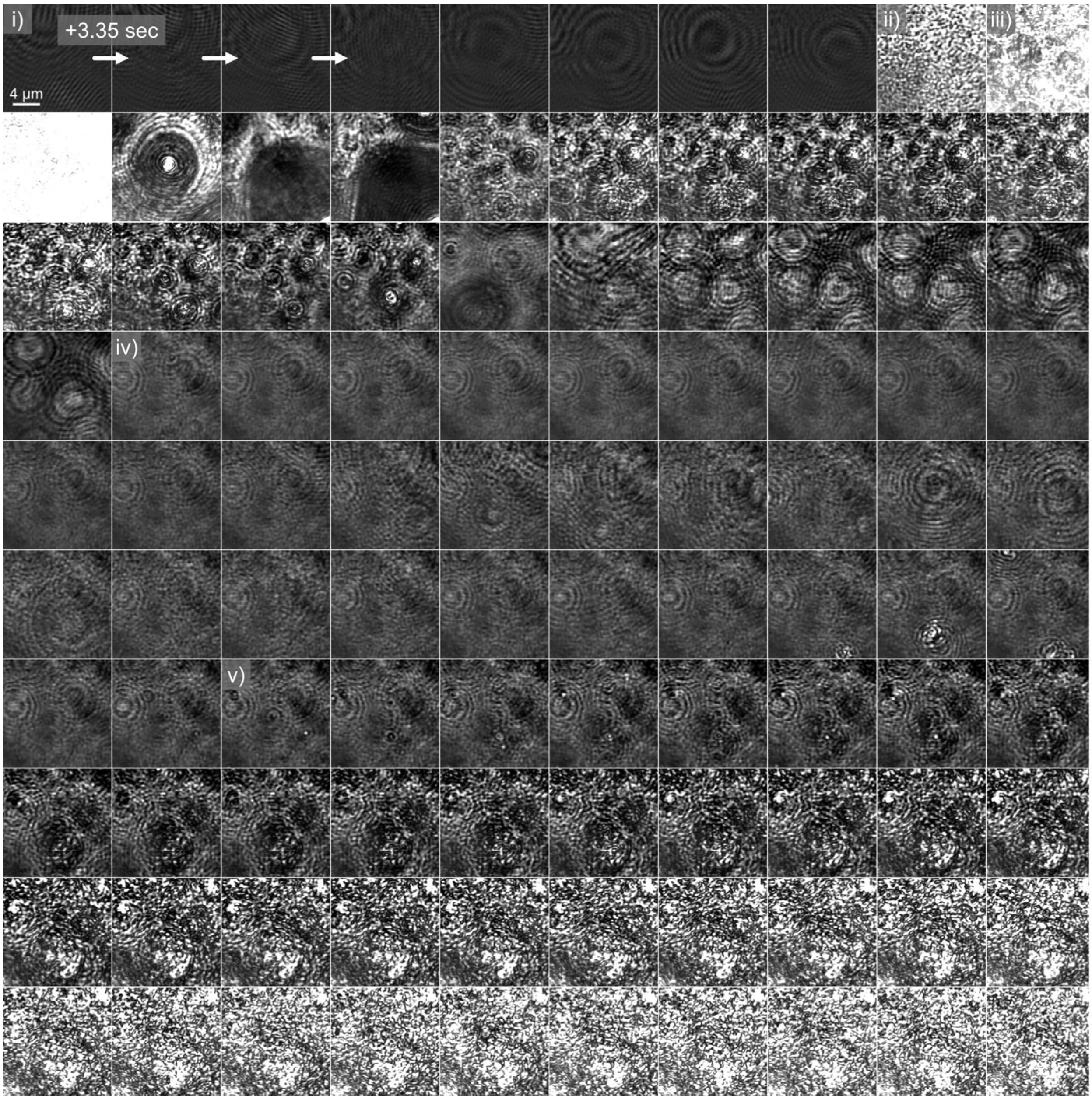

Supplementary Figure 9. Background-subtracted iSCAT images during TA-TAPB COF formation with 1M HOAc (time period: 335 sec). To the initial reactant solution (i ; TA and TAPB in 1,4-dioxane/mesitylene v/v 9:1), the aqueous catalyst mixture (1 equiv.) is added (ii). Subsequently, phase rearrangement processes and nucleation of black contrast mesitylene droplets (iii) are imaged. After dissolution of the mesitylene droplets (iv) first solid particles are detected on the surface (v) and subsequent nucleation and growth of the COF framework on surface starts. Images were acquired at a speed of 6.7 ms per frame (149 fps), background-subtracted and 2x2 binned. To enhance visibility, a subset of images was selected from the 50.000 acquired images. Specifically, every 500th image was chosen for display, resulting in a time difference of 3.35 sec between the displayed frames. The contrast is adjusted to 0.55 – 3.03. Scale bar (applies to all images), 4 µm.

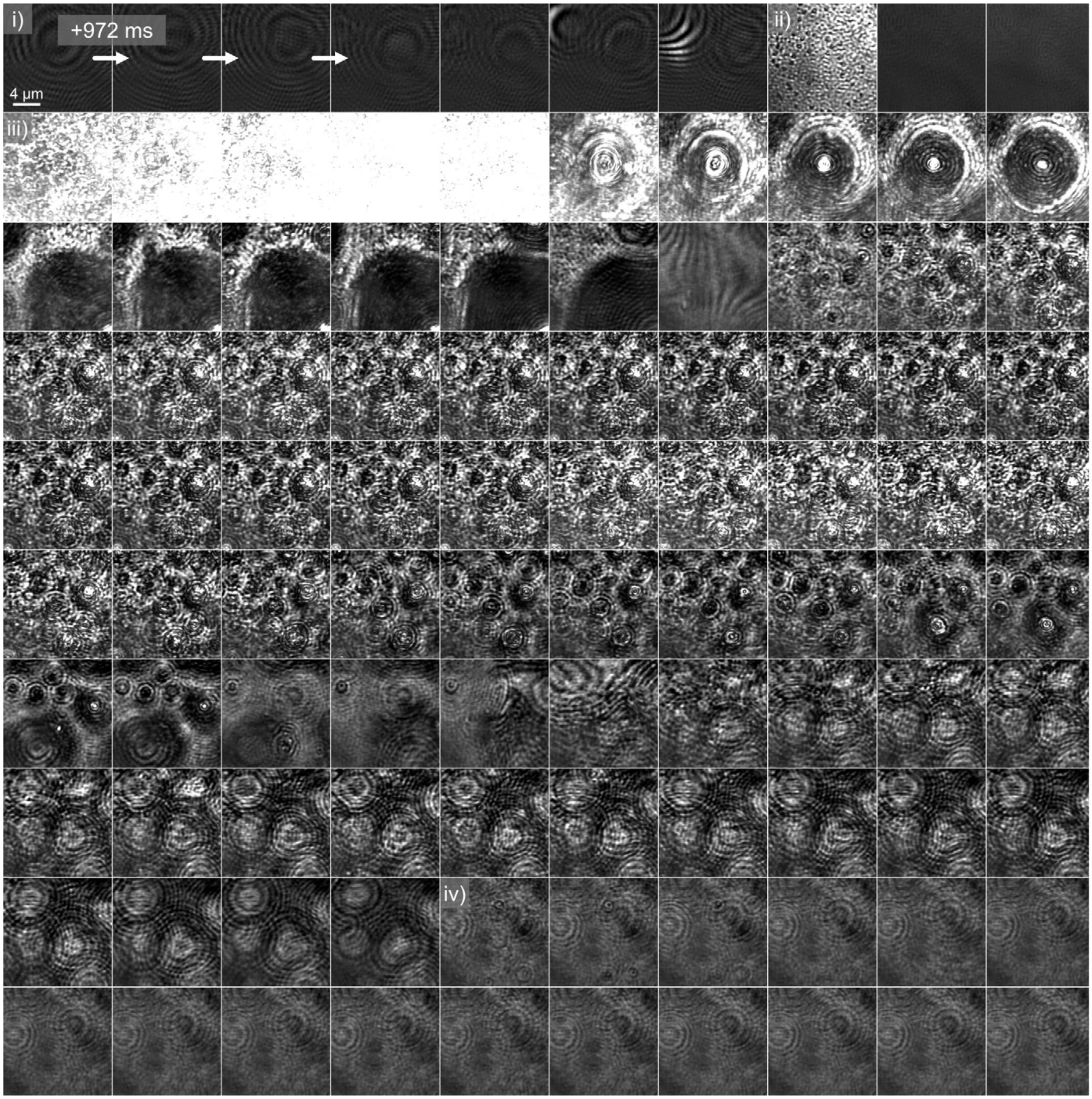

Supplementary Figure 10. Background-subtracted iSCAT images during the initial stages of TA-TAPB COF formation with 1M HOAc (time period: 97.2 sec). To the initial reactant solution (i; TA and TAPB in 1,4-dioxane/mesitylene v/v 9:1), the aqueous catalyst mixture (1 equiv.) is added (ii). Subsequently, phase rearrangement processes and nucleation of black contrast mesitylene droplets are imaged (iii), which then are dissolved into the solution (iv). Images were acquired at a speed of 6.7 ms per frame (149 fps), background-subtracted and 2x2 binned. To enhance visibility, every 145th image acquired was chosen for display, resulting in a time difference of 971.5 ms between the displayed frames. The contrast is adjusted to 0.60 – 2.96. Scale bar (applies to all images), 4 µm.

c) Confirmation of imine bond and COF formation under iSCAT conditions

To validate the chosen conditions (RT, 3M) for gaining insight into the initial stages of formation, we conducted additional analyses using infrared (IR) spectroscopy, X-ray diffraction (XRD), and scanning electron microscopy (SEM) (Supplementary Figure 11 - Supplementary Figure 14). Through these analyses, we confirmed that under the selected conditions within our iSCAT sample holder, imine bonds are formed, and crystalline covalent organic frameworks (COFs) with the anticipated spherical morphology are obtained after three days. This verification ensures that we are indeed imaging the relevant polymerization processes and suggests that the obtained results can be extrapolated to bulk syntheses conducted at higher catalyst concentrations (6M) and elevated temperature (120 °C). This extrapolation will be further supported and substantiated throughout the course of this study.

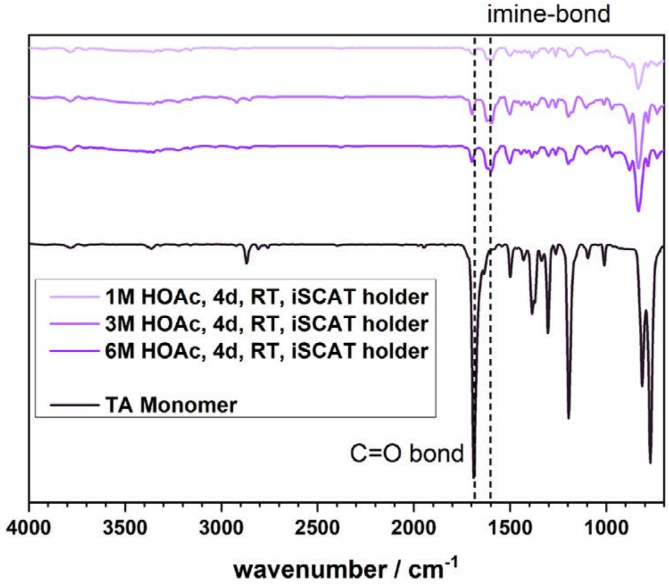

Supplementary Figure 11. FT-IR spectra of TA-TAPB COF synthesized in the iSCAT cell (all: 1,4-dioxane/mesitylene/aqueous catalyst, v/v 9:1:1; 4d, RT) with different concentrations of acetic acid as well as the TA-Monomer as reference. For TA, an absorption band at ≈1689 cm^{-1} corresponding to the carbonyl group stretching was observed. In contrast, this band was not detected in the spectra of the respective frameworks. Instead, the formation of an additional bond at around 1596 cm^{-1} was observed, indicating the successful formation of the respective imine bonds.[1]

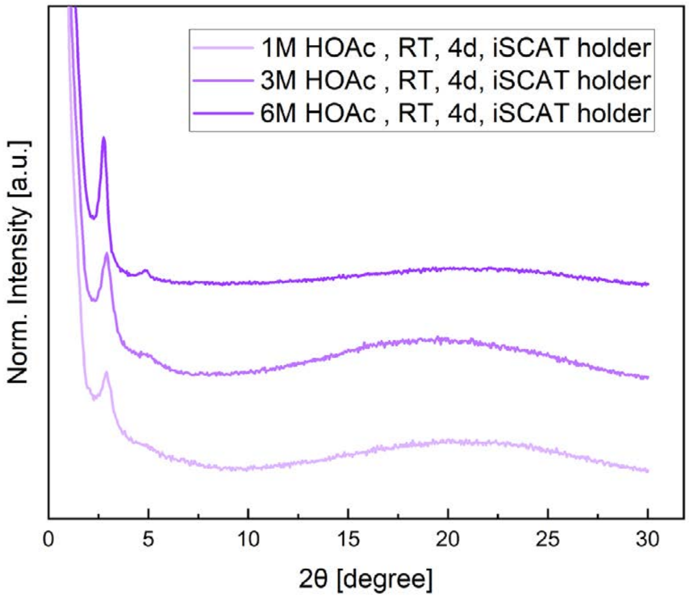

Supplementary Figure 12. Normalized PXRD patterns of TA-TAPB COF synthesized in the iSCAT cell at RT with different HOAc concentrations (all: 1,4-dioxane/mesitylene/aqueous catalyst, v/v 9:1:1; 4 d). Crystalline COF powder is obtained for all catalyst concentrations.

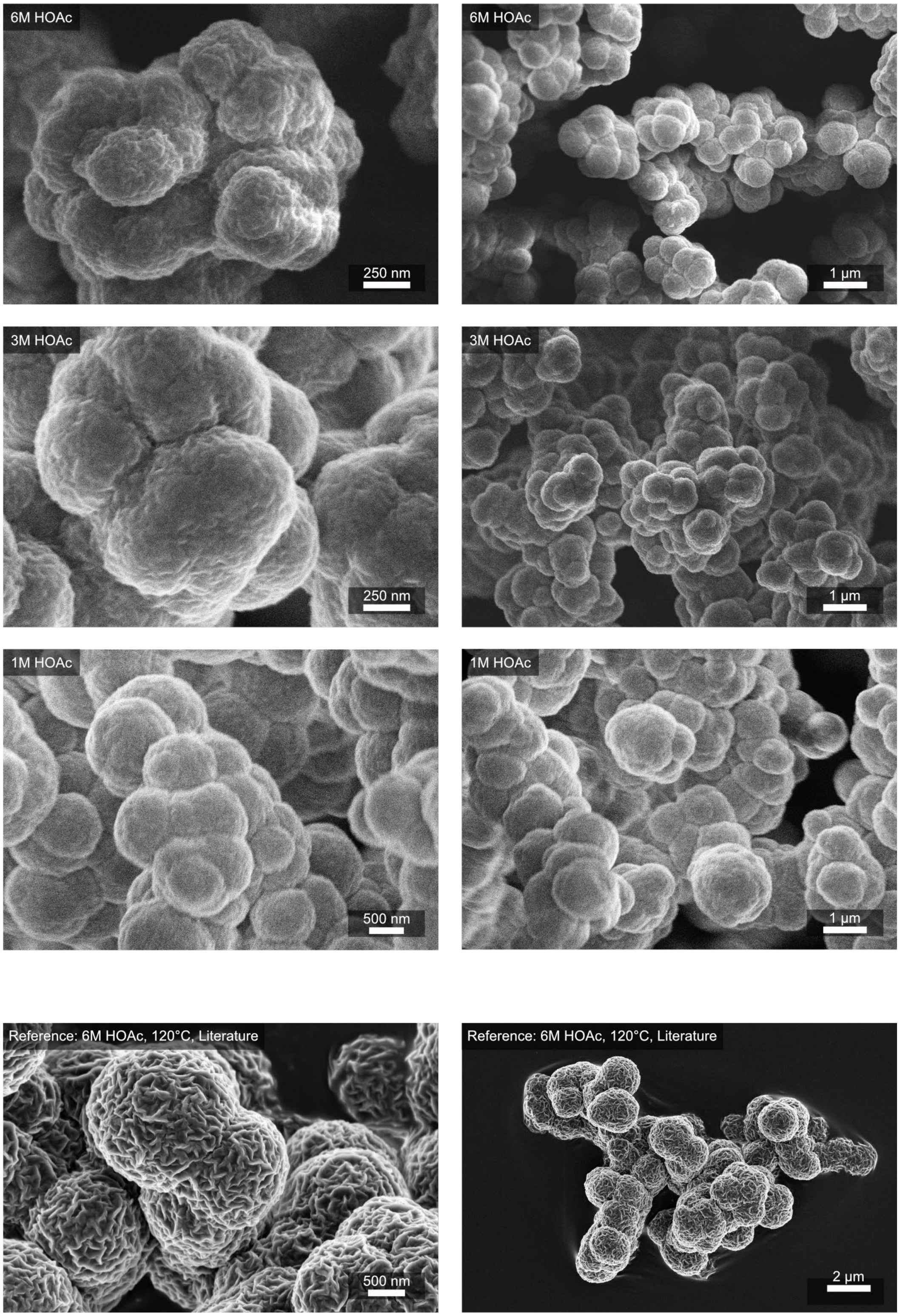

Supplementary Figure 13. Top, SEM of TA-TAPB COF synthesized in iSCAT sample holder with different HOAc concentrations (all: 1,4-dioxane/mesitylene/aqueous catalyst, v/v 9:1:1; RT, 4 d); bottom, Reference SEM of TA-TAPB COF synthesized in a Pyrex tube and literature conditions (1,4-dioxane/mesitylene/6M HOAc, v/v 9:1:1; 120 °C, 3 d). The spherical like morphology is maintained in all conditions.

d) Optical absorption

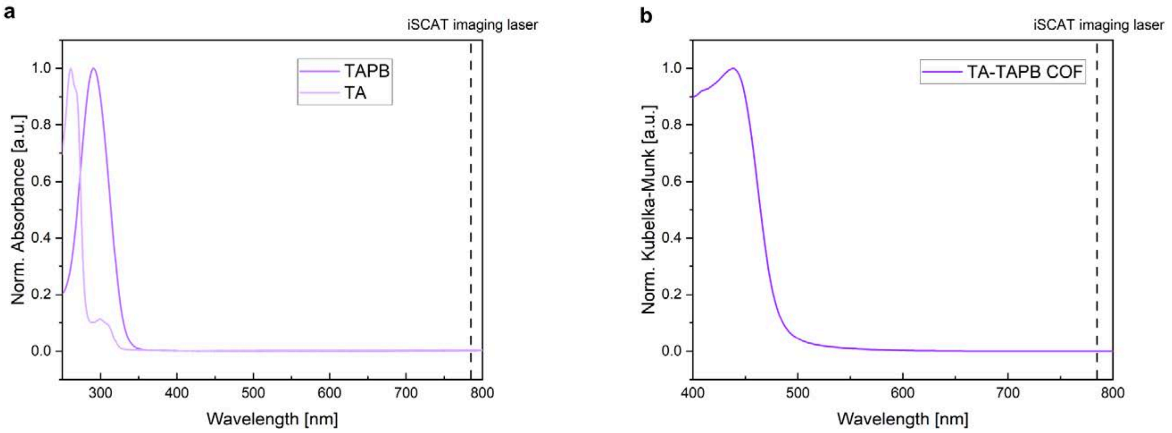

Supplementary Figure 14. **a** Normalized UV-Vis spectra of TA, TAPB in 1,4-dioxane. **b** Normalized Kubelka-Munk of TA-TAPB COF. Monomers and COF show no interaction with light via optical absorption at the iSCAT imaging laser wavelength of 785 nm.

Section 3. iSCAT methodology

a) iSCAT signal origin and contributions

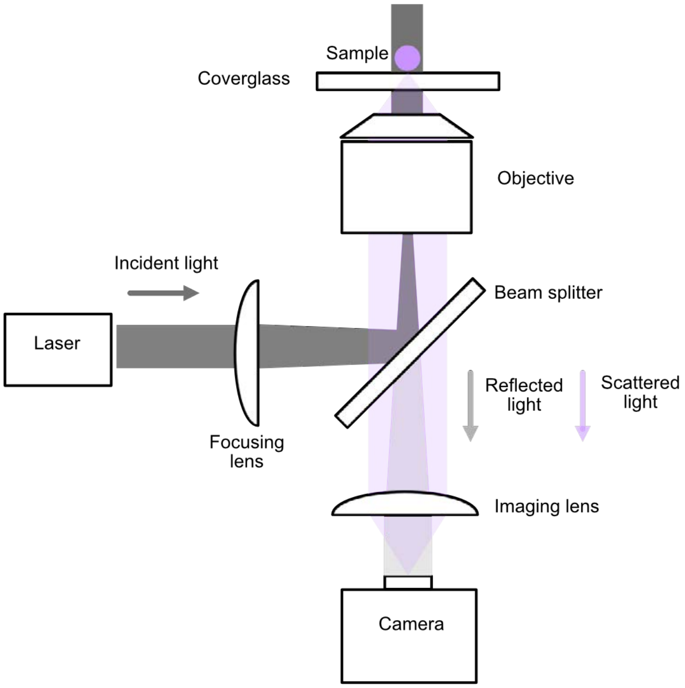

Supplementary Figure 15. Schematic overview of the beam path of the custom-built iSCAT microscope used in this study.

Supplementary Figure 15 displays the beam path of the used interferometric scattering microscope. The detected light at the camera of the microscope ($I_{detected}$) originates from the reflected light field ($E_{reflected}$) and the scattered light field ($E_{scattered}$), described by Equation 1.1 and 1.2.

$$I_{detected} = \left|E_{reflected} + E_{scattered}\right|^2 = \quad \text{(Equation 1.1)}$$

$$= \left|E_{reflected}\right|^2 + |E_{scattered}|^2 + 2\left|E_{reflected}\right|\left|E_{scattered}\right| \cos(\Delta\phi) \quad \text{(Equation 1.2)}$$

The reflected light emerges due to the refractive index difference at the interface between coverglass and reaction medium. It does not contain sample information, is static and can be removed by background subtraction leaving only the scattering features from the sample itself (see for more details Section 1b)). The sample information is contained in the scattered light as it originates from local refractive index changes during the reaction, e.g., the emergence and growth of polymer nucleation seeds. The scattered light field is proportional to the complex polarizability α of the scattering matter:[2]

$$E_{scattered} = \alpha E_{incident} \quad \text{(Equation 2)}$$

with α (for a deeply sub-wavelength sphere in the quasistatic approximation):

$$\alpha = 3\epsilon_m V \left(\frac{\epsilon_s - \epsilon_m}{\epsilon_s + 2\epsilon_m}\right) \quad \text{(Equation 3)}$$

where V is the particle volume, ϵ_s and ϵ_m the permittivities of the scatterer and the surrounding medium, respectively. The value of α is primarily dictated[2] by the volume of the scatterer, V, which is in turn proportional to the third-power of the diameter of the scatterer, d^3.

The light scattering materializes in two contributions in the signal at the camera (see Equation 1.2) - a pure scattering term ($|E_{scattered}|^2$, exploited in dark-field microscopy) and an interferometric scattering term ($2|E_{reflected}||E_{scattered}|\cos(\Delta\phi)$). In case of small scatterers (ca. d < 50nm), the pure scattering term can be neglected as it has a sixth-power dependence on the diameter of the scatterer (dividing the diameter in half results in 64 times less signal) compared to a third-power dependence for the interferometric scattering. Therefore, the imaged iSCAT intensity for small scatterers originates from the interference term ($2|E_{reflected}||E_{scattered}|\cos(\Delta\phi)$) in Equation 1.2., where the interference is between the scattered light and the reflected light. Here, the reflected light acts as reference for the constructive or destructive modulation. The interferometric scattering signal emerges a small variation on top of the large background of sample-independent reflections and is highlighted only after image processing. The third-power relationship on the diameter (compared to a sixth-power relationship for pure scattering) makes it possible to detect very weak scatterers.

However, for larger scatterers, the pure scattering term cannot be neglected. As the diameter increases, the pure scattering contribution begins to rival the interference term and eventually surpasses it, thanks to its sixth-power dependence. Consequently, the pure scattering contribution becomes the primary factor in the detected signal. This can be experimentally visualized, e.g., during the growth of a nucleation seed (Extended Fig. 2). Here, the signal contrast transitions from negative/black (interferometric scattering) to positive/white (pure scattering) due to the different signs of the two contributions in Equation 1.2. As a general remark, depending on the focus position, the interferometric signal of small scatterers can also exhibit a positive, white contrast due to the Gouy phase shift.[3]

b) Background correction and image processing

Background correction plays a crucial role in iSCAT microscopy by eliminating static background features present in the raw images captured by the camera. These background features originate from reflected light from the coverglass, impurities on the optical components, and spurious back-reflections, among others.[4] For background correction, we adopt the temporal median approach. Here, we calculate the median pixel intensity for each pixel across the first 300 raw images, which were captured at successive time points before the catalyst was added. This process generates a median image that represents the static background features observed in the measurement. Subsequently, each raw image within the acquired image stack is divided by this median image, effectively eliminating the static background features from the images. The resulting images only

contain the dynamic changes that occurred during the acquisition time (as depicted in Supplementary Figure 16).

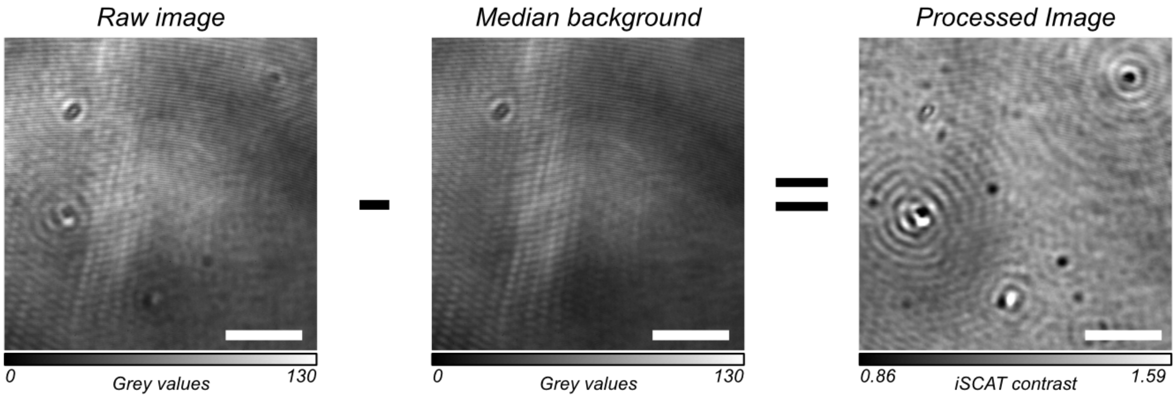

Supplementary Figure 16. Background subtraction in iSCAT image processing. Dividing the raw images by the median image of the first 300 frames eliminates all static background features that mask the dynamic reaction processes (here, the attachment of nucleation seeds). The processed image is additionally 2x2 binned and temporally averaged (5 consecutive frames). Scale bar (applies to all images), 4 µm.

A remaining contribution to the background noise which is inherent to the measurement principle is shot noise. It arises from statistical fluctuations in the number of photoelectrons (N) detected at the camera.[5] The shot noise is proportional to $\sqrt{N}$ and can therefore be reduced by increasing the incident laser illumination and hence the measured photoelectrons. However, experimentally one is limited by the number of photoelectrons the camera can detect (full well depth). Another way to mitigate this noise is through image processing techniques. In this work, two image processing approaches have been utilized: pixel binning and temporal averaging of images.

Pixel binning involves averaging neighboring pixels and electronically combining them, effectively increasing the number of detected photoelectrons per pixel. In this work, all displayed images were 2x2 pixel binned if not explicitly mentioned otherwise, which means that a square of four adjacent pixels was averaged into one pixel (raw image of 512x512 pixels to processed image of 256x256 pixels). This increases the effective number of detected photoelectrons by a factor of four and reduces the shot noise by a factor of two ($\sqrt{4 * N}$).

Temporal averaging of frames follows a similar principle, but instead of averaging pixels within the same image, pixels across successive images are averaged. In this work, where explicitly stated, five successive images are averaged into one image. Each pixel in the averaged image represents the average of five pixels taken at different times but at the same location. This reduces the shot noise by a factor of the square root of five ($\sqrt{5}$). However, it should be noted that this averaging process also sacrifices time resolution.

c) Acronyms for microscopes exploiting interferometric detection

There are several acronyms for microscopes exploiting the interferometric detection principle used in this work, two prominent cases being interference reflection microscopy (IRM) and interferometric scattering microscopy (iSCAT).[2,6]
While the mechanical setups are comparable, a setup is commonly referred to as IRM when the scatterers imaged are entities that are micrometer-sized or larger (diameter >> incident wavelength) and therefore the concept of reflection is better suited for describing the signal origin, while it is typically referred to as iSCAT in case of scatterers which are sub-wavelength in size (diameter ≤ incident wavelength). In COF formation processes, the imaging includes sub-wavelength particles (nucleation-seeds) as well as micrometer-sized entities (liquid droplets). We refer to our microscope as iSCAT, as umbrella term also covering the imaging of small scatterers. Furthermore, most of the methodology we refer to is from the field of iSCAT microscopy.

d) StdDev as figure of merit for temporal iSCAT traces

To comprehensively analyze the temporal evolution of the changes in the scattering properties of the probed region induced by reaction processes,[7] we calculate the standard deviation (StdDev) of all pixel intensity values for each processed iSCAT image. These StdDev values are then plotted against time.
An increase in StdDev represents a broadening of the signal intensity distribution compared to the initial situation of the reaction solution before catalyst addition. Here, the average is located at 1.0 with a small spread in the intensity distribution (ergo, StdDev) which mainly originates from shot noise. Phrased differently, an increase in StdDev results from an increase in scattering volume that has a different local refractive index compared to this initial situation of the reaction medium before catalyst addition. Consequently, the StdDev is a viable parameter to assess the reaction stages and their progress.

e) Differentiation between scatterers floating in solution and attached on the substrate

We distinguish between something floating in solution and attached on the surface of the coverglass based on two factors. First, entities floating in solution change their x-y position while something attached on the coverglass is immobilized with a constant x-y position in our case. Second, the z-position of the nanoscale particle changes/fluctuates when floating in solution while it is fixed when attached on the coverglass. This has implications on the observed signal, as the interferometric point spread function (iPSF) depends on the particle position relative to the coverslip and the focal plane.[8] The iPSF is composed of a central peak (exploited for determining sample size) and rings of particular contrast and diameter – both of which are dependent on the axial position of the particle with respect to the focus of the objective.[9] While the on-surface particles are characterized by high contrast of their central peak (in our case, black contrast dots in image; main Fig 1c, iii), for the strongly out-of-focus floating entities only rings are imaged (main Fig. 1c, i).

f) Size estimation based on iSCAT contrast

Most of the initial precipitates breaking out of solution and attaching on the coverglass exhibit an iSCAT contrast of around 35% (see Fig 2b v, vi; see also Extended Figure 4).

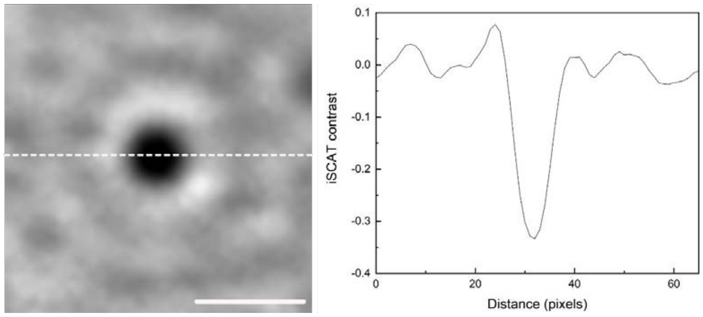

Supplementary Figure 17. Cross section of an exemplary particle detected in the initial stages. Scale bar, 1 μm.

The iSCAT contrast is proportional to the scattered light field which in turn is proportional to the complex polarizability α of the scatterer. Therefore, the contrast is determined by the volume and the refractive index of the scattering matter and its surrounding medium (see Section 3a). For polystyrene spheres (n_{PS}= ca. 1.62[8]) in water (n= 1.33), which is a similar system to ours (n_{COF} = ca. 1.59[10]; $n_{diox/mes/water}$ = 1.41 - SI section 8), 35% contrast corresponds to a particle diameter of 85 nm. In the COF system, the refractive index of the medium is slightly closer to that of the particle, resulting in less scattered light compared to the polystyrene case. However, as the scattered light and therefore the contrast scales with the third power of the particle diameter it is fair to assume that the initial particles are in the range of $d \leq 100$ nm. In future studies, we envision that an extensive contrast to volume relation is established either by measuring colloidal COF particles[10] or by theoretical simulation.[8,11]

Section 4. COF crystallization mechanism in current literature

The formation of COFs towards crystalline and porous materials, is a complex process to trace. The conventional techniques to elucidate the processes are lacking the combination of a suitable time resolution, spatial resolution and sensitivity to the diverse nano-matter present in the mixture, especially at the early stages of molecular interactions and in operando.[12] Because of these limitations, there are still open questions and vibrant discussions around the topic of the exact crystallization mechanism behind the conventional COF synthesis under solvothermal conditions. To tackle the intricacies of these complex systems, even theoretical predictions try to assist with the understanding of the process by performing computational studies on the COF synthesis.[13,14] In the following, we bring forward a few prominent reports dealing with the experimental investigation of COF growth mechanisms; it is not a review of the literature but a compressive discussion.

Initially, it was almost a consensus for (imine) COFs that during the fast-initial polymerization stage (condensation reactions of the monomers) an amorphous polymer is produced followed by the emergence of long-range order through defect healing by dynamic covalent chemistry (DCC) over a time span of days.[15,16] However, recent reports by Dichtel, Evans, Medina et al. are pointing towards a more complex process for obtaining a crystalline COF product (e.g., TA-TAPB COF). Medina et al. and several other groups showed the product isolation procedures such as vacuum activation and solvent washing are critical for the long-range order of the COFs obtained. Indicating that the overall crystallinity of those structures is dependent on many factors and that relying on the final product only provides with part of the overall crystallization picture.[1,17,18] Following this, it has been reported by Dichtel et al., after exploiting gentler activation techniques, that the very initially precipitated species are, at least partially formed as a few-layer disorganized crystalline sheets. In situ synchrotron XRD measurements confirmed that crystalline matter can emerge more rapidly than previously anticipated and that at the earliest possible measurement time of 90 sec ordered material exists in the mixture (TA-TAPB COF).[18] In a later report,[19] Zhao et al. describe the crystallization of TA-TAPB COF process by nucleation and growth stages. At first, a crystalline COF phase can develop rapidly from self-templated monomers along with an amorphous phase which is later transferred via a self-healing growth stage to a crystalline phase. Thereby, the overall crystallinity increases mainly due to the conversion of the amorphous phase to a crystalline phase. This slower amorphous to crystalline transformation has been previously reported on several occasions.[12,18–20] It is considered that the process is facilitated by the DCC of the initial defective material into long-range ordered COFs (which are more stable towards the conventional vacuum activation).

In a review by Evans et al. the authors proposed that, “a combination of mechanisms is active in all dynamic 2D COF polymerization reactions, the extent of which depends greatly on the polymerization conditions and polymer system studied“.[12]

Section 5. Refractive index of binary and ternary solvent systems in the TA-TAPB COF reaction

While there are several popular mixing rules to calculate the refractive index of binary solvent systems (e.g., Gladstone-Dale, Lorentz-Lorentz, or Oster),[21] in the case of ternary solvent systems these mixing rules are not commonly applied. Nevertheless, it has been shown that they provide a good estimate of the refractive index of various ternary solvent systems (relative error in the magnitude of 1%).[22,23] Furthermore, it has been reported that the estimation of the refractive index via calculation is satisfactory for ternary solvent systems that form surfactant-free emulsion.[23]

Here, we use on of the most common mixing rules, the Gladstone-Dale relation, to estimate the refractive index of the binary and ternary solvent systems occurring in this work:[22]

$$n - 1 = \sum_{i=1}^{k} (n_i - 1) * \phi_i$$

With n is the refractive index of mixture; n_i is the refractive index of the pure component i; k is the number of the mixture components; ϕ_i is the volume fraction of the pure component i:

$$\phi_i = \frac{x_i V_i}{\sum_{i=1}^{k} x_i V_i}$$

An overview of the obtained refractive index values is given in the Supplementary Table 2:

**Solvent system**	**Refractive index (@785 nm)**
Water	1.330[24]
1,4-Dioxane	1.417[25]
Mesitylene	1.500[26]
1,4-Dioxane / Mesitylene (8:1)	1.426
1,4-Dioxane / Mesitylene / Water (8:1:1)	1.417
Coverglass (Schott D 263® M)	*1.517*[27]

Supplementary Table 3. Literature refractive index values for solvents and calculated refractive indices of binary and ternary solvent systems.

Section 6. Reflectivity of solvents and solvent mixtures in iSCAT

The reflectivity at the interface between the coverglass and the medium can be determined using the Fresnel equations, which take into account the refractive index and the angle of incidence. We assume normal incidence for our setup; thus, the reflectivity is given by:

$$R_0 = \left|\frac{n_1 - n_2}{n_1 + n_2}\right|^2$$

By utilizing the refractive indices from Section 5, we compute the reflectivity for various solvent compositions and then compare these results with the corresponding iSCAT measurements. The iSCAT images for each solvent mixture are obtained with an exposure time of 3 milliseconds, a resolution of 512 x 512 pixels. Subsequently, a Gaussian blur filter (100 pixels) is applied. The mean pixel intensity observed in the iSCAT images directly correlates with the calculated reflectivity at the "coverglass"-"medium" interface, as demonstrated in Supplementary Figure 18. The specific case of mesitylene/water (1:1) was selected to highlight the probing of the "coverglass"-"medium" interface reflectivity. Mesitylene and water have very low miscibility, resulting in two separate liquid phases. Due to its higher density (997 kg/m³ vs. 864 kg/m³), water forms the lower layer, while mesitylene floats on top. Consequently, the probed reflectivity essentially corresponds to that of water, with a minor reduction in intensity compared to pure water.

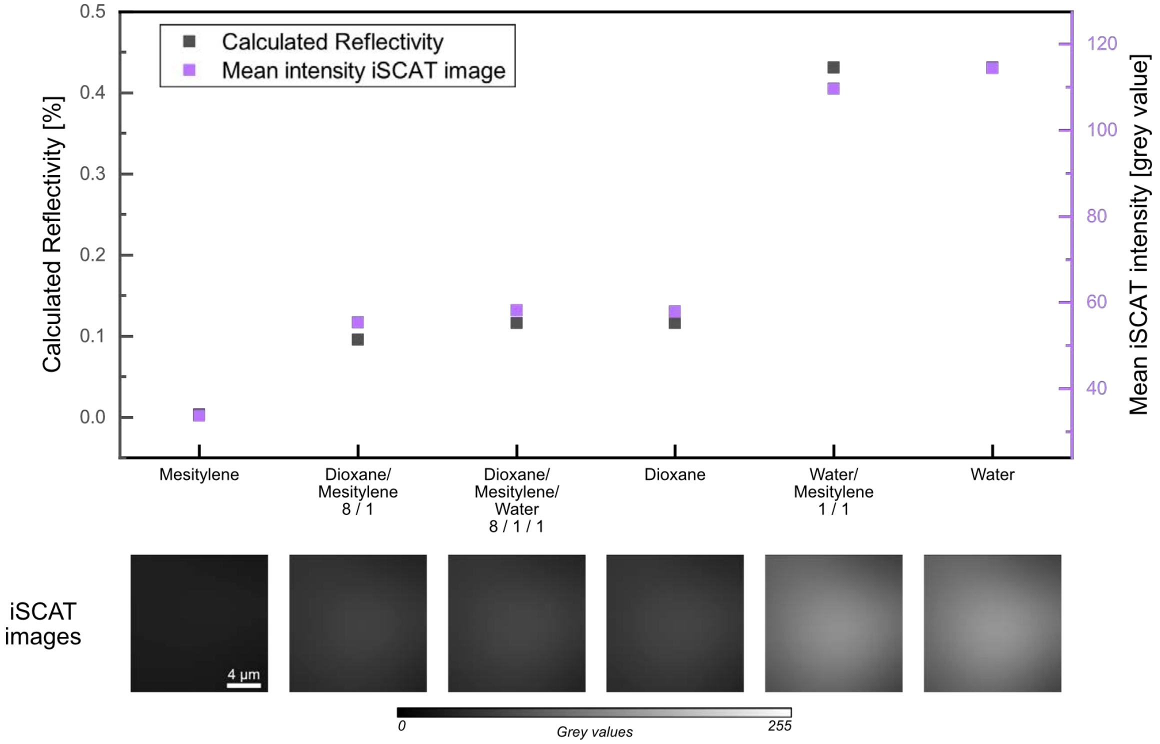

Supplementary Figure 18. Plot showing the calculated reflectivity and the mean iSCAT intensity for different solvents and solvent mixtures. iSCAT images are visualizing the different refractive indices of the solvents used in COF synthesis. Scale bar (applies to all images), 4 µm. In the particular case of water/mesitylene (v/v 1:1), the solvents have a low miscibility and the reflectivity imaged in iSCAT corresponds to the separated water layer that constitutes the "coverglass"-"medium" interface.

Section 7. Solvent restructuring processes visualized with iSCAT

Our objective was to unravel the underlying processes occurring during solvent restructuring by discerning the reaction and phase rearrangements. To achieve this, we conducted measurements using a solute monomer (TA) and water. A single reactant was included to prevent any undesired reaction, as even the addition of water, instead of a catalyst, can trigger a minor reaction resulting in subsequent nanoscale solid precipitates. Furthermore, to eliminate any chemical interaction between the monomer and the catalyst, we transitioned from using HOAc to water. To enhance the clarity of the dynamics, we employed a 4:1 1,4-dioxane/mesitylene mixture to which 1 equivalent of water was added (Supplementary Figure 19 - Supplementary Figure 21).

Additionally, to visualize the origin of the black droplets, we initiated experiments with a 2:1 1,4-dioxane/water mixture and gradually introduced mesitylene in 10 µl increments up to 100µl (Supplementary Figure 22). With increasing amount of mesitylene, the previous black non-equilibrium droplets only visible during the phase rearrangements become at some point stable, steady-state droplets that accumulate on the surface. The size of these droplets correlates with the amount of mesitylene introduced to the system.

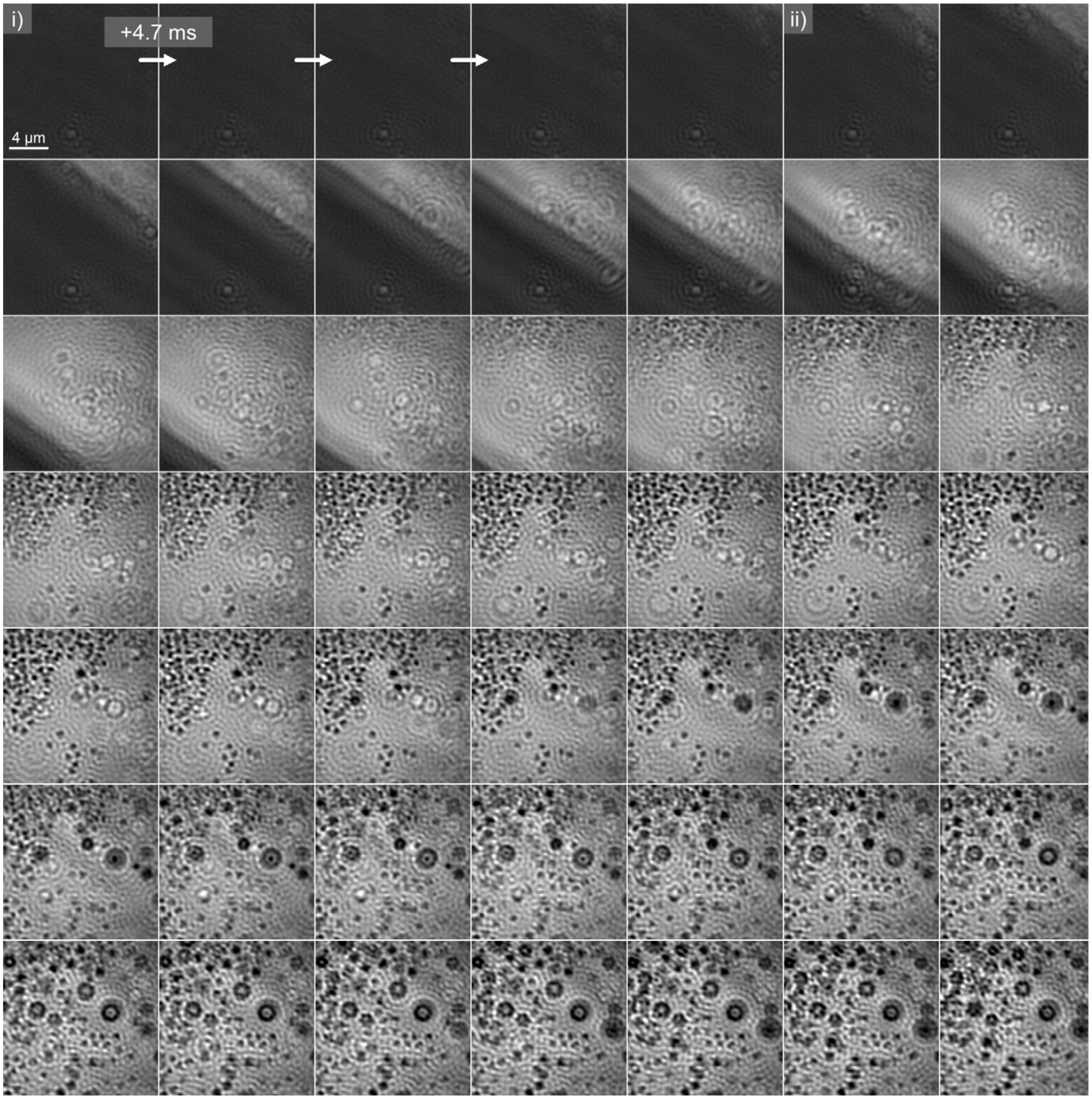

Supplementary Figure 19. Background-subtracted iSCAT images of the nucleation of liquid mesitylene droplets (time period: 230.3 ms). To a model system (i) of 1,4-dioxane/mesitylene (v/v 4:1; 100 µl) and TA (1.01 mg, 0.045 mmol) , water (1 equiv.) was added. After addition, a white front with high iSCAT signal propagates from the right upper corner of the image (ii), indicating the distribution of water on the coverglass surface. Concurrently, black spots and out-of-focus Point Spread Functions (PSFs) emerge within the white contrast medium and attach to the coverglass surface. This phenomenon corresponds to the nucleation of hydrophobic mesitylene, facilitated by the high polarity environment induced by the presence of water. Images were acquired at a speed of 4.7 ms per frame (212 fps), background-subtracted and 2x2 binned. The contrast is adjusted to 0.75 – 2.0. Scale bar (applies to all images), 4 µm.

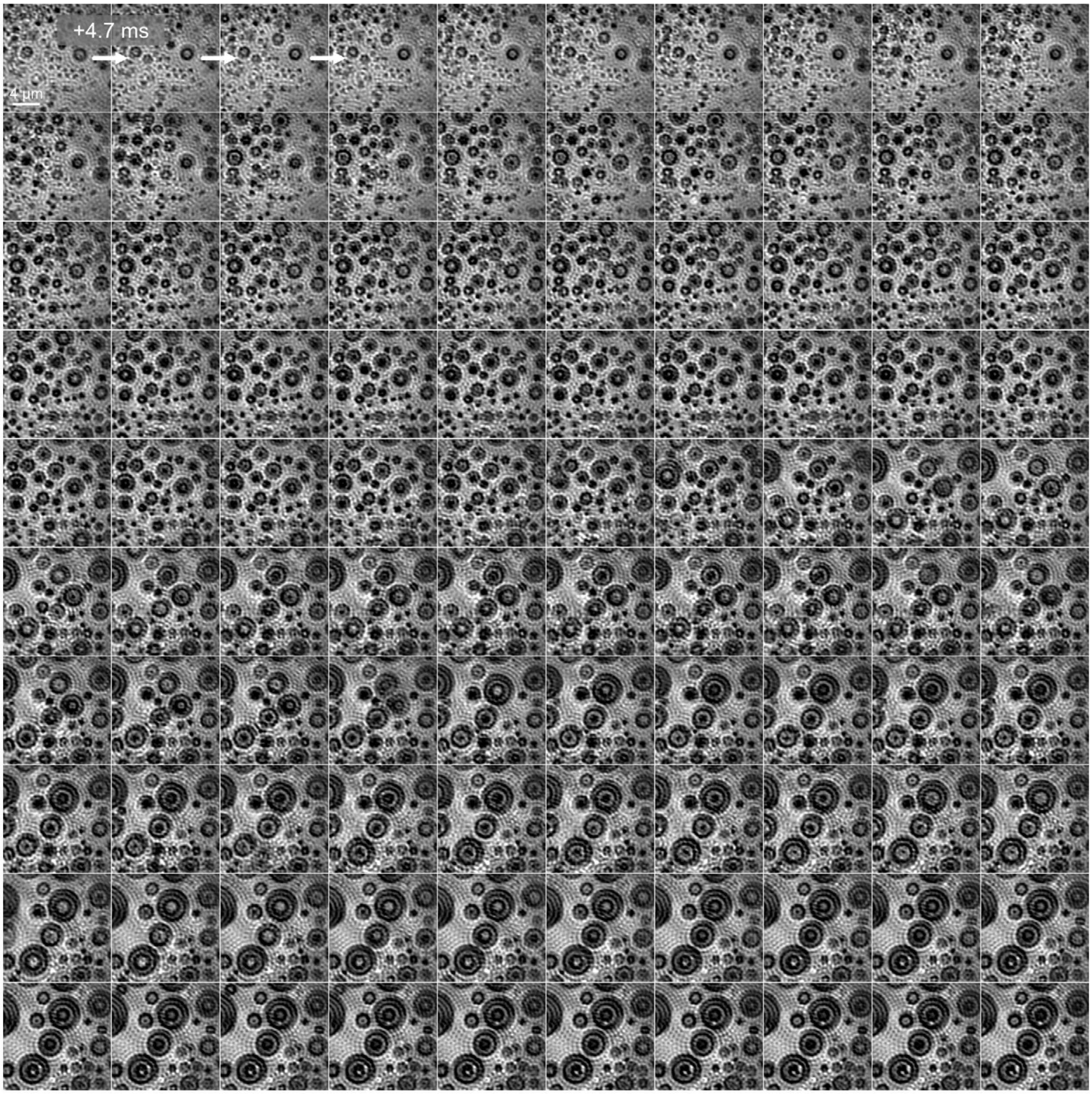

Supplementary Figure 20. Background-subtracted iSCAT images of the Ostwald ripening of liquid mesitylene droplets (time period: 470 ms). Here, water (1 equiv.) was added to a model solvent system of 1,4-dioxane/mesitylene (v/v 4:1; 100 µl) and TA (1.01 mg, 0.045 mmol), leading to the nucleation of dark contrast mesitylene droplets. The images show their growth via Ostwald ripening. Images were acquired at a speed of 4.7 ms per frame (212 fps), background-subtracted and 2x2 binned. The contrast is adjusted to 0.75 – 2.0. Scale bar (applies to all images), 4 µm.

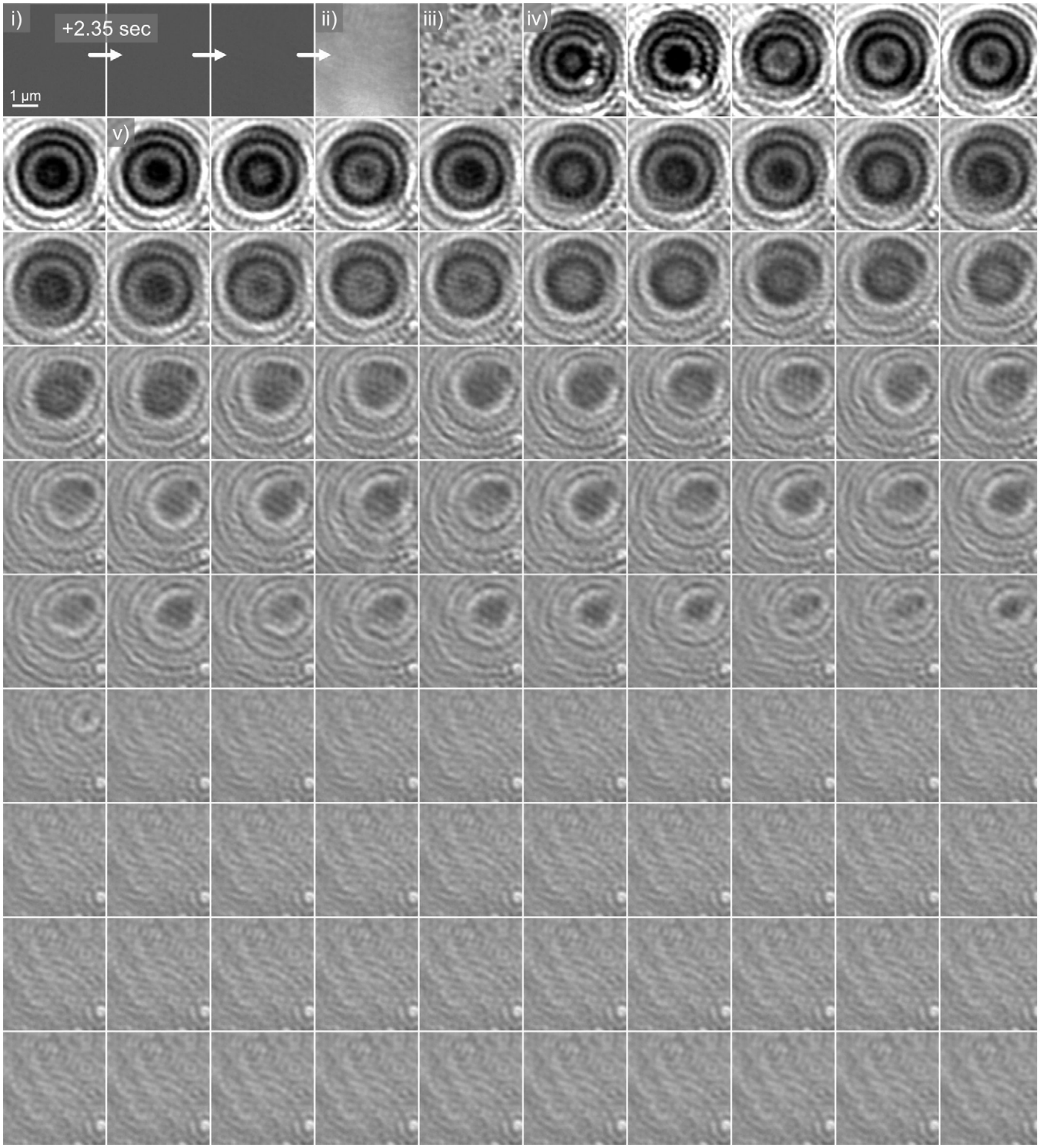

Supplementary Figure 21. Background-subtracted iSCAT images of the dissolution of liquid mesitylene droplets (time period: 235 ms). To a model system (i) of 1,4-dioxane/mesitylene (v/v 4:1; 100 µl) and TA (1.01 mg, 0.045 mmol) , water (1 equiv.) was added (ii),leading to the nucleation of dark contrast mesitylene droplets (iii). After their growth via Ostwald ripening, the droplets reach a stable state for a limited period (iv). Subsequently, solvent mixing proceeds, where 1,4-dioxane aids in the dissolution of water throughout the entire volume (v). Consequently, the polarity of the environment decreases, leading to a gradual dissolution of the mesitylene droplets. These interdependent processes manifest in the diminishing white signal surrounding the dark droplet (corresponding to water dissolution) and the reduction in size and decrease in black contrast of the droplet itself until complete dissolution (corresponding to mesitylene dissolution). The resulting ternary solvent system shows an enhanced contrast compared to the initial binary one due to inclusion of water. To enhance visibility, a subset of images was selected from the 50.000 acquired images. Specifically, every 500th image was chosen for display, resulting in a time difference of 2.35 sec between the displayed frames. Images were acquired at a speed of 4.7 ms per frame (212 fps), background-subtracted and 2x2 binned. The contrast is adjusted to 0.43 – 2.12. Scale bar (applies to all images), 1 µm.

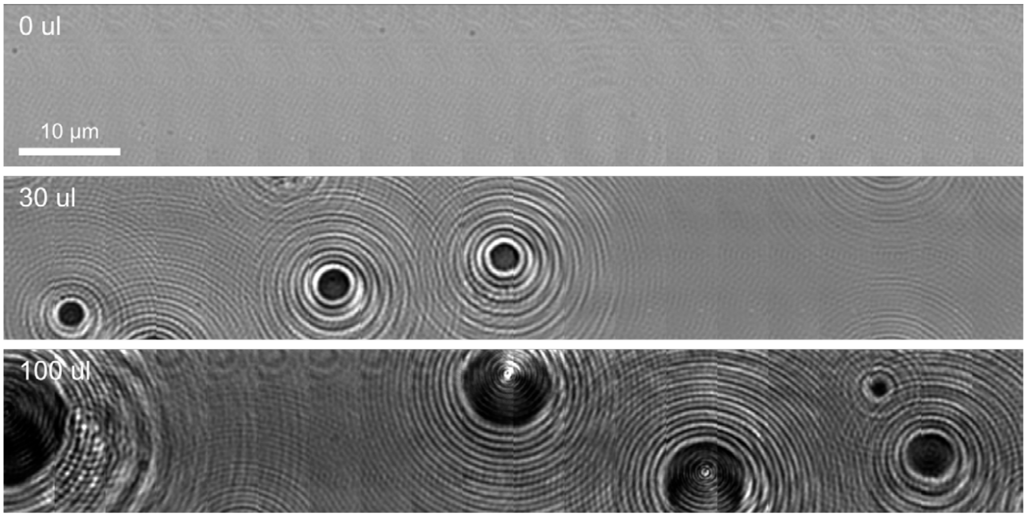

Supplementary Figure 22. To a 1,4-dioxane/water mixture (v/v 2:1) mesitylene is introduced incrementally increased from 0 µl over 30 µl to 100 µl. For each concentration, a 1D scan of the coverglass surface was recorded. The sample holder's stage was moved in 5 µm increments in the +y direction, covering a total distance of 100 µm. For each position, an iSCAT image was recorded. After background subtraction, the dynamic features are visualized which include the moving glass surface and all entities present on it. The concentration of mesitylene exhibited a clear correlation with the volume of the black droplets, confirming their identical nature. Furthermore, mesitylene was also found to be dissolved in the ternary solvent mixture, as evidenced by the decreasing background contrast. This decrease resulted from a lower refractive index difference between the coverglass and the solution after the inclusion of mesitylene in the mixture. The contrast is adjusted to 0.12 – 1.5. Scale bar (applies to all images), 10 µm.

Section 8. Droplet growth during liquid-liquid phase rearrangements

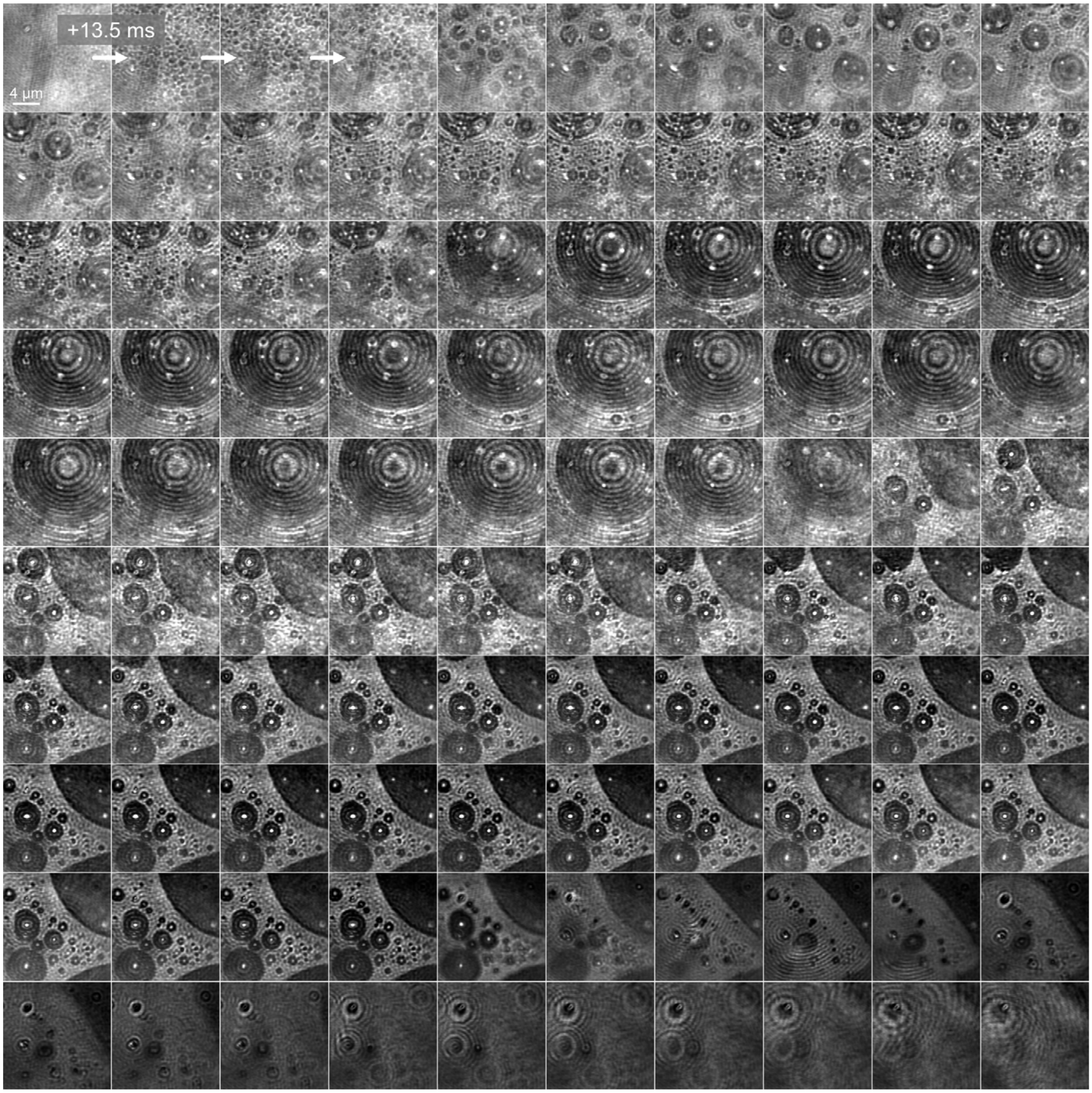

Supplementary Figure 23. Background-subtracted iSCAT images of the phase rearrangement processes during TA-TAPB COF formation with 3M HOAc (time period: 1.35 sec). The images show the nucleation, growth, presumably via Ostwald ripening, and dissolution of mesitylene droplets after catalyst addition. Images were acquired at a speed of 2.7 ms per frame (370 fps), background-subtracted, 2x2 binned and temporally averaged (5 consecutive frames). This results in a time difference of 13.5 ms between the displayed frames. The contrast is adjusted to 0.72 – 3.09. Scale bar (applies to all images), 4 µm.

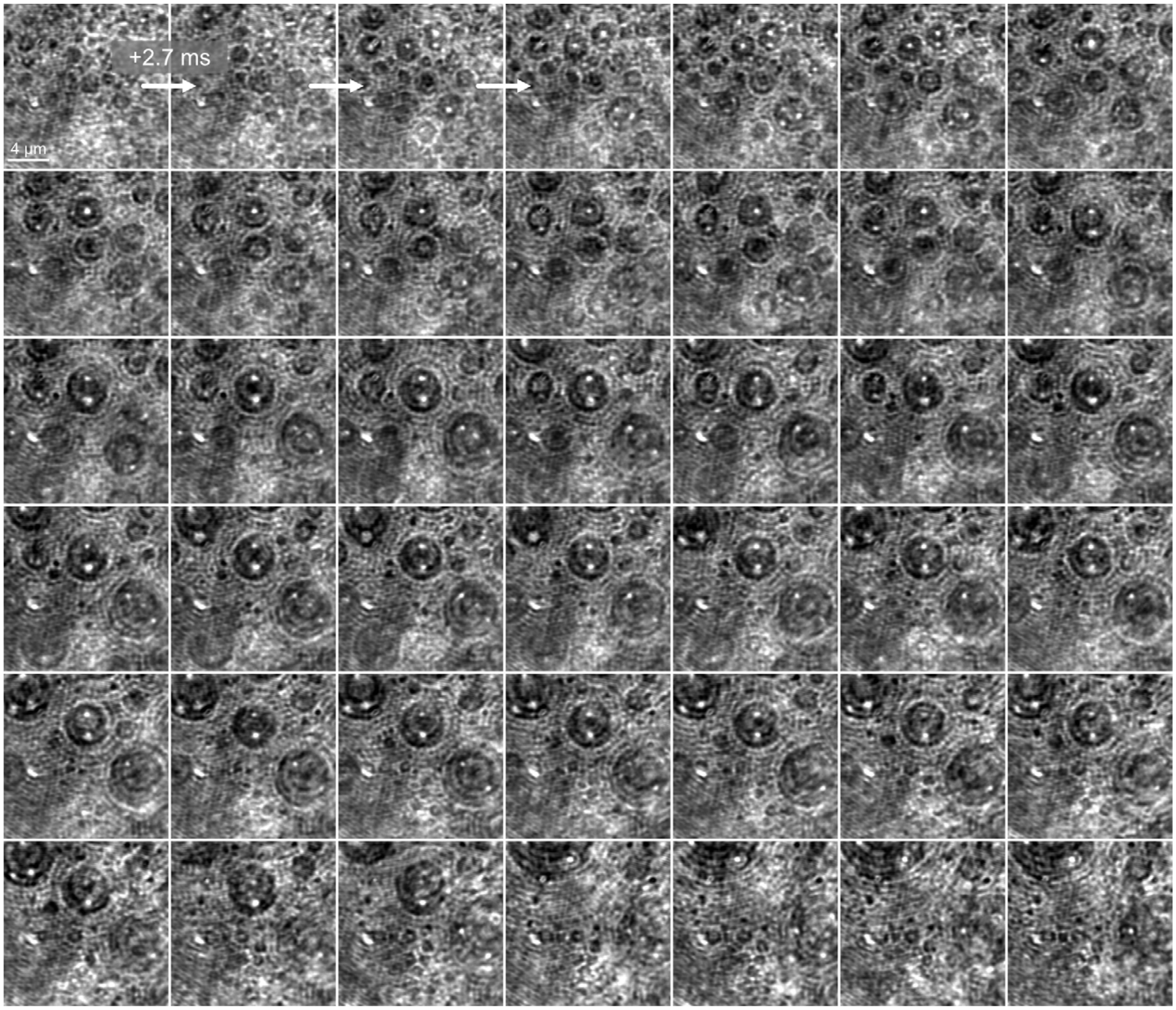

Supplementary Figure 24. Background-subtracted iSCAT images of the phase rearrangement processes during TA-TAPB COF formation with 3M HOAc (time period: 113.4 ms). The images show the growth, presumably via Ostwald ripening, and dissolution of the nucleated mesitylene droplets. Images were acquired at a speed of 2.7 ms per frame (370 fps), background-subtracted and 2x2 binned. The contrast is adjusted to 1.03 – 3.2. Scale bar (applies to all images), 4 µm.

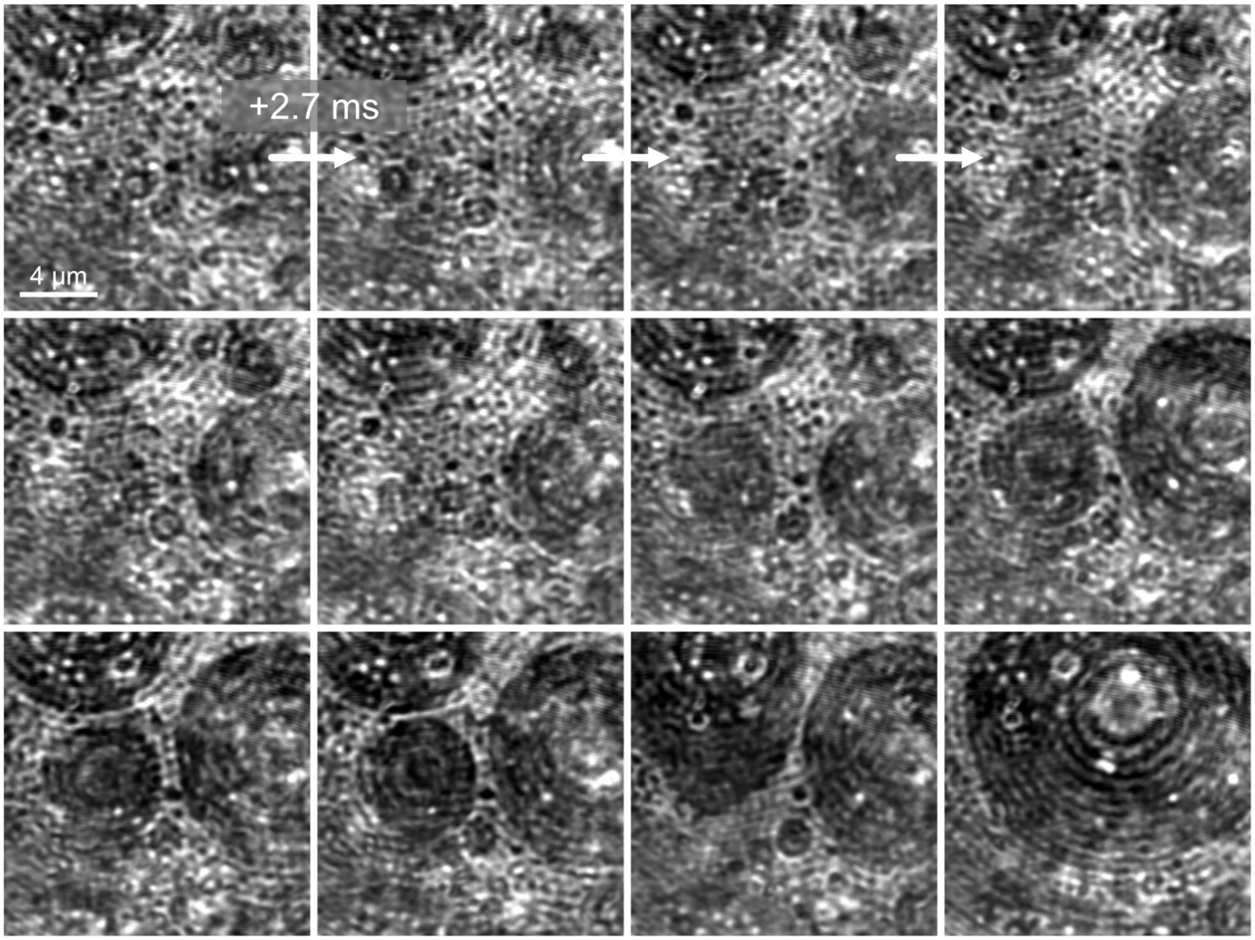

Supplementary Figure 25. Background-subtracted iSCAT images of the phase rearrangement processes during TA-TAPB COF formation with 3M HOAc (time period: 32.4 ms). The images show the growth, presumably via Ostwald ripening, of the mesitylene droplets. Images were acquired at a speed of 2.7 ms per frame (370 fps), background-subtracted and 2x2 binned. The contrast is adjusted to 0.82 - 3.2. Scale bar (applies to all images), 4 μm.

Section 9. Surfactant-free solvent structuring and liquid phase diagrams

The following discussion aims at elucidating the intricacies of ternary solvent systems, which comprise two immiscible solvents and an amphiphilic solvent, and their corresponding liquid phase behavior.

The liquid phase diagrams of these systems can vary significantly depending on the character of the solvents employed (e.g., the single phase region might occupy 5% or 75% of the phase diagram).[23,28,29] In addition to experimentally determining boundaries and areas of the their respective subregions, liquid phase diagrams can also be theoretically predicted by ab initio calculations.[29] The shape of the respective phase diagram is determined by the choice of solvents, while the position in the phase diagram and consequently the structuring are determined by the relative ratio of solvents in the ternary mixtures.[23,30] Notably, all participating solvents statistically contribute to each structured phase, implying the presence of, for instance, water within the oil phase, albeit in minor quantities.[31,32]

Importantly, in the case of the biphasic/multiphase regime, even in macroscopically separated phases, both phases can still exhibit structural features.[28,33,34] For example, Davis and co-workers examined the phase behavior and interfacial tensions of mixtures of hydrocarbon–brine–short chain alcohol (v/v 1:1:1) regarding salinity and temperature.[28,35,36] The authors discovered that increasing either the salinity or temperature could result in a change in the patterns of phase behavior, from a two-liquid-phase to a three-liquid-phase and further back to a two-liquid-phase equilibrium, as schematically shown in Supplementary Figure 26. The observed changes in phase behavior patterns for the surfactant-free ternary mixtures were found to be analogous to those previously reported by Winsor et al. in traditional surfactant-containing systems.[30,37] Furthermore, the three types of phase equilibria may align with Winsor I, III, and II systems, respectively.[28]

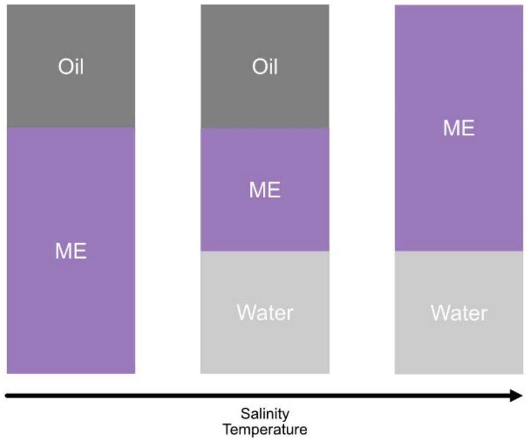

Supplementary Figure 26. Patterns of phase behavior of the oil-rich, water-rich, and alcohol-rich, microemulsion (ME) phases for equal-volume, surfactant-free mixtures of octane, brine (NaCl (aq.)), and *n*-propanol. Adapted from Ref. 36. The dependences of phase behavior on the salinity and temperature in surfactant-free ternary mixtures were found to be similar to those in surfactant-containing ones.[28]

Moreover, these experiments demonstrate that multiple parameters, in addition to the solvents, have a significant impact on the phase diagrams.

For instance, increasing the temperature extends the monophasic area while reducing the biphasic region.[38] Additionally, temperature changes can alter the relative areas of subregions and influence the size of solvent aggregates.[39] Furthermore, temperature changes can provide reversible control over phase behavior and the formation of nanodomains.[38]

Another considerable influence is achieved through the use of additives such as inorganic salts, which can cause significant alterations to the location of the subregion boundaries in the phase diagram.[40] In the context of surfactant-free emulsions, these additives have a comparable impact on interfacial surface tensions to that observed in systems containing surfactants (i.e. an increase in the tension of water-rich phase/oil-rich phase[41] and alcohol-rich phase/water-rich phase[33] and a decrease in the tension of alcohol-rich phase/oil-rich phase[33]). The effect of salt can also be described as the „salting out“ effect: the presence of salt reduces the solubility of other components in the water-rich phase.[42] For example, it has been reported for the system ethanol/octanol/water, that by salting out the hydrotrope (ethanol) from the water-rich phase, the polarity in the water phase is increased effectively pushing the system towards the two-phase region and the formation of larger oil-rich domains (octanol).[42] Other additives investigated include antagonistic salts. Here, it was reported that these additives decrease the interfacial tension of the oil/water interface[41] as well as the two-phase region.[43]

Another factor that can impact the solvent structure is the manner in which the solvent components are mixed (e.g., the order of addition of the components; step-by-step or one-shot addition).[34,44] It should be noted here that the kinetics of their formation are typically fast (< 1s), but can be slowed down.[45]

In recent literature, one of the most studied structuring effects has been the thermodynamically stable surfactant-free microemulsions (SFME). The term SFME is one of a multitude of names that include detergentless microemulsions, “pre-Ouzo“, micellar-like structural fluctuations, mesoscale solubilization, and ultraflexible microemulsions (UFMEs).[38] The thermodynamic stability of SFMEs can be explained by the establishment of an equilibrium between the repulsive hydration force, attributed to solvation effects, acting as a deterrent to the coalescence of water-rich and hydrophobe-rich domains, and entropy, which propels the system towards the formation of smaller domains.[46] It is noteworthy that SFMEs and mesoscopic structuring can also form in binary solvent mixtures.[47] Furthermore, SFMEs have been designed where the structuring can be specifically controlled by external stimuli, e.g., by CO_2 or temperature.[39,48] In the case of a phase inversion of an o/w emulsion, the o/w domains gradually grow and interconnect, initially forming a bicontinuous structure and subsequently an oil-continuous domain with w/o domains.[28] The sizes of solvent aggregates exhibit considerable variability based on formulation, mixture type, and external parameters, ranging from approximately 1 nm[38] to over 100 nm[23,44] Here, it has been demonstrated that multi-scale aggregates can coexist within the system.[23,34,38] The role of SFMEs in chemical reactivity is complex, with reports indicating substantial impacts on kinetics, yields, and local reactant concentrations.[47,49] The complexity increases as the reactants themselves influence the system and phase diagram.

Furthermore, it should be noted that there are several related concepts based on surfactant-free solvent structuring (covering pre-micellar aggregates to microscale entities), such as solvent shifting/displacement,[50] nanoprecipitation[44] or facilitated hydrotropy[51].

Section 10. iSCAT measurements of COF formation in a binary solvent system without solvent structuring

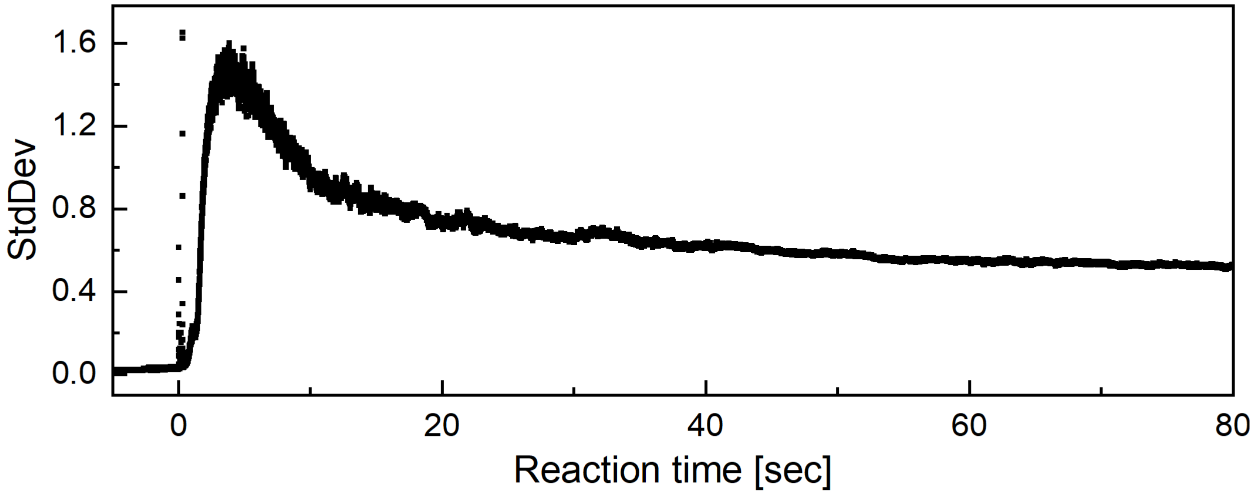

Supplementary Figure 27. Temporal evolution of the StdDev of the iSCAT images acquired during the formation of TA-TAPB COF in the binary solvent system 1,4-dioxane and 3M aqueous acetic acid (no mesitylene). Images were acquired at a speed of 2.2 ms per frame (455 fps). Images were background-subtracted and 2x2 binned.

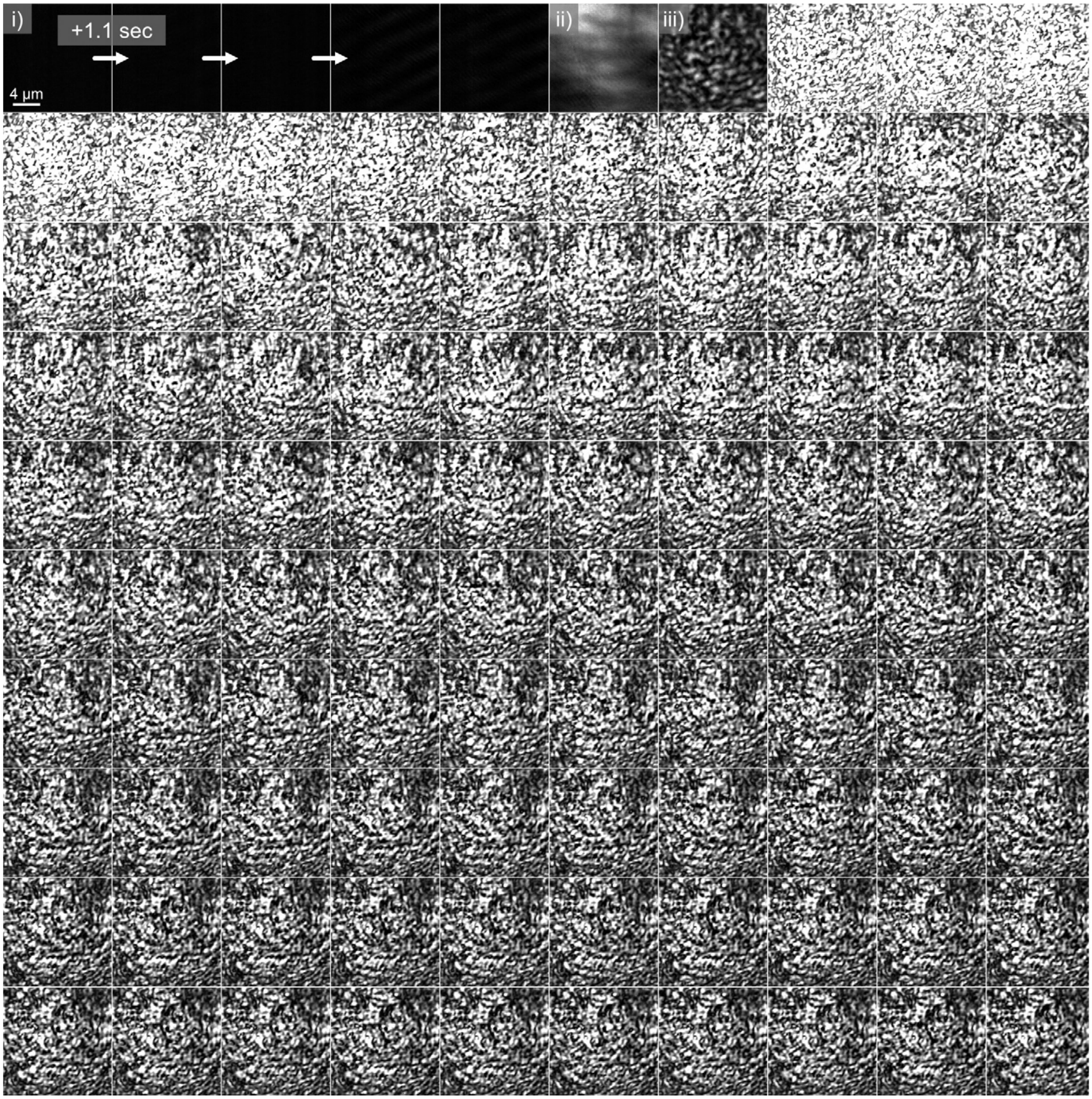

Supplementary Figure 28. Background-subtracted iSCAT images during TA-TAPB COF formation in the binary solvent system 1,4-dioxane and 3M aqueous acetic acid (time period: 110 sec). To the initial reactant solution (i ; TA and TAPB in 1,4-dioxane), the aqueous catalyst mixture is added (ii) which initiates the reaction and precipitation on the surface (iii).. Images were acquired at a speed of 2.2 ms per frame (455 fps), background-subtracted and 2x2 binned. To enhance visibility, a subset of images was selected from the 50.000 acquired images. Specifically, every 500th image was chosen for display, resulting in a time difference of 1.1 sec between the displayed frames. The contrast is adjusted to 0.92 – 2.51. Scale bar (applies to all images), 4 µm.

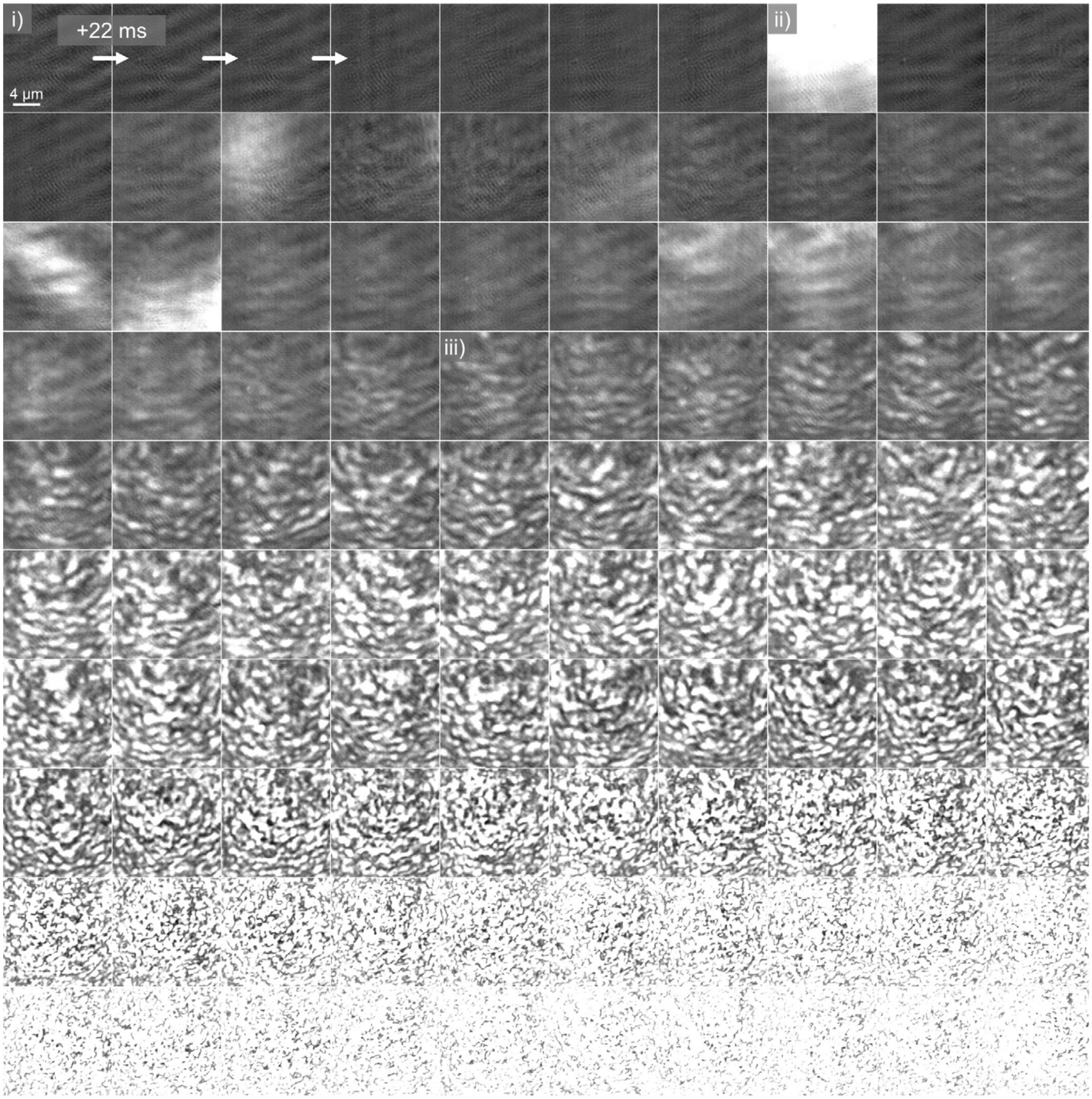

Supplementary Figure 29. Background-subtracted iSCAT images during TA-TAPB COF formation in the binary solvent system 1,4-dioxane and 3M aqueous acetic acid (time period: 2.2 sec). To the initial reactant solution (i ; TA and TAPB in 1,4-dioxane), the aqueous catalyst mixture is added (ii). Upon addition, diffuse alterations in the reflectivity are observed, stemming from the introduction of water (higher refractive index difference to the glass surface compared to 1,4-dioxane) and the resulting solvent mixing processes. However, no phase separation phenomena or nucleation of liquid droplets is captured by the imaging process. In the following, a direct transition to the reaction is imaged (iii). Images were acquired at a speed of 2.2 ms per frame (455 fps), background-subtracted and 2x2 binned. To enhance visibility every 10th image was chosen for display, resulting in a time difference of 22 ms between the displayed frames. The contrast is adjusted to 0.8 – 1.5. Scale bar (applies to all images), 4 µm.

Section 11. Solvent systems meeting the conditions for surfactant-free emulsification commonly used in COF synthesis and systems currently not used in COF synthesis

Hydrotrope	Hydrophobe	Hydrophile	Composition	Reference
1,4-Dioxane	Mesitylene	Water/Acetic Acid (6M)	9/1/1	[4]
1,4-Dioxane	Dichlorethane	Water/Acetic Acid (8M)	17/17/1	[45]
1,4-Dioxane	Toluene	Water/Acetic Acid (9M)	2/4/1	[46]
NMP	TCB	Water/Acetic Acid (6M)	8/2/1	[47]
NMP	o-DCB	Water/Acetic Acid (6M)	8/2/1	[48]
n-Butanol	Mesitylene	Water/Acetic Acid (6M)	5/5/1	[49]
n-Butanol	o-DCB	Water/Acetic Acid (6M)	5/5/1	[50]
n-Propanol	Dichlorethane	Water/Acetic Acid (8M)	17/17/1	[45]
Ethanol	Mesitylene	Water/Acetic Acid (6M)	5/5/1	[51]
Ethanol	o-DCB	Water/Acetic Acid (9M)	13/13/2	[52]
Methanol	Chloroform	Water/Acetic Acid (6M)	2/3/1	[53]
Benzyl alcohol	Mesitylene	Water/Acetic Acid (6M)	5/5/1	[54]
DMAC	o-DCB	Water/Acetic Acid (6M)	6/2/1	[55]

Supplementary Table 4. Overview of different ternary solvent systems that are commonly used in the synthesis of COFs and consist of a hydrotrope, hydrophobe and hydrophile, fulfilling the conditions for the creation of surfactant-free emulsions. Abbreviations: N-methyl-2-pyrrolidinone (NMP), 1,2,4-Trichlorobenzene (TCB), o-Dichlorobenzene (o-DCB), Dimethylacetamide (DMAC).

Hydrotrope	Hydrophobe	Hydrophile	Reference
THF	Anisole	Water	[25]
Ethanol	Anisole	Water	[25]
Acetone	Anisole	Water	[25]
THF	Limonene	Acetonitrile	[25]
Ethanol	Limonene	Acetonitrile	[25]
Acetone	Limonene	Acetonitrile	[25]
THF	Limonene	Water	[25]
Ethanol	Limonene	Water	[25]
Acetone	Limonene	Water	[25]
Oleic acid	n-Propanol	Water	[56]
Ethanol	trans-Anethanole	Water	[20]
Ethanol	1-Octanol	Water	[57]
1-Propanol	Isopentyl-Acetate	Water	[54]
1-Octanol	Ethanol	Glycerol	[55]
Diethyl Adipate	Tetrahydrofurfuryl alcohol	Deep eutectic solvent: Urea–Choline Chloride (2/1)	[55]
Diethyl Adipate	Tetrahydrofurfuryl alcohol	Deep eutectic solvent: Ethylene Glycol–Choline Chloride (4/1)	[55]
Methyl Salicylate	Ethanol	Water	[60]
2-Propanol	Toluene	Water	[61]
2-Propanol	Benzene	Water	[62]
Ethanol	Benzene	Water	[63]
Ethanol	Furaldehyde	Water	[64]
N,N-dimethyl formamide	Furaldehyde	Water	[65]
Hexanol	Cyclohexane	Water	[66]
1-Propanol	n-Hexane	Water	[67]
n-Butanol	n-Hexane	Water	[68]
Dichloromethane	Ethanol	Water	[43]

Supplementary Table 5. Overview of ternary solvent systems not commonly used in COF synthesis that consist of a hydrotrope, hydrophobe and hydrophile and that were reported to form surfactant-free emulsions. Abbreviation: Tetrahydrofuran (THF).

Section 12. iSCAT induction periods of TA TAPB COF formation employing different catalyst and solvent conditions

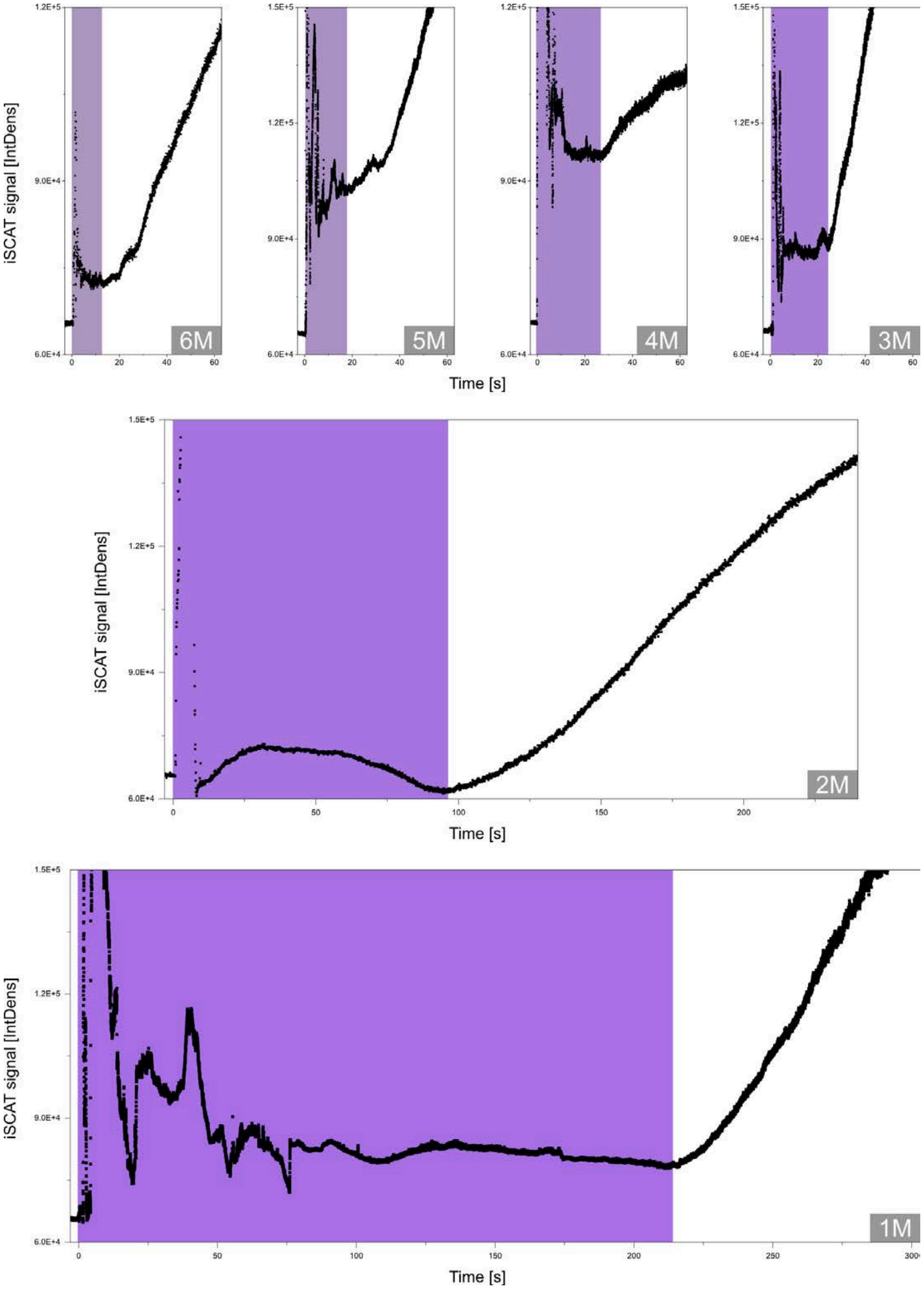

Supplementary Figure 30. iSCAT traces of the TA-TAPB COF reaction in 1,4-dioxane/mesitylene (9:1 v/v) with different concentrations of aqueous HOAc employed as catalyst (1 equiv.). The colored region is depicting the induction periods (defined as time period between catalyst addition and integrated iSCAT signal onset). Here, as a figure of merit for the reaction progress by

iSCAT, the integrated density (IntDens) of the background corrected images is taken (summing up of all pixel values in an image) as it is better suited for determining the system-wide growth. Up to three traces have been averaged for Fig. 2 and Supplementary Figure 35 (1M, 3M and 6M show here correspond to the montages above).

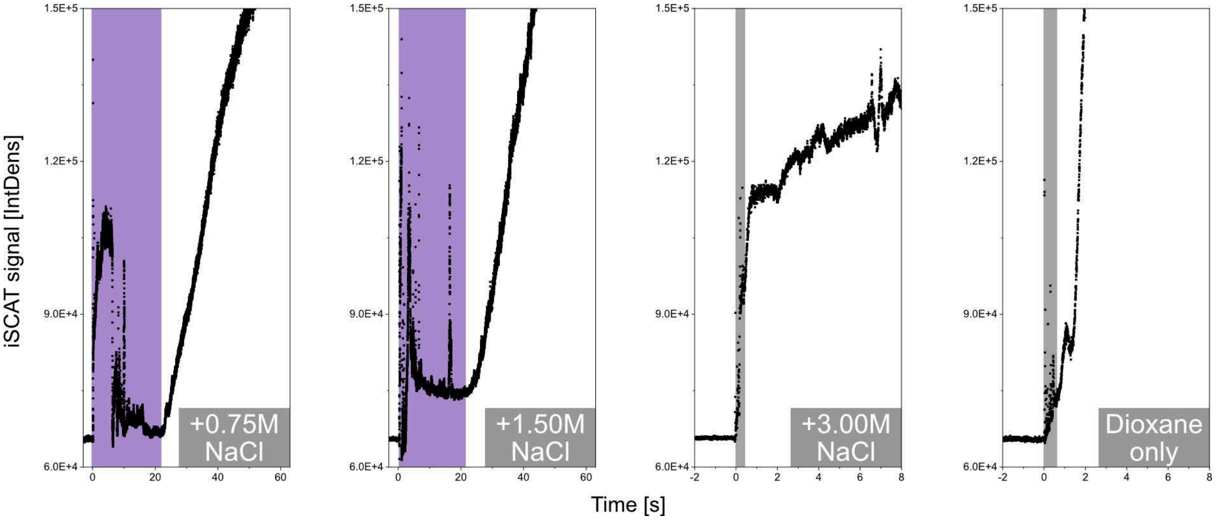

Supplementary Figure 31. iSCAT traces of the TA-TAPB COF reaction in 1,4-dioxane/mesitylene (9:1 v/v) with 3M aqueous HOAc and different amount of NaCl employed as catalyst (1 equiv.) or in 1,4-dioxane (10 equiv.) with 3M aqueous HOAc employed as catalyst (1 equiv.). The colored region is depicting the induction periods (defined as time period between catalyst addition and integrated iSCAT signal onset). Here, as a figure of merit for the reaction progress by iSCAT, the integrated density of the background corrected images is taken (summing up of all pixel values in an image) as it is better suited for determining the system-wide growth. Up to three traces have been averaged for Fig. 2 and Supplementary Figure 35 (dioxane only corresponds to the montage shown above).

Section 13. Complementary data to Figures 3 and 4

a) Corresponding PXRD patterns to Figure 2b

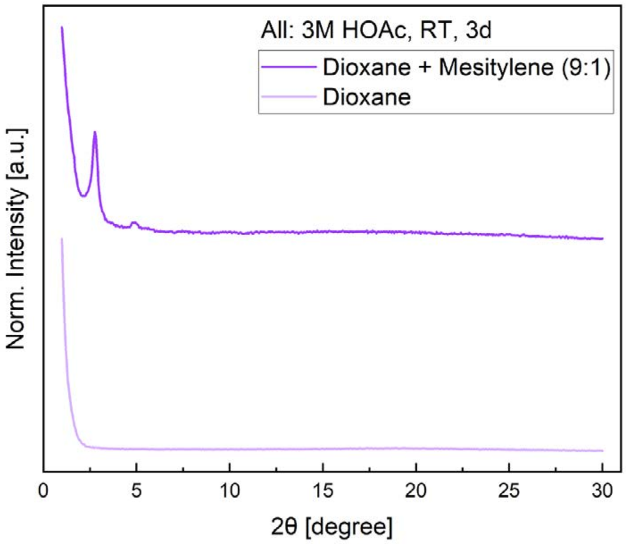

Supplementary Figure 32. Normalized PXRD patterns of TA-TAPB COF synthesized in a mixture of 1,4-dioxane and mesitylene (v/v 9:1) and in 1,4-dioxane (10 equiv.) (both RT, 1 equiv. of 3M HOAc, 3 d). Powders obtained in the binary solvent system of 1,4-dioxane and 3M HOAc shows no crystallinity while synthesis in the ternary solvent system results in considerably enhanced crystalline features. Snippets of these spectra are displayed in Fig. 3b.

b) Effect of the inclusion of mesitylene in the reaction mixture at different reaction times

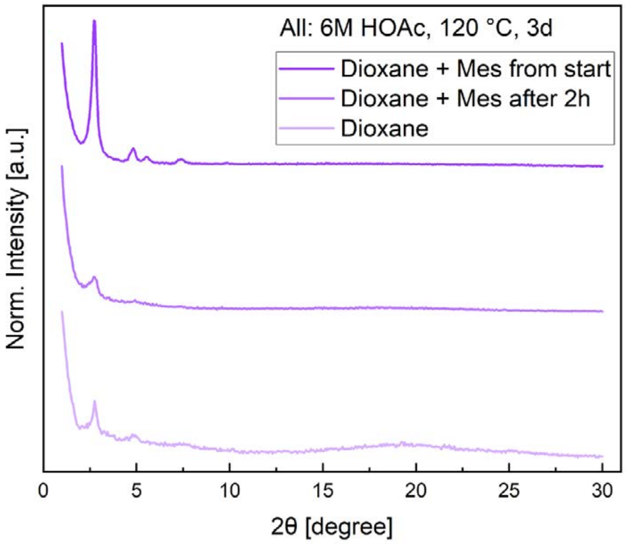

Supplementary Figure 33. Normalized PXRD patterns of TA-TAPB COF synthesized in a reactant mixture of TA and TAPB solute in: 1,4-dioxane only (10 equiv.); initially only 1,4-dioxane (9 equiv.), then addition of mesitylene (1 equiv.) 2 h after reaction start (i.e., catalyst addition); 1,4-dioxane/mesitylene (v/v 9:1). All: 1 equiv. of 6M HOAc, 120°C, 3 d. The crystallinity of the obtained COF powder is significantly influenced by the presence of mesitylene only when it is included in the reaction mixture from the beginning.

c) Corresponding PXRD patterns to the crystallinity plot in Figure 3c

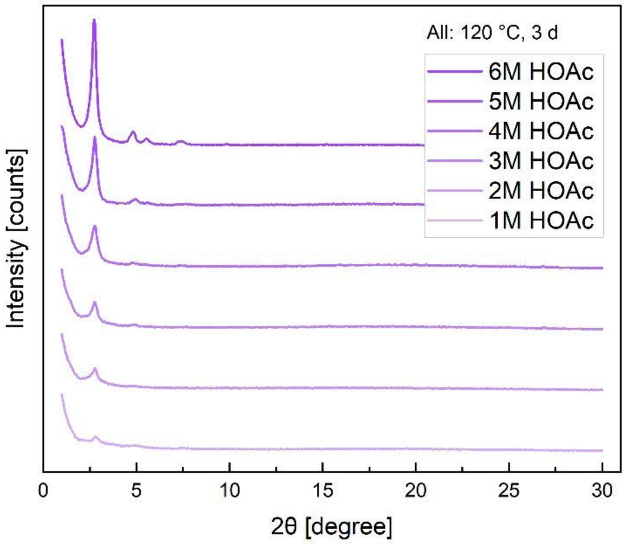

Supplementary Figure 34. PXRD patterns of TA-TAPB COF synthesized with 1-6M HOAc (all: 1,4-dioxane/mesitylene/HOAc v/v 9:1:1; 120 °C, 3 d). Increase in catalyst concentration correlates with an increase in crystallinity of the obtained COF powder. The area below all crystalline peaks is calculated for each diffractogram after background correction and plotted in Fig. 3c. While the reactions have been conducted at 120 °C, the initial reaction started in the seconds upon solvent mixing at room temperature.

d) iSCAT induction periods of TA-TAPB COF formation

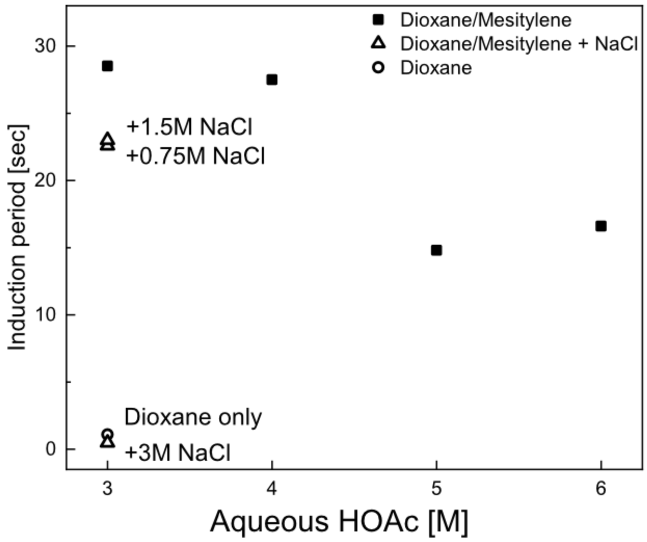

Supplementary Figure 35. iSCAT induction periods for TA-TAPB COF formation in different reaction conditions (all: RT; solvent system either 1,4-dioxane/mesitylene/aqueous catalyst mixture v/v 9:1:1 or 1,4-dioxane/aqueous catalyst mixture v/v 10:1). The induction period is defined as the time span between catalyst addition and start of the integrated iSCAT signal onset .

e) 100 Peak height as Figure of merit for trend shown in Fig. 4a

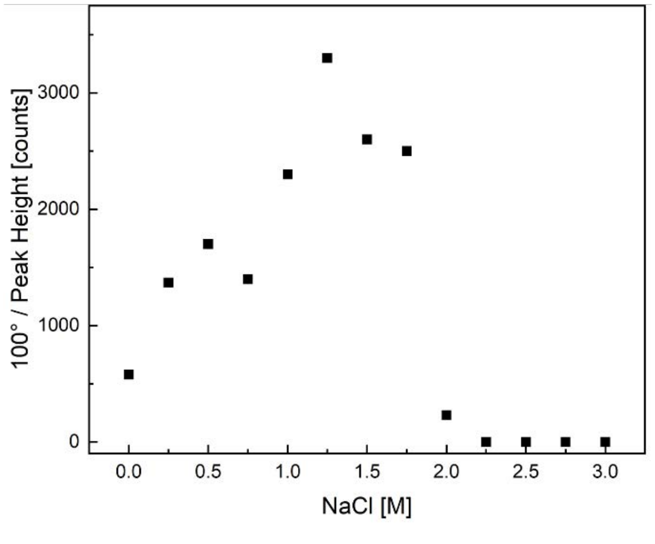

Supplementary Figure 36. The plot represents a qualitative trend, visible in Fig. 4a, calculated based on the peak height of the 100 reflection.

Section 14. Effect of including NaCl into the catalyst mixture on the solvent restructuring processes

To confirm the salting-out effect of NaCl on the solvent phases in the TA-TAPB COF system, we conducted iSCAT measurements without HOAc to prevent initiating the reaction. While addition of water to the reactant solution resulted in a stable ternary solvent mixture, upon aqueous NaCl addition, water-rich droplets break out of the solution after several minutes and attach on the surface. After 15 min water-rich domains have accumulated on the surface with smaller diameter upon higher salt concentration which we attribute to the increase in surface tension. This shows the role of increasing ionic concentration in structuring the solvent mix.

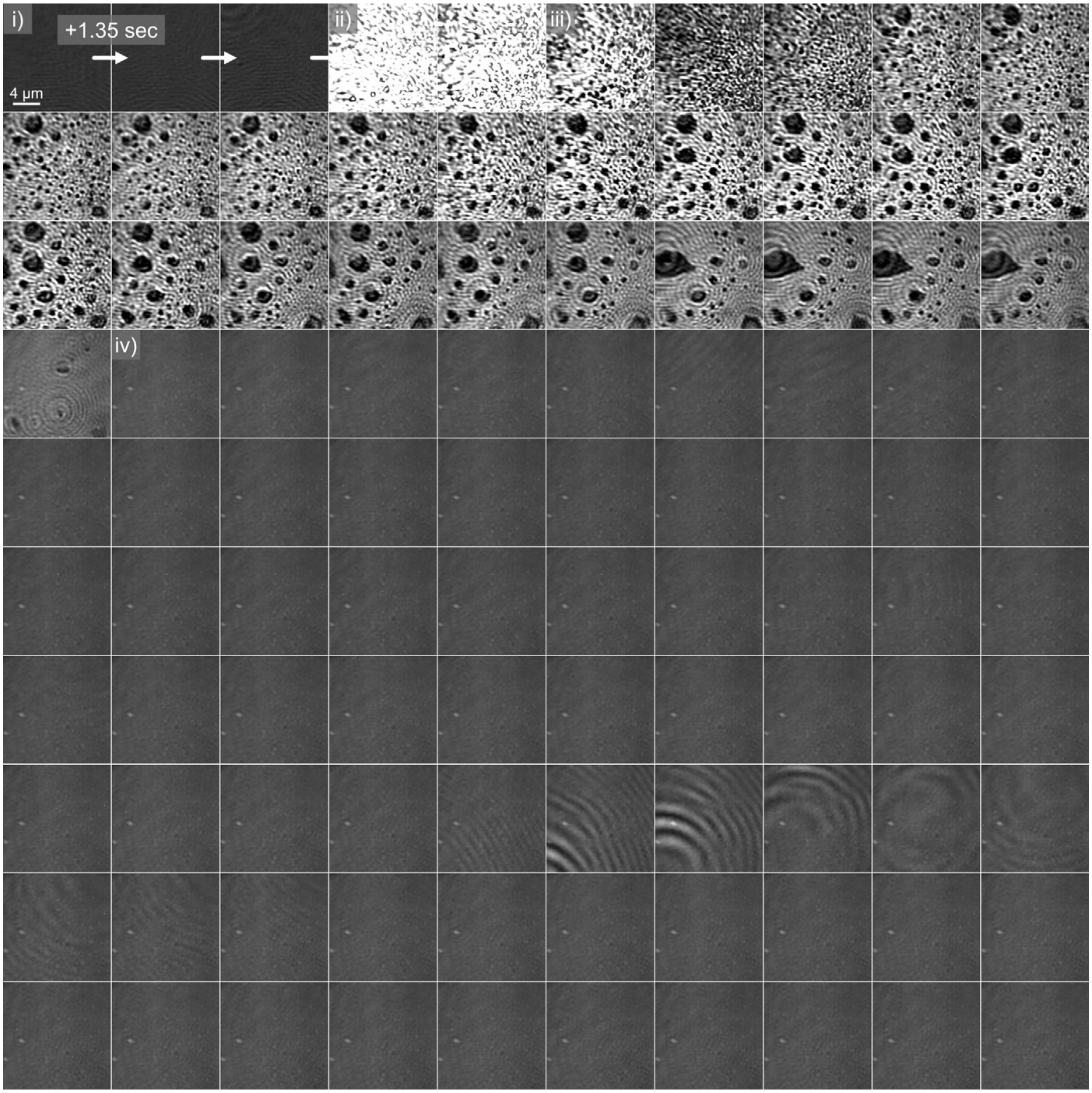

Supplementary Figure 37. Background-subtracted iSCAT images of the solvent rearrangements in the TA-TAPB reactant mixture upon addition of water (time period: 135 sec). To the initial reactant solution (i ; TA and TAPB in 1,4-dioxane/mesitylene v/v 9:1), water (1 equiv.) is added (ii). Subsequently, phase rearrangement processes and nucleation of black contrast mesitylene droplets (iii) are imaged. The droplets dissolve into solution and a ternary solvent mixture is formed (iv). Images were acquired at a speed of 2.7 ms per frame (371 fps), background-subtracted and 2x2 binned. To enhance visibility, a subset of images was selected from the 50.000 acquired images. Specifically, every 500th image was chosen for display, resulting in a time difference of 1.35 sec between the displayed frames. The contrast is adjusted to 0.71 – 2.08. Scale bar (applies to all images), 4 µm.

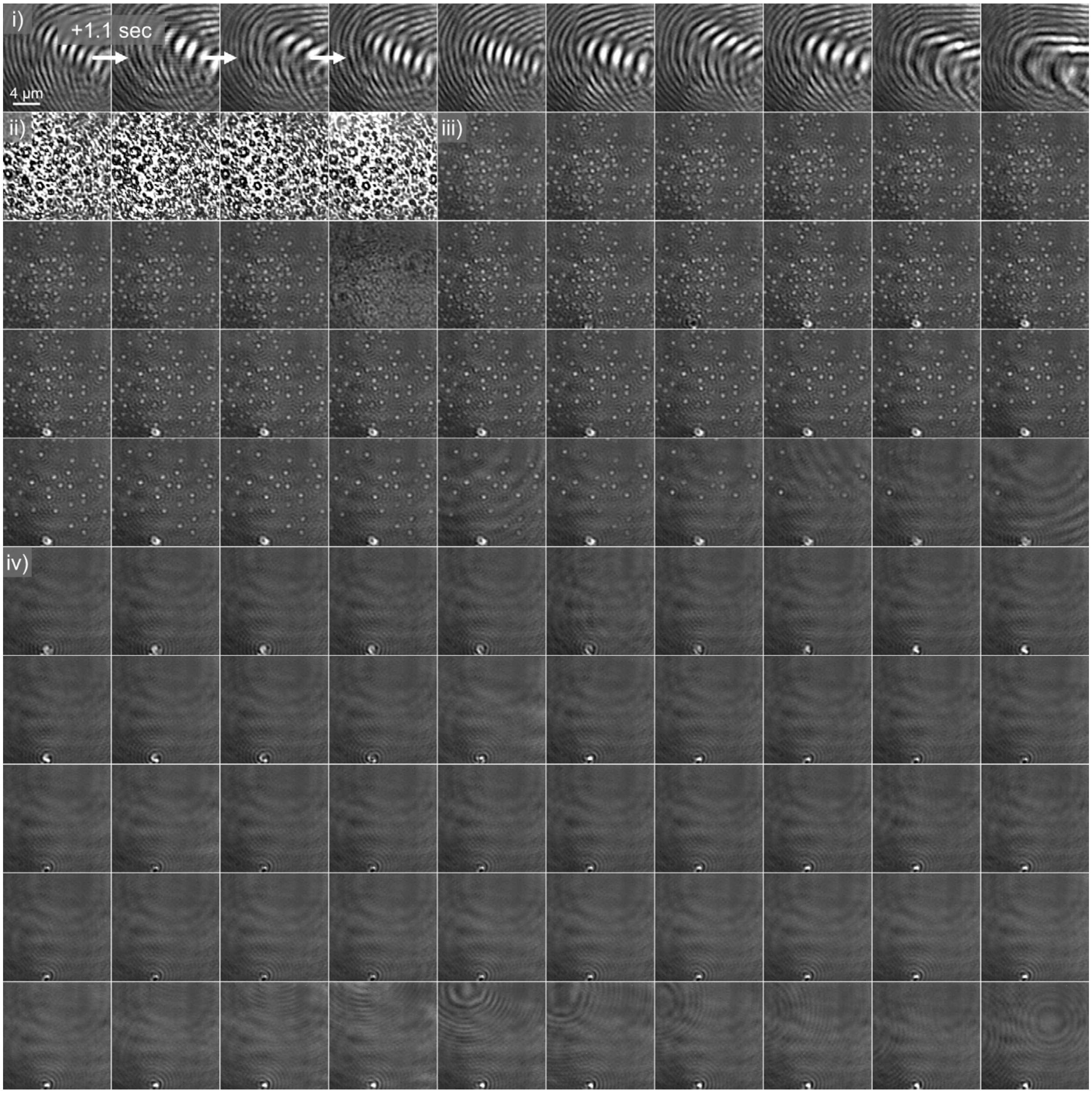

Supplementary Figure 38. Background-subtracted iSCAT images of the solvent rearrangements in the TA-TAPB reactant mixture upon addition of aqueous 1.5M NaCl (time period: 110 sec). To the initial reactant solution (i ; TA and TAPB in 1,4-dioxane/mesitylene v/v 9:1), where some undissolved reactants are visible as out-of-focus PSFs, 1.5M NaCl (1 equiv.) is added (ii). In the subsequent phase rearrangement processes, dark-contrast mesitylene droplets nucleate in the water-rich, high-contrast environment (ii). After the dissolution of both phases into solution, white-contrast entities are imaged on the surface for a limited time (iii; see in greater detail Supplementary Figure 39). Finally, the images show a ternary solvent mixture where floating entities exist in solution (iv). Images were acquired at a speed of 2.2 ms per frame (456 fps), background-subtracted and 2x2 binned. To enhance visibility, a subset of images was selected from the 50.000 acquired images. Specifically, every 500th image was chosen for display, resulting in a time difference of 1.1 sec between the displayed frames. The contrast is adjusted to 0.68 – 1.74. Scale bar (applies to all images), 4 µm.

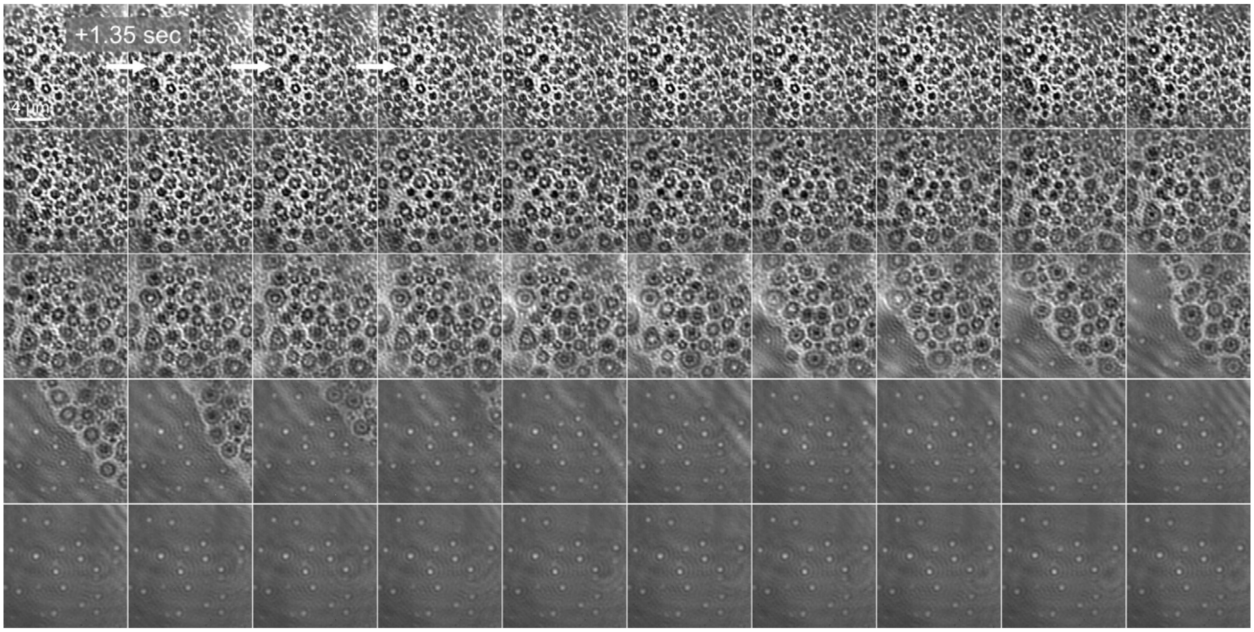

Supplementary Figure 39. Background-subtracted iSCAT images of the solvent rearrangements in the TA-TAPB reactant mixture upon addition of aqueous 1.5M NaCl (time period: 110 ms). Here, the dissolution of the water-rich, white-contrast phase and the dark-contrast mesitylene droplets is shown in higher temporal resolution (transition from ii to iii in Supplementary Figure 38). Images were acquired at a speed of 2.2 ms per frame (456 fps), background-subtracted and 2x2 binned. The contrast is adjusted to 0.5 – 2.0. Scale bar (applies to all images), 4 µm.

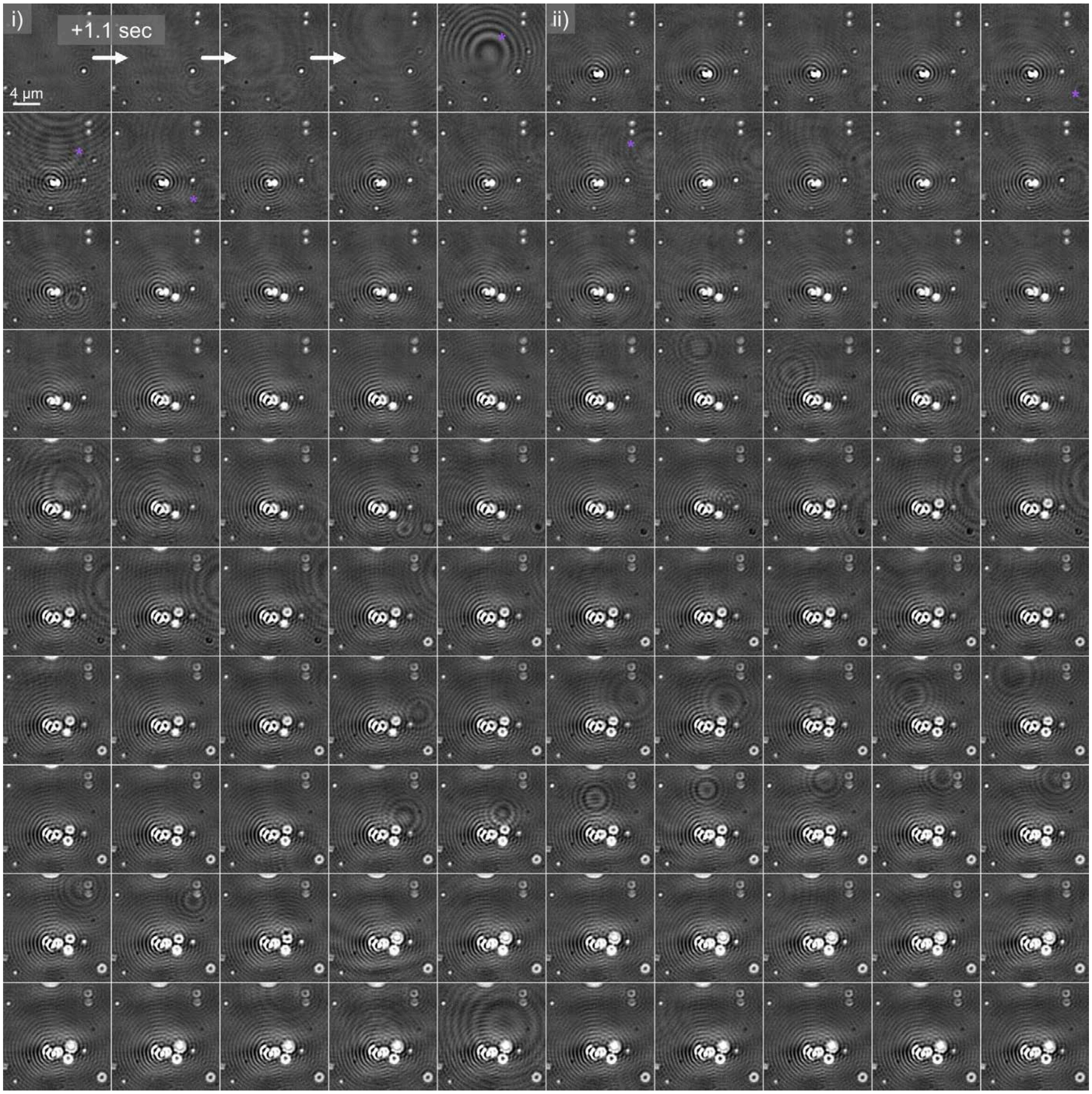

Supplementary Figure 40. Background-subtracted iSCAT images taken 3 minutes after adding aqueous 3M NaCl (1 equiv.) to the TA-TAPB reactant mixture (TA and TAPB in 1,4-dioxane/mesitylene v/v 9:1) (time period: 110 sec). In the ternary solvent system formed previously by addition of aqueous NaCl (i), water-rich droplets have nucleated in solution. They are visible in the out-of-focus PSFs (some examples in the initial images have been marked with * for guidance), which attach on the surface during the measurement and give rise to white-contrast, water-rich droplets (ii). Images were acquired at a speed of 13.7 ms per frame (73 fps), background-subtracted and 2x2 binned. To enhance visibility, a subset of images was selected from the 8.000 acquired images. Specifically, every 80th image was chosen for display, resulting in a time difference of 1.1 sec between the displayed frames. The contrast is adjusted to 0.79 – 1.63. Scale bar (applies to all images), 4 µm.

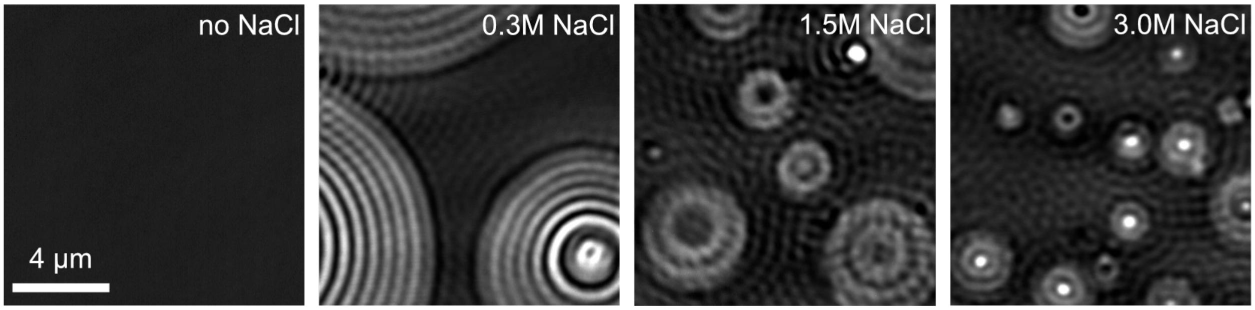

Supplementary Figure 41. To the reaction solution (TA/TAPB in 1,4-dioxane/mesitylene v/v 9:1) water or aqueous NaCl in different concentrations (1 equiv.) were added. Here, the background corrected iSCAT images show the coverglass surface after 15 min. In case of the NaCl mixtures, stable water-rich droplets have nucleated in solution and attached to the surface (see also Supplementary Figure 40). The size of the droplets inversely correlates with the concentration of NaCl, which we attribute to the increasing surface tension of the water-rich phase with increasing NaCl. Scale bar (applies to all images), 4 μm. Contrast is adjusted to 0.66 - 3.12.

Section 15. Characterization of TA TAPB COF synthesized with IAC approach at RT

a) Indexing of TA-TAPB COF obtained with 3M HOAc/1.5M NaCl as catalyst mixture at RT

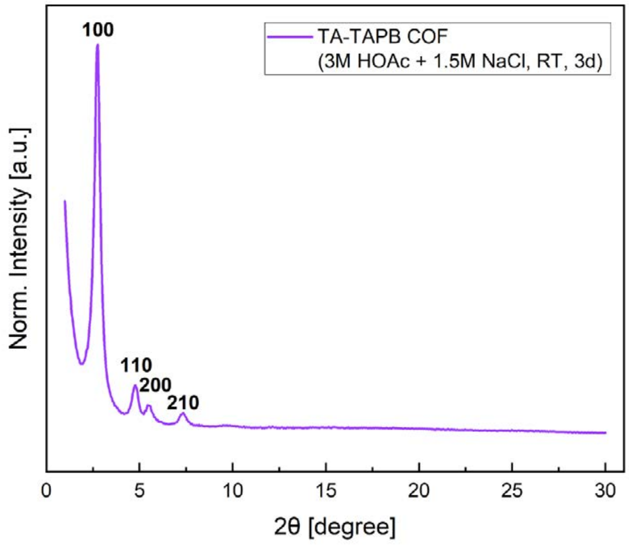

Supplementary Figure 42. Normalized PXRD pattern of TA-TAPB COF synthesized in 1,4-dioxane/mesitylene (v/v 9:1) and 3M HOAc / 1.5M NaCl as catalyst mixture (1 equiv.; RT, 3 d).

b) Pore accessibility of TA-TAPB COF synthesized with 3M HOAc/1.5M NaCl as catalyst mixture at RT

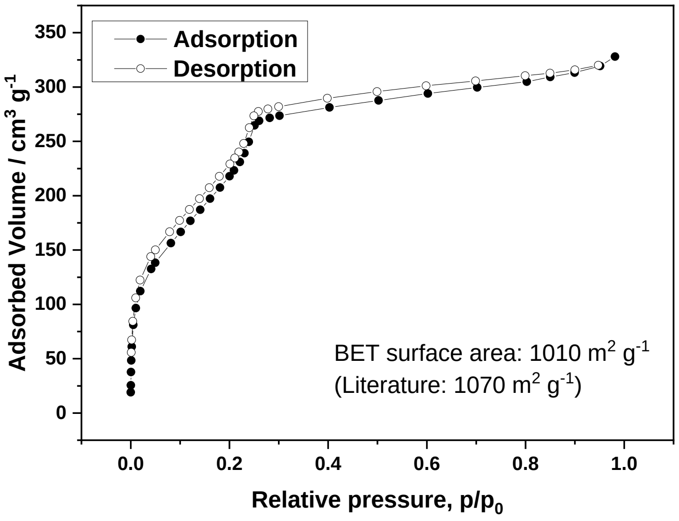

Supplementary Figure 43. Nitrogen sorption measurement at 77K of TA-TAPB COF synthesized in 1,4-dioxane/mesitylene (v/v 9:1) and 3M HOAc / 1.5M NaCl as catalyst mixture (1equiv.; RT, 3 d). A type IVb isotherm was obtained, characteristic of mesoporous materials with pore sizes < 5 nm,[56] exhibiting two steep and well-defined nitrogen uptakes at relatively low partial pressure ($p/p_0 < 0.15$ and up to 190 cm^3g^{-1}). This and the BET surface area of 1010 m^2/g^{-1} are in good agreement with our previous reported values (1070 m^2/g^{-1} for TA-TAPB COF synthesized at 120°C in 1,4-dioxane/mesitylene/6M HOAc, v/v 9:1:1)[1].

c) Morphology of TA-TAPB COF synthesized with NaCl in catalyst mixture

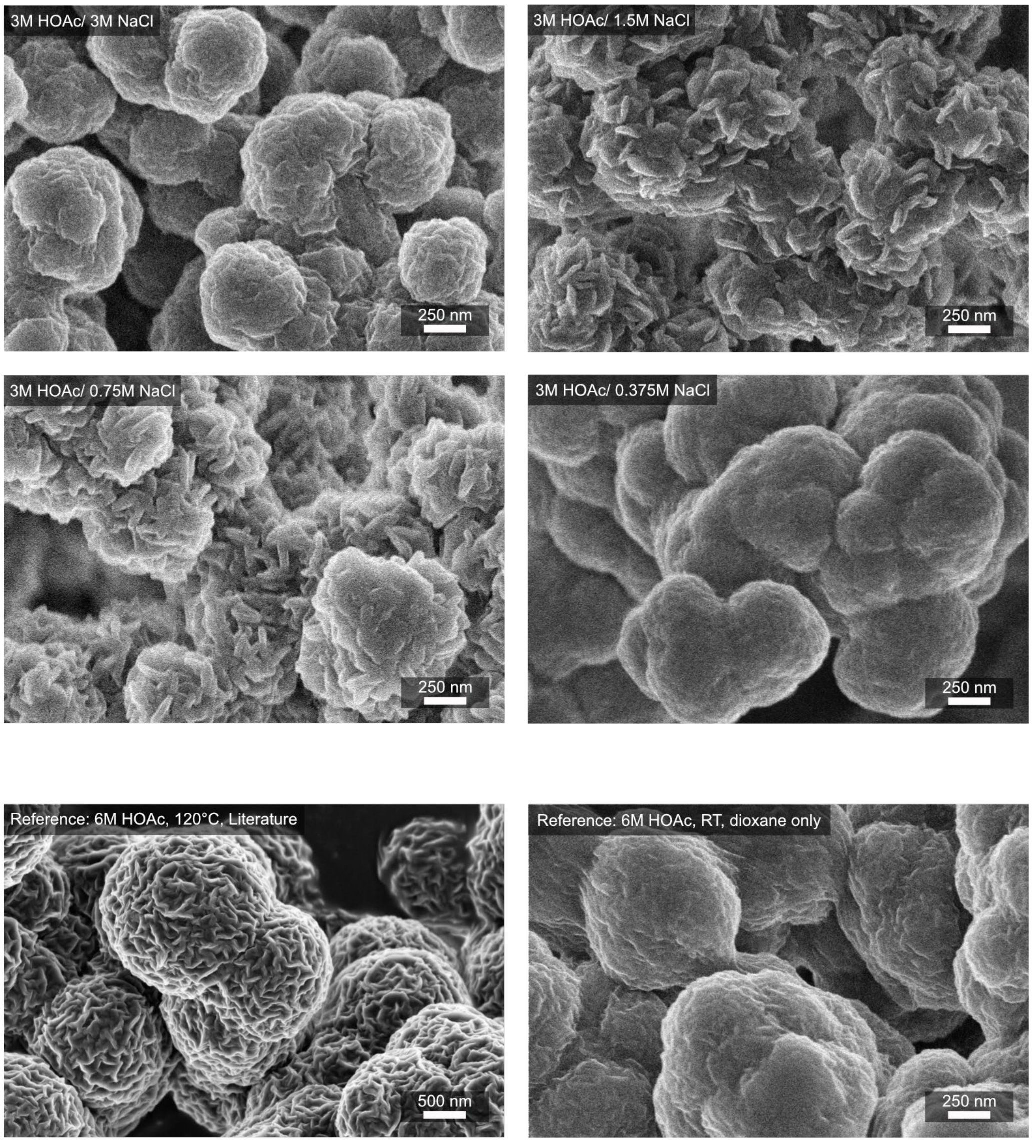

Supplementary Figure 44. Top, SEM images depicting the morphology of TA-TAPB COF synthesized in 1,4-dioxane/mesitylene/aqueous catalyst mixture (v/v 9:1:1), including different amount of NaCl in the catalyst mixture (RT, 3 days). Bottom, Reference SEM image of TA-TAPB COF synthesized under high temperature (120 °C) in literature conditions (1,4-dioxane/mesitylene/6M HOAc, v/v 9:1:1; 3 d)[1] and TA TAPB COF synthesized in the binary solvent system 1,4-dioxane and aqueous acetic acid (v/v 10:1; 6M HOAc, RT, 3 d). The spherical-like morphology reported in literature is maintained for low (0.375M) and high (3M) NaCl concentrations with less pronounced individual crystals. For 0.75M and 1.5M NaCl, the structures exhibit a large degree of interconnection, and the individual crystallites are more particulate and larger in size.

Section 16. IAC approach - required conditions, generalizability, upscaling and effect on the COF crystallization

a) Effect of NaCl addition on COF crystallinity in a binary solvent system

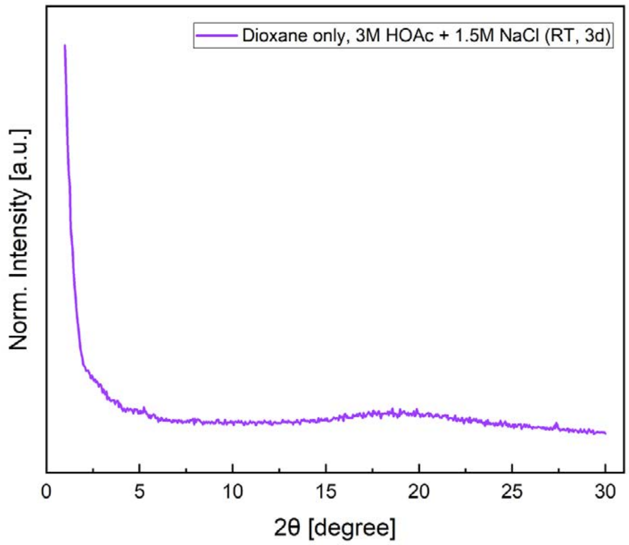

Supplementary Figure 45. Normalized PXRD patterns of TA-TAPB COF synthesized in 1,4-dioxane and 3M HOAc / 1.5M NaCl (v/v 10:1; RT, 3 d). The COF powder obtained shows no crystallinity, indicating that the beneficial effect of NaCl in the formation process is only present in combination with solvent structuring.

b) Effect of NaCl addition on COF crystallinity depending on introduction time

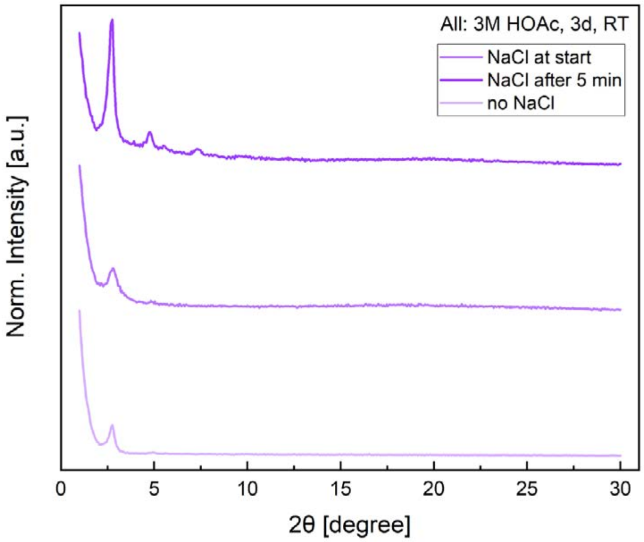

Supplementary Figure 46. Normalized PXRD patterns of TA-TAPB COF where: 1.5M NaCl was included in the catalyst mixture at the start of the reaction; an equivalent amount of NaCl was added after 5 min in a minimal amount of water (5M NaCl, 15 µl); no NaCl was included (all: 1,4-dioxane/mesitylene, v/v 9:1; 3d, RT). The crystallinity of the obtained COF powder is significantly influenced by the presence of NaCl only when it is included in the catalyst mixture from the beginning, demonstrating that the timing of the NaCl addition is critical. This indicates that the beneficial effect of the increased concentration of solute ions is correlated with the initial steps of the polymerization.

c) Relative catalyst mixture concentrations

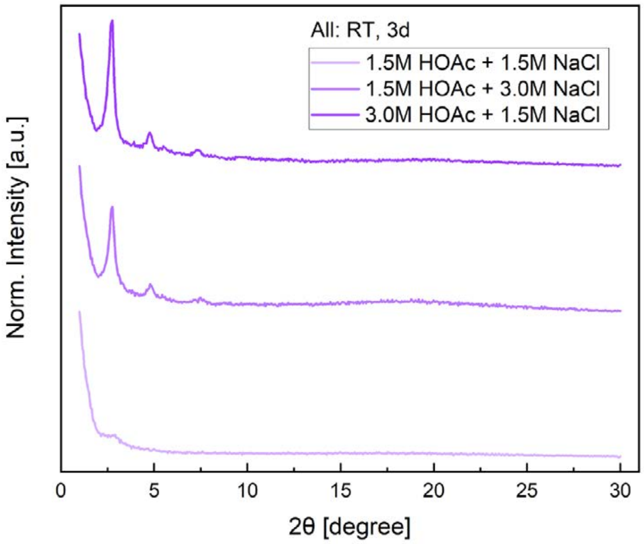

Supplementary Figure 47. Normalized PXRD patterns of TA-TAPB COF synthesized in HOAc / NaCl catalyst mixtures with different concentrations (all: 1,4-dioxane/mesitylene/aqueous catalyst mixture, v/v 9:1:1; RT, 3 d). Reduction in the amount of HOAc can be compensated for by an increase of NaCl concentration. Decreasing the HOAc concentration by half from 3M to 1.5M while keeping the NaCl constant at 1.5M results in a significant loss in crystallinity. Increasing the NaCl concentration from 1.5M to 3M while keeping the HOAc concentration at 1.5M (4-fold decrease to conventioanal 6M HOAc conditions) results in a significant increase in crystallinity.

d) Ions

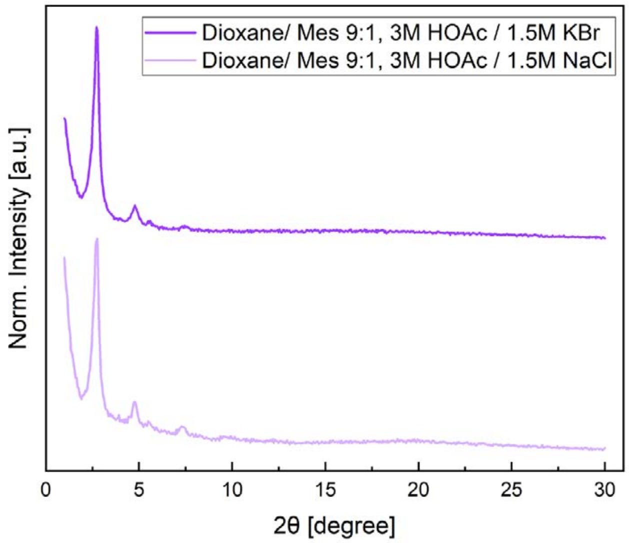

Supplementary Figure 48. Normalized PXRD patterns of TA-TAPB COF synthesized with 3M HOAc / 1.5M NaCl or 3M HOAc / 1.5M KBr, respectively (all: 1,4-dioxane/mesitylene/aqueous catalyst mixture, v/v 9:1:1; RT, 3 days). Changing the inorganic ions in the synthesis protocol from NaCl to KBr yields COF powder with comparable crystallinity.

e) Gram-scale synthesis

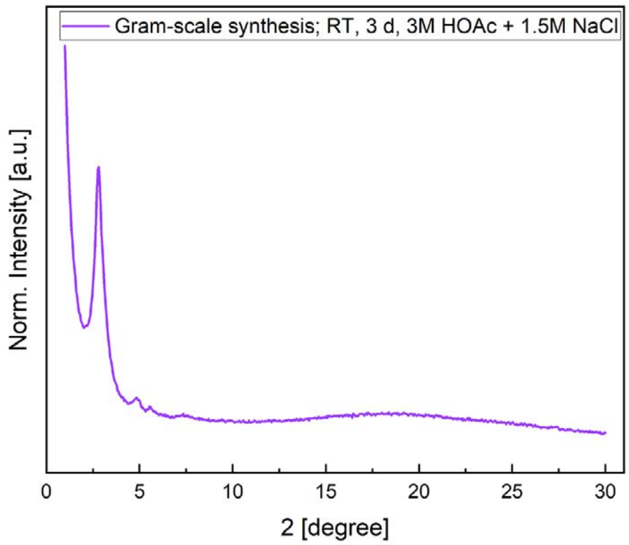

Supplementary Figure 49. Normalized PXRD pattern of TA-TAPB COF synthesized at the gram-scale (2.49 g; 86% yield) in 1,4-dioxane/mesitylene (v/v 9:1) with 3M HOAc / 1.5M NaCl as catalyst mixture (1 equiv.; RT, 3d).

f) In-situ XRD analysis of TA-TAPB COF reaction with and without NaCl

To shed light on the initial emergence of a crystalline product, we have performed in-situ XRD in a capillary for two TA-TAPB COF samples (3M HOAc + 1.5M NaCl, RT and 6M HOAc, RT). By incorporating salt in the synthesis, we register a reflection of a crystalline COF material assigned to the 100 plane as early as 13 minutes, whereas without salt and with 6M acetic acid catalyst, detection occurs at ca. 120 minutes. Additionally, in the salt-containing sample, the background notably decreases attributed to overall increase of structural order, enhancing the signal-to-noise ratio, in contrast to the constant high background detected for the 6M sample. Our findings affirm that substituting some of the acetic acid catalyst with salt promotes early development of ordered matter, without ambiguity given by the workup procedure. These results support the notion that the formed COF material exhibits fewer structural faults in the initial stages, creating a more favorable platform for the crystallization process.

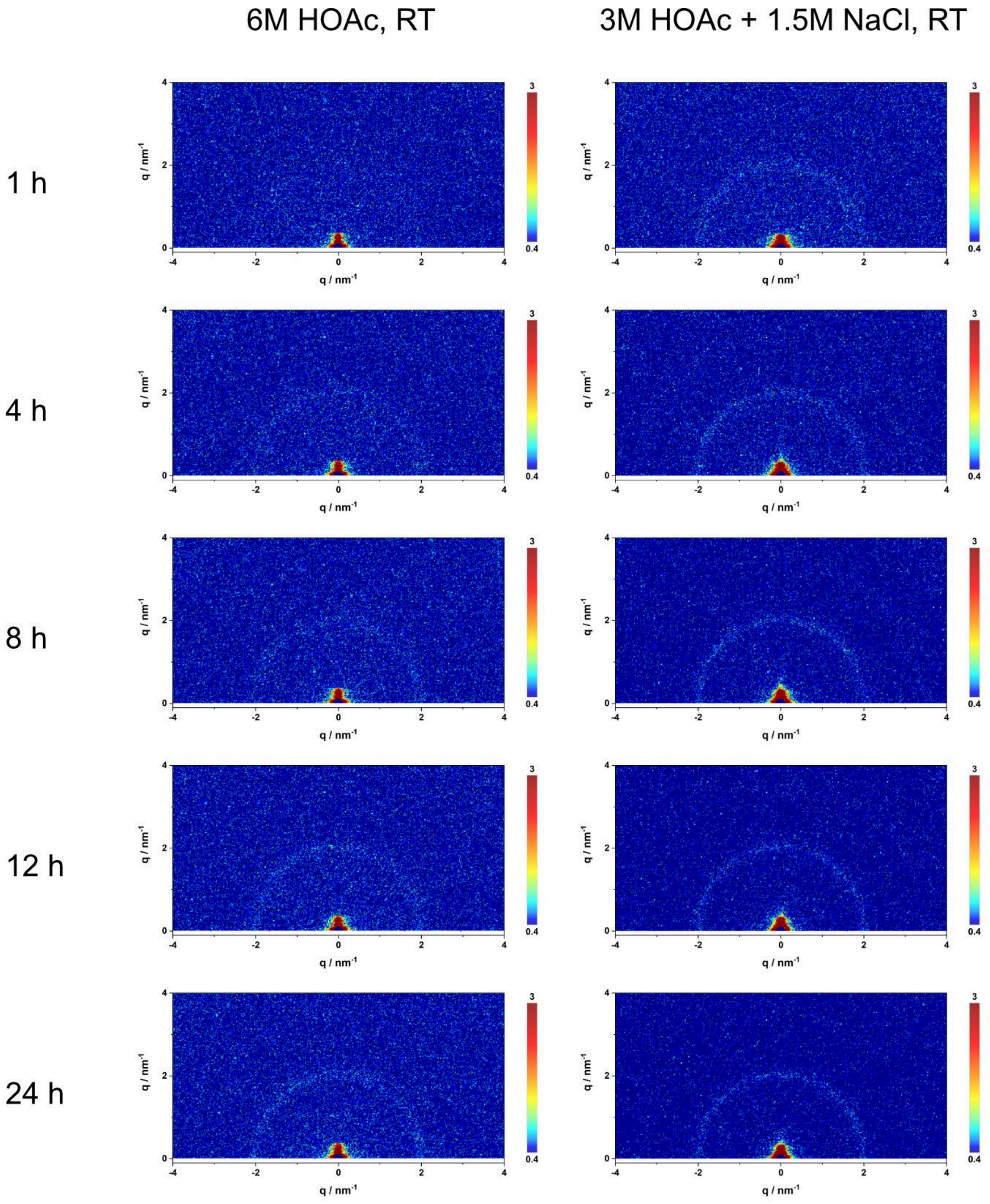

6M HOAc, RT
3M HOAc + 1.5M NaCl, RT
1 h
4 h
8 h
12 h
24 h
q / nm-1

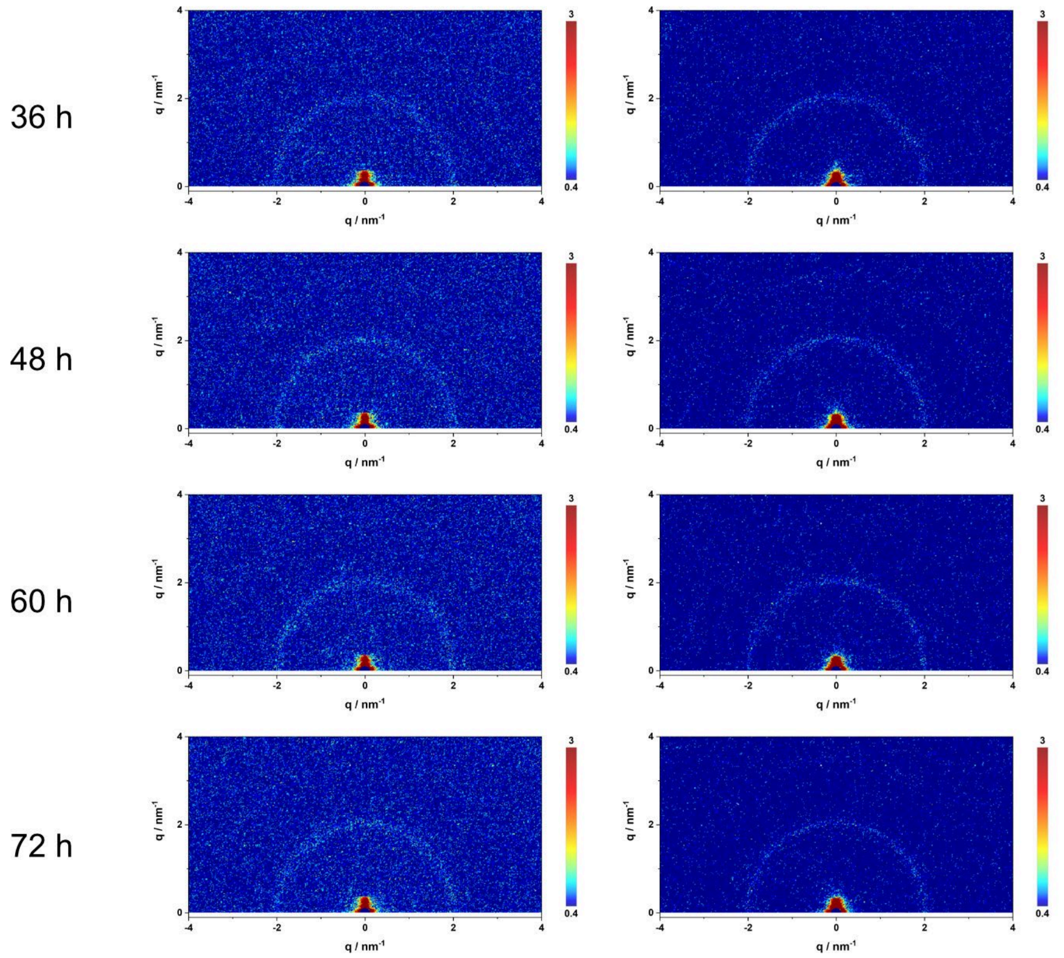

Supplementary Figure 50. In situ WAXS/SAXS 2D snapshot patterns of TA-TAPB COF synthesized in 1,4-dioxane/mesitylene (v/v 9:1) employing as aqueous catalyst (1 equiv.) either 6M HOAc (left) or 3M HOAc /1.5 M NaCl (right) at RT. Each image was recorded in the time frame of 1h.

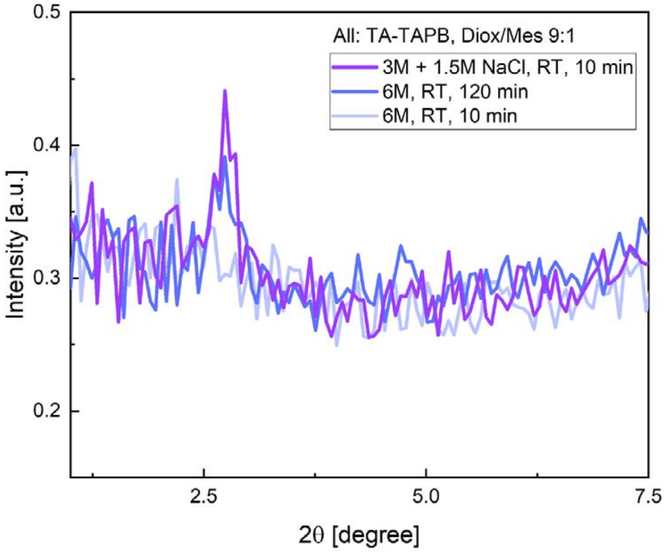

Supplementary Figure 51. Data reduction plots of XRD snapshots of the in situ WAXS/SAXS 2D analysis obtained for TA-TAPB COF synthesized with 6M HOAc and 3M HOAc / 1.5 M NaCl at different reaction time periods (all: 1,4-dioxane/mesitylene/aqueous catalyst mixture 9:1:1; RT). Each of the diffractograms was recorded in the time frame of 10 min. 3 min preparation time have to be added (see Methods).

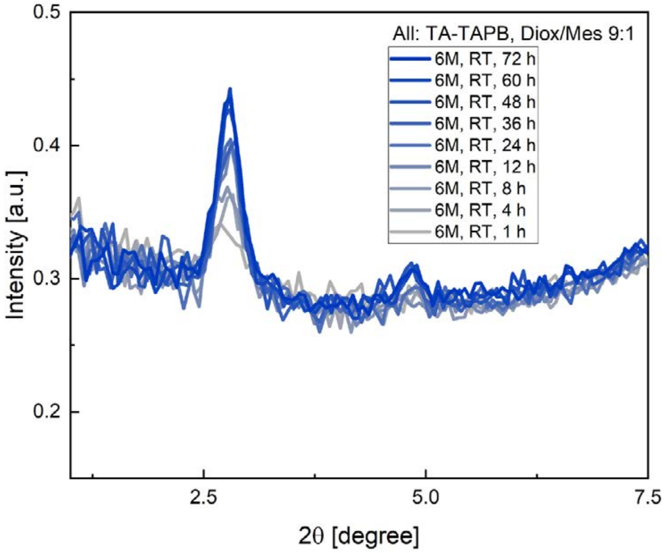

Supplementary Figure 52. Data reduction plots of XRD snapshots of the in situ WAXS/SAXS 2D analysis obtained for TA-TAPB COF synthesized with 6M HOAc at different reaction time periods (all: 1,4-dioxane/mesitylene/aqueous catalyst mixture 9:1:1; RT). Each of the diffractograms was recorded in the time frame of 1 h.

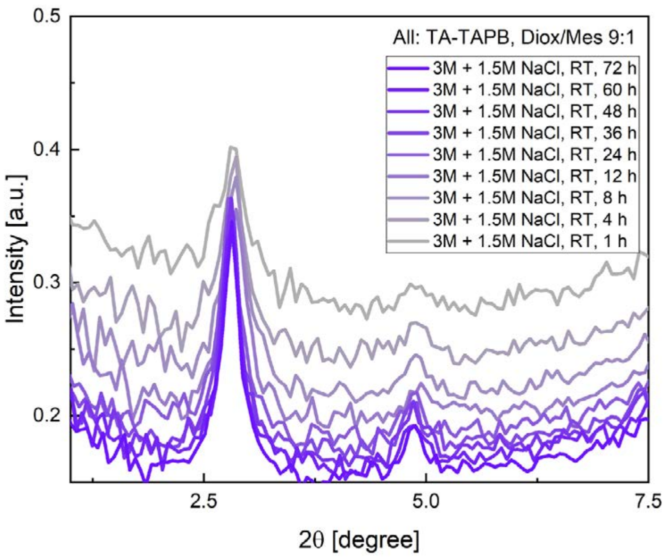

Supplementary Figure 53. Data reduction plots of XRD snapshots of the in situ WAXS/SAXS 2D analysis obtained for TA-TAPB COF synthesized with 3M HOAc / 1.5M NaCl at different reaction time periods (all: 1,4-dioxane/mesitylene/aqueous catalyst mixture 9:1:1; RT). Each of the diffractograms was recorded in the time frame of 1 h. The increasing signal-to-noise ratio is evident and attributed to the increasing number of crystalline particles in the sample.

Section 17. Expanding the concept to different COFs, solvent systems, and salt types

With the thorough insights obtained for the ion-assisted conversion protocol for TA-TAPB COF, we aimed at expanding the solvent structuring paradigm to various COF structures and solvent systems. For this we chose the synthesis of the previously reported COFs WTA,[57] TT-ETTA[58] (both synthesized in, benzyl alcohol/mesitylene/aqueous HOAc) and TAPB-DMTA[59] (1,4-dioxane/mesitylene/aqueous HOAc) (see Methods). To maintain the solvent structuring established for the TA-TAPB COF, we employed the exact solvent composition i.e., hydrotrope/hydrophobe/aqueous catalyst 9:1:1 v/v, including NaCl salt in the catalyst mixture and conducted the synthesis at room temperature for 3 days.

Importantly, the WTA COF powder obtained with the ion-assisted conversion exhibited comparable crystallinity to the material obtained at elevated temperature with the same solvent mixture. In the case of TAPB-DMTA COF, at room temperature, COF powder with high crystallinity was obtained with increased reflection intensity upon increasing salt concentration up to 1.5M. Interestingly, the very same reaction has been conducted at elevated temperatures, however under these conditions powder did not emerge. Moreover, TT-ETTA COF followed the observed trend of increasing crystallinity upon increasing salt concentration.

Notably, the optimal salt amount was found to vary for the different COF systems. This can be attributed to the specific combinations of monomers and solvents, where alterations from the system established mandatorily constitute a change in the shape of the respective phase diagram regions. To probe the influence of employing more hydrophobic solvent mixtures, we turned to yet another very typical solvent composition namely the previously reported 10:10:1 for WTA COF[57]. Here, by the addition of the aqueous catalyst solution a biphasic separation can coexist along with a structured regime (see also schematically Supplementary Figure 26[28,33,35]). The catalyst is thereby mainly confined in a relatively large volume visible to the naked eye. We postulate that breaking the large catalyst confinement droplet into smaller solvent aggregates will serve the polymerization process at room temperature both in terms of reaction medium homogeneity and in the optimum number of nucleation points. Therefore, we aimed at decreasing the water-oil surface tension, thereby decreasing the biphasic regime in the phase diagram, and giving rise to the compartmentation of the catalyst in smaller solvent aggregates. One way of achieving this is the use of a so-called antagonistic salt, e.g. $PPhCl_4$. Antagonistic salts consist of an organic group with a small inorganic counterion. The ions accumulate at oil/water interfaces, decreasing the surface tension, but do not show activity at air/water interfaces, therefore differing from surfactants.[41] Strikingly, incorporating $PPhCl_4$ into the catalyst mixture instead of an inorganic salt (i.e. NaCl) resulted in a highly crystalline WTA COF powder at room temperature.

a) WTA COF, inorganic salt

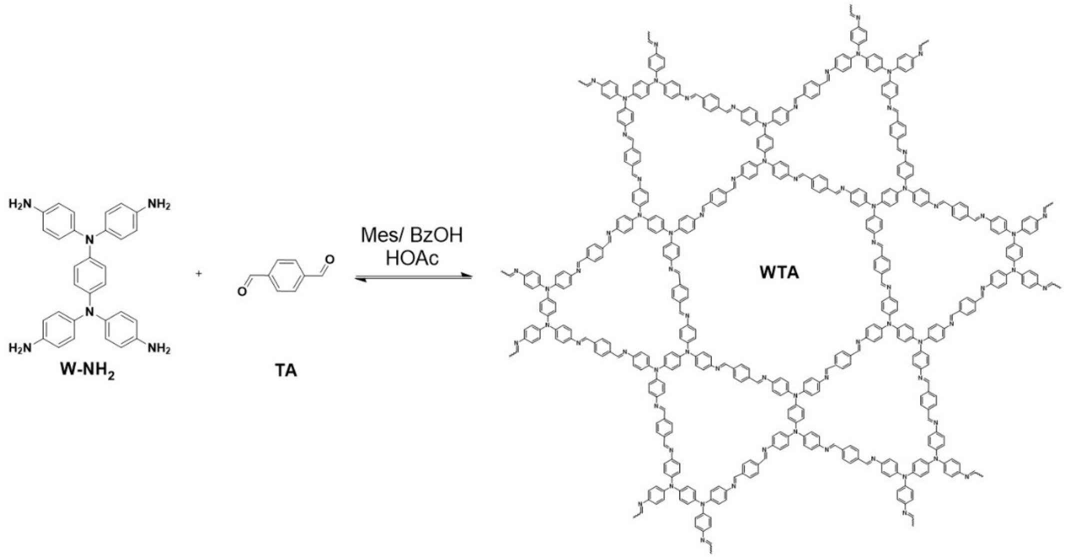

Supplementary Figure 54. Synthesis scheme of WTA COF in benzyl alcohol and mesitylene with aqueous HOAc as catalyst.

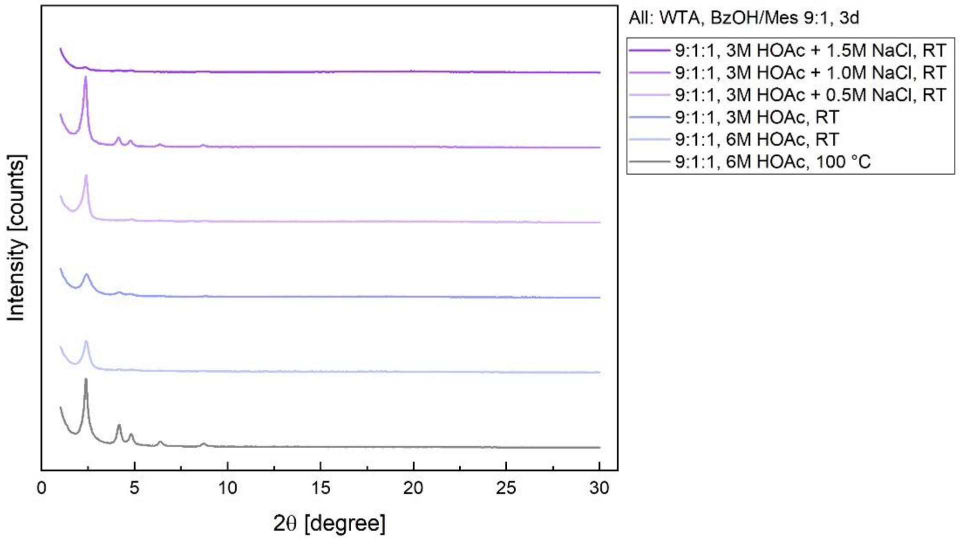

Supplementary Figure 55. PXRD patterns of WTA COF synthesized in benzyl alcohol / mesitylene with different catalyst mixtures (v/v 9:1:1) at room temperature or 100 °C for 3 days.

b) TAPB-DMTA COF, inorganic salt

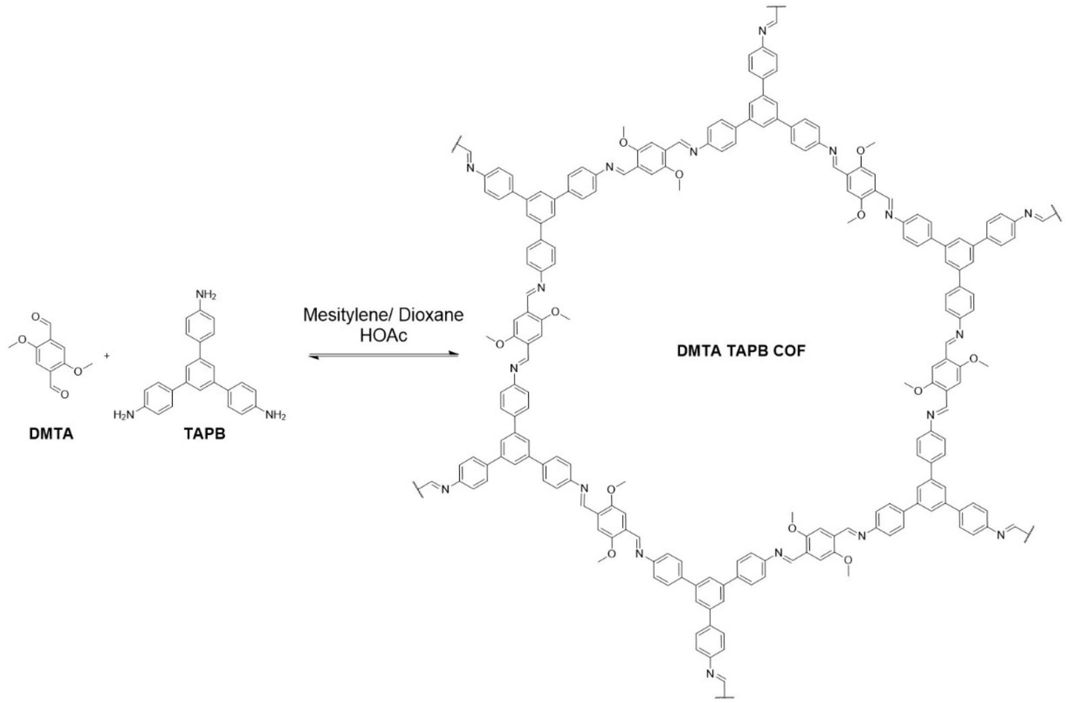

Supplementary Figure 56. Synthesis scheme of DMTA-TAPB COF in benzyl alcohol and mesitylene with aqueous HOAc as catalyst.

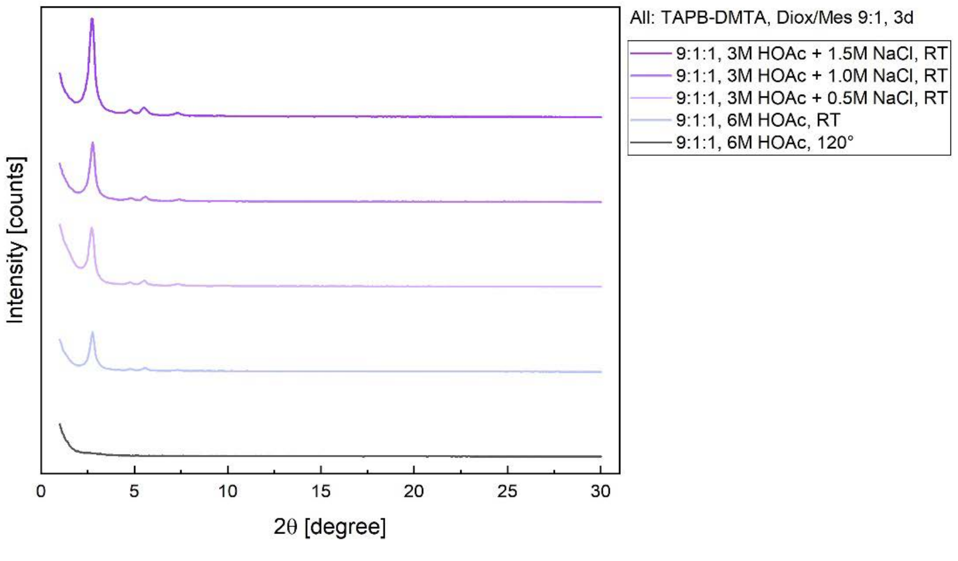

Supplementary Figure 57. PXRD patterns of TAPB-DMTA COF synthesized in 1,4-dioxane / mesitylene with different catalyst mixtures (v/v 9:1:1) at room temperature or 120 °C for 3 days.

c) TT-ETTA COF, inorganic salt

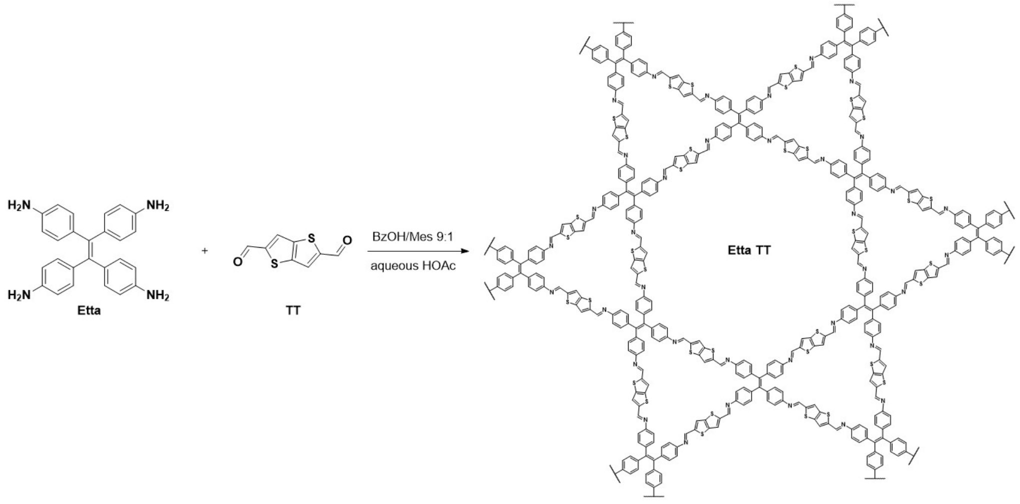

Supplementary Figure 58. Synthesis scheme of TT-ETTA COF in benzyl alcohol and mesitylene with aqueous HOAc as catalyst.

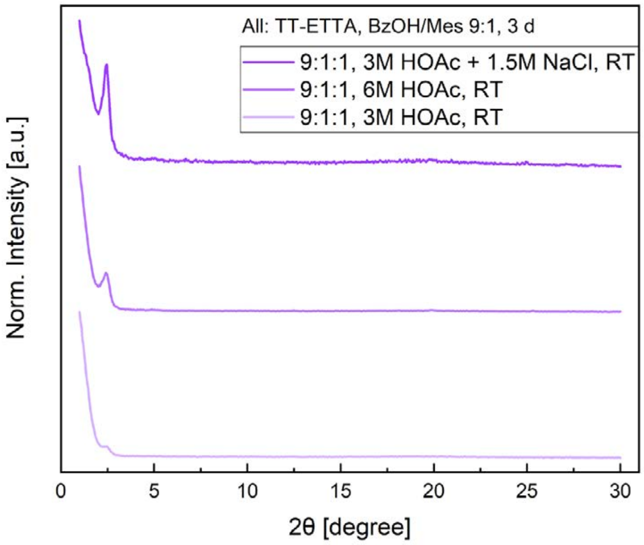

Supplementary Figure 59. Normalized PXRD patterns of TT-ETTA COF synthesized in benzyl alcohol / mesitylene with different catalyst mixtures (v/v 9:1:1) at room temperature for 3 days.

d) WTA COF, antagonistic salt

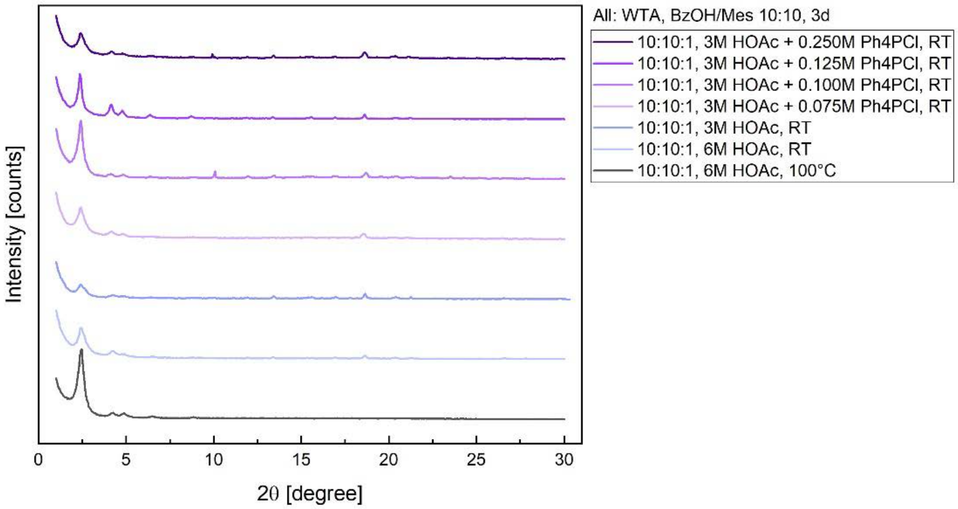

Supplementary Figure 60. PXRD patterns of WTA COF synthesized in benzyl alcohol / mesitylene with different catalyst mixtures (v/v 10:10:1) at room temperature or 100 °C for 3 days.

References

1. Sick, T. *et al.* Switching on and off Interlayer Correlations and Porosity in 2D Covalent Organic Frameworks. *J. Am. Chem. Soc.* **141**, 12570–12581 (2019).

2. Taylor, R. W. & Sandoghdar, V. Interferometric Scattering Microscopy: Seeing Single Nanoparticles and Molecules via Rayleigh Scattering. *Nano Lett.* **19**, 4827 (2019).

3. Hwang, J. & Moerner, W. E. Interferometry of a single nanoparticle using the Gouy phase of a focused laser beam. *Opt. Commun.* **280**, 487 (2007).

4. Taylor, R. W. & Sandoghdar, V. Interferometric Scattering (iSCAT) Microscopy and Related Techniques. in *Label-Free Super-Resolution Microscopy.* (ed. Astratov, V.) 25–65 (Springer, Cham., 2019). doi:10.1007/978-3-030-21722-8_2.

5. Ortega-Arroyo, J. & Kukura, P. Interferometric scattering microscopy (iSCAT): new frontiers in ultrafast and ultrasensitive optical microscopy. *Phys. Chem. Chem. Phys.* **14**, 15625 (2012).

6. Utterback, J. K. *et al.* Operando Label-Free Optical Imaging of Solution-Phase Ion Transport and Electrochemistry. *ACS Energy Lett.* **8**, 1785–1792 (2023).

7. Dastjerdi, H. M. *et al.* Optimized analysis for sensitive detection and analysis of single proteins via interferometric scattering microscopy. *J. Phys. D: Appl. Phys.* **55**, 054002 (2022).

8. Kashkanova, A. D., Blessing, M., Gemeinhardt, A., Soulat, D. & Sandoghdar, V. Precision size and refractive index analysis of weakly scattering nanoparticles in polydispersions. *Nat. Methods* 1–8 (2022) doi:10.1038/s41592-022-01460-z.

9. Taylor, R. W. *et al.* Interferometric scattering microscopy reveals microsecond nanoscopic protein motion on a live cell membrane. *Nat. Photonics* **13**, 480 (2019).

10. Fonseca, J. *et al.* Assembly of Covalent Organic Frameworks into Colloidal Photonic Crystals. *J. Am. Chem. Soc.* **145**, 20163–20168 (2023).

11. Yong, H. *et al.* Multiscale modeling and analysis for high-fidelity interferometric scattering microscopy. *J. Phys. D: Appl. Phys.* **54**, 274002 (2021).

12. Evans, A. M. *et al.* Two-Dimensional Polymers and Polymerizations. *Chem. Rev.* **122**, 442–564 (2022).

13. Li, H. *et al.* Nucleation–Elongation Dynamics of Two-Dimensional Covalent Organic Frameworks. *J. Am. Chem. Soc.* **142**, 1367–1374 (2020).

14. Li, H., Evans, A. M., Dichtel, W. R. & Bredas, J.-L. Quantitative Description of the Lateral Growth of Two-Dimensional Covalent Organic Frameworks Reveals Self-Templation Effects. *ACS Mater. Lett.* **3**, 398–405 (2021).

15. Smith, B. J., Overholts, A. C., Hwang, N. & Dichtel, W. R. Insight into the crystallization of amorphous imine-linked polymer networks to 2D covalent organic frameworks. *Chem. Commun.* **52**, 3690–3693 (2016).

16. Haase, F. & Lotsch, B. V. Solving the COF trilemma: towards crystalline, stable and functional covalent organic frameworks. *Chem. Soc. Rev.* **49**, 8469–8500 (2020).

17. Feriante, C. H. *et al.* Rapid Synthesis of High Surface Area Imine-Linked 2D Covalent Organic Frameworks by Avoiding Pore Collapse During Isolation. *Adv. Mater.* **32**, e1905776 (2020).

18. Feriante, C. *et al.* New Mechanistic Insights into the Formation of Imine-Linked Two-Dimensional Covalent Organic Frameworks. *J. Am. Chem. Soc.* **142**, 18637–18644 (2020).

19. Kang, C. *et al.* Growing single crystals of two-dimensional covalent organic frameworks enabled by intermediate tracing study. *Nat. Commun.* **13**, 1370 (2022).

20. Xue, T. *et al.* Green Synthesis of Robust Imine-Linked Two-Dimensional Covalent Organic Frameworks in Supercritical Carbon Dioxide. *Chem. Mater.* **34**, 10584–10593 (2022).

21. Tasic, A. Z., Djordjevic, B. D., Grozdanic, D. K. & Radojkovic, N. Use of mixing rules in predicting refractive indexes and specific refractivities for some binary liquid mixtures. *J. Chem. Eng. Data* **37**, 310–313 (1992).

22. Sechenyh, V. V., Legros, J.-C. & Shevtsova, V. Experimental and predicted refractive index properties in ternary mixtures of associated liquids. *J. Chem. Thermodyn.* **43**, 1700–1707 (2011).

23. Li, M., Yi, L. & Sun, C. Spontaneously formed multiscale nano-domains in monophasic region of ternary solution. *J. Colloid Interface Sci.* **628**, 223–235 (2022).

24. Polyanskiy, M. Refractive index database, H2O, D2O (Water, heavy water, ice). https://refractiveindex.info/?shelf=main&book=H2O&page=Hale (2008).

25. Polyanskiy, M. Refractive index database, C4H8O2 (Dioxane). https://refractiveindex.info/?shelf=organic&book=dioxane&page=Moutzouris (2008).

26. Sigma-Aldrich, Mesitylene reagent grade, 97%. https://www.sigmaaldrich.com/DE/de/product/aldrich/140864.

27. Polyanskiy, M. Refractive index database, SCHOTT - multiple purpose - D263TECO. https://refractiveindex.info/?shelf=glass&book=SCHOTT-multipurpose&page=D263TECO (2008).

28. Hou, W. & Xu, J. Surfactant-free microemulsions. *Curr. Opin. Colloid Interface Sci.* **25**, 67–74 (2016).

29. Hahn, M. *et al.* Ab initio prediction of structuring/mesoscale inhomogeneities in surfactant-free microemulsions and hydrogen-bonding-free microemulsions. *Phys. Chem. Chem. Phys.* **21**, 8054–8066 (2019).

30. Gradzielski, M. *et al.* Using Microemulsions: Formulation Based on Knowledge of Their Mesostructure. *Chem. Rev.* **121**, 5671–5740 (2021).

31. Diat, O. *et al.* Octanol-rich and water-rich domains in dynamic equilibrium in the pre-ouzo region of ternary systems containing a hydrotrope. *J. Appl. Crystallogr.* **46**, 1665–1669 (2013).

32. Schöttl, S. *et al.* Emergence of surfactant-free micelles from ternary solutions. *Chem. Sci.* **5**, 2949–2954 (2014).

33. Pulg, J. E., Hemker, D. L., Gupta, A., Davis, H. T. & Scriven, L. E. Interfacial tensions and phase behavior of alcohol-hydrocarbon-water-sodium chloride systems. *J. Phys. Chem.* **91**, 1137–1143 (1987).

34. Prévost, S. *et al.* Spontaneous Ouzo Emulsions Coexist with Pre-Ouzo Ultraflexible Microemulsions. *Langmuir* **37**, 3817–3827 (2021).

35. Knickerbocker, B. M., Pesheck, C. V., Davis, H. T. & Scriven, L. E. Patterns of three-liquid-phase behavior illustrated by alcohol-hydrocarbon-water-salt mixtures. *J. Phys. Chem.* **86**, 393–400 (1982).

36. Knickerbocker, B. M., Pesheck, C. V., Scriven, L. E. & Davis, H. T. Phase behavior of alcohol-hydrocarbon-brine mixtures. *J. Phys. Chem.* **83**, 1984–1990 (1979).

37. Winsor, P. A. Hydrotropy, solubilisation and related emulsification processes. *Trans. Faraday Soc.* **44**, 376–398 (1948).

38. Li, M., Wakata, Y., Zeng, H. & Sun, C. On the thermal response of multiscale nanodomains formed in trans-anethol/ethanol/water surfactant-free microemulsion. *J. Colloid Interface Sci.* **652**, 1944–1953 (2023).

39. Zhang, Y. *et al.* Temperature-Switchable Surfactant-Free Microemulsion. *Langmuir* **36**, 7356–7364 (2020).

40. Keiser, B. A., Varie, D., Barden, R. E. & Holt, S. L. Detergentless water/oil microemulsions composed of hexane, water, and 2-propanol. 2. Nuclear magnetic resonance studies, effect of added sodium chloride. *J. Phys. Chem.* **83**, 1276–1280 (1979).

41. Michler, D., Shahidzadeh, N., Westbroek, M., Roij, R. van & Bonn, D. Are Antagonistic Salts Surfactants? *Langmuir* **31**, 906–911 (2015).

42. Schöttl, S. & Horinek, D. Salt effects in surfactant-free microemulsions. *J. Chem. Phys.* **148**, 222818 (2018).

43. Sadakane, K. *et al.* 2D-Ising-like critical behavior in mixtures of water and 3-methylpyridine including antagonistic salt or ionic surfactant. *Soft Matter* **7**, 1334–1340 (2010).

44. Iglicki, D., Goubault, C., Mahamoud, M. N., Chevance, S. & Gauffre, F. Shedding light on the formation and stability of mesostructures in ternary “Ouzo” mixtures. *J. Colloid Interface Sci.* **633**, 72–81 (2023).

45. Rak, D. & Sedlák, M. On the Mesoscale Solubility in Liquid Solutions and Mixtures. *J. Phys. Chem. B.* **123**, 1365–1374 (2019).

46. Zemb, T. N. *et al.* How to explain microemulsions formed by solvent mixtures without conventional surfactants. *Proc. Natl. Acad. Sci. U.S.A.* **113**, 4260–4265 (2016).

47. Krickl, S. *et al.* A systematic study of the influence of mesoscale structuring on the kinetics of a chemical reaction. *Phys. Chem. Chem. Phys.* **19**, 23773–23780 (2017).

48. Zhou, Y., He, S., Li, H. & Zhang, Y. CO2 and Temperature Control over Nanoaggregates in Surfactant-Free Microemulsion. *Langmuir* **37**, 1983–1990 (2021).

49. Sun, B. *et al.* A surfactant-free microemulsion consisting of water, ethanol, and dichloromethane and its template effect for silica synthesis. *J. Colloid Interface Sci.* **526**, 9–17 (2018).

50. Iglicki, D. *et al.* Simple elaboration of drug-SPION nanocapsules (hybridosomes®) by solvent shifting: Effect of the drug molecular structure and concentration. *Int. J. Pharm.* **649**, 123645 (2024).

51. Klossek, M. L., Touraud, D. & Kunz, W. Eco- solvents – cluster-formation, surfactantless microemulsions and facilitated hydrotropy. *Phys. Chem. Chem. Phys.* **15**, 10971–10977 (2013).

52. Cai, S.-L. *et al.* An unprecedented 2D covalent organic framework with an htb net topology. *Chem. Commun.* **55**, 13454–13457 (2019).

53. Nguyen, H. L. *et al.* A Porous Covalent Organic Framework with Voided Square Grid Topology for Atmospheric Water Harvesting. *J. Am. Chem. Soc.* **142**, 2218–2221 (2020).

54. Liu, Y., Xu, J., Deng, H., Song, J. & Hou, W. A surfactant-free microemulsion composed of isopentyl acetate, n -propanol, and water. *RSC Adv.* **8**, 1371–1377 (2018).

55. Fischer, V., Marcus, J., Touraud, D., Diat, O. & Kunz, W. Toward surfactant-free and water-free microemulsions. *J. Colloid Interface Sci.* **453**, 186–193 (2015).

56. Thommes, M. *et al.* Physisorption of gases, with special reference to the evaluation of surface area and pore size distribution (IUPAC Technical Report). *Pure Appl. Chem.* **87**, 1051–1069 (2015).

57. M., R., Julian *et al.* Highly conducting Wurster-type twisted covalent organic frameworks. *Chem. Sci.* **11**, 12843 (2020).

58. Ascherl, L. *et al.* Molecular docking sites designed for the generation of highly crystalline covalent organic frameworks. *Nat. Chem.* **8**, 310–316 (2016).

59. Ji, W. *et al.* Solvothermal depolymerization and recrystallization of imine-linked two-dimensional covalent organic frameworks. *Chem. Sci.* **12**, 16014–16022 (2021).

60. Smith, B. J. *et al.* Colloidal Covalent Organic Frameworks. *ACS Cent. Sci.* **3**, 58–65 (2017).

61. Parent, L. R. *et al.* Tackling the Challenges of Dynamic Experiments Using Liquid-Cell Transmission Electron Microscopy. *Acc. Chem. Res.* **51**, 3–11 (2018).

62. Zheng, W. & Lee, L. Y. S. Observing Electrocatalytic Processes via In Situ Electrochemical Scanning Tunneling Microscopy: Latest Advances. *Chem. Asian J.* **17**, e202200384 (2022).

63. Mirsaidov, U., Patterson, J. P. & ZhengGuest, H. Liquid phase transmission electron microscopy for imaging of nanoscale processes in solution. *MRS Bull.* **45**, 704–712 (2020).

64. Venugopal, A. *et al.* Caught in Action: Visualizing Dynamic Nanostructures Within Supramolecular Systems Chemistry. *Angew. Chem. Int. Ed.* **62**, e202208681 (2022).

65. Zhan, G. *et al.* Observing polymerization in 2D dynamic covalent polymers. *Nature* **603**, 835–840 (2022).

66. Merryweather, A. J., Schnedermann, C., Jacquet, Q., Grey, C. P. & Rao, A. Operando optical tracking of single-particle ion dynamics in batteries. *Nature* **594**, 522–528 (2021).

67. Bruycker, K. D., Welle, A., Hirth, S., Blanksby, S. J. & Barner-Kowollik, C. Mass spectrometry as a tool to advance polymer science. *Nat. Rev. Chem.* **4**, 257–268 (2020).

68. Xia, M. *et al.* Lab-Scale In Situ X-Ray Diffraction Technique for Different Battery Systems: Designs, Applications, and Perspectives. *Small Methods* **3**, 1900119 (2019).

69. Smith, B. J. & Dichtel, W. R. Mechanistic Studies of Two-Dimensional Covalent Organic Frameworks Rapidly Polymerized from Initially Homogenous Conditions. *J. Am. Chem. Soc.* **136**, 8783–8789 (2014).

70. Omar, A. F. B. & MatJafri, M. Z. B. Turbidimeter Design and Analysis: A Review on Optical Fiber Sensors for the Measurement of Water Turbidity. *Sensors* **9**, 8311–8335 (2009).

71. Llewellyn, A. V., Matruglio, A., Brett, D. J. L., Jervis, R. & Shearing, P. R. Using In-Situ Laboratory and Synchrotron-Based X-ray Diffraction for Lithium-Ion Batteries Characterization: A Review on Recent Developments. *Condens. Matter* **5**, 75 (2020).
